# A Computational Method for Classifying Different Human Tissues with Quantitatively Tissue-Specific Expressed Genes

**DOI:** 10.3390/genes9090449

**Published:** 2018-09-07

**Authors:** JiaRui Li, Lei Chen, Yu-Hang Zhang, XiangYin Kong, Tao Huang, Yu-Dong Cai

**Affiliations:** 1 School of Life Sciences, Shanghai University, Shanghai 200444, China; jiaruili@shu.edu.cn; 2 College of Information Engineering, Shanghai Maritime University, Shanghai 201306, China; chen_lei1@163.com; 3 Shanghai Key Laboratory of PMMP, East China Normal University, Shanghai 200241, China; 4 Institute of Health Sciences, Shanghai Institutes for Biological Sciences, Chinese Academy of Sciences, Shanghai 200031, China; zhangyh825@163.com

**Keywords:** tissue-specific expressed genes, transcriptome, tissue classification, support vector machine, feature selection

## Abstract

Tissue-specific gene expression has long been recognized as a crucial key for understanding tissue development and function. Efforts have been made in the past decade to identify tissue-specific expression profiles, such as the Human Proteome Atlas and FANTOM5. However, these studies mainly focused on “qualitatively tissue-specific expressed genes” which are highly enriched in one or a group of tissues but paid less attention to “quantitatively tissue-specific expressed genes”, which are expressed in all or most tissues but with differential expression levels. In this study, we applied machine learning algorithms to build a computational method for identifying “quantitatively tissue-specific expressed genes” capable of distinguishing 25 human tissues from their expression patterns. Our results uncovered the expression of 432 genes as optimal features for tissue classification, which were obtained with a Matthews Correlation Coefficient (*MCC*) of more than 0.99 yielded by a support vector machine (SVM). This constructed model was superior to the SVM model using tissue enriched genes and yielded *MCC* of 0.985 on an independent test dataset, indicating its good generalization ability. These 432 genes were proven to be widely expressed in multiple tissues and a literature review of the top 23 genes found that most of them support their discriminating powers. As a complement to previous studies, our discovery of these quantitatively tissue-specific genes provides insights into the detailed understanding of tissue development and function.

## 1. Introduction

A biological tissue is an ensemble of similar cells residing in the same location and performing specific biological functions in multicellular organisms. As the bridge between single cells and functional organs, tissues are elementary units with both phenotypical and functional contributions to biological identity [1]. All biological functions are regulated and manipulated directly or indirectly by proteins, which can be further attributed to gene expression patterns measured by messenger RNA (mRNA) expression [2]. Therefore, different tissues and cell types could have their own unique expression patterns and a full picture of how genes are expressed in different tissues will help to unveil the molecular mechanisms involved in tissue development and function.

Ten years ago, two milestones for identifying tissue-specific gene expression were conducted and completed right after the human genome project, which built tissue-specific gene expression profiles at the protein and RNA levels [3,4]. At the protein level, the protein distribution in human tissues was explored using 718 antibodies corresponding to 650 human protein-coding genes in the Human Protein Atlas [3]. While at the RNA level, the FANTOM consortium initiated the creation of a gene atlas by integrating mouse and human expression data from multiple tissues using microarrays [4] and built the widely used BioGPS portal, which has expression data from numerous resources [5]. These projects have been extended and updated by incorporating more genes and taking advantage of advanced technologies, such as next-generation sequencing. For example, the number of genes included in the Human Protein Atlas project has been increased to 10,118, which is more than half of the protein-coding genes in humans [6]. Meanwhile, the application of RNA-Seq technologies has created an RNA-Seq Atlas in different human tissues, providing a more comprehensive and unbiased view of gene expression compared with the initial efforts using microarrays [7]. A recent study presented a map of the human tissue proteome by integrating multiple-omics approaches, including RNA-Seq and tissue microarray-based immunohistochemistry, which detected more than 90% of putative protein-coding genes and found that 2355 genes are significantly enriched in a single tissue, 3478 are enhanced in a single tissue and 1109 are enriched in a group of tissues [8]. These tissue-specific genes not only help understand human biology but can also be applied in medical research, such as pharmaceutical drug development and biomarker discovery in the field of translational medicine. However, these studies have mainly focused on “qualitatively tissue-specific expressed genes”, which are enriched in a single or subgroup of tissues. The genes expressed in all or almost all tissues could also have divergent expression patterns among different cell types, which we termed as “quantitatively tissue-specific expressed genes”. Although these quantitatively tissue-specific expressed genes have less expression enrichment compared with qualitatively expressed genes, they might also play important roles in tissue function and development.

In this study, we took advantage of recently published transcriptomic data in multiple tissues from the Genotype-Tissue Expression (GTEx) project [9] and present a new computational method that integrates machine learning algorithms to identify genes that are widely expressed in the human body but with different expression signatures across 25 human tissues and are capable of distinguishing different tissue types. According to our results, the 25 tissues have been taken into account and subtyped by 432 key genes using a prediction engine based on a support vector machine (SVM) [10,11] with an Matthews Correlation Coefficient (*MCC*) value more than 0.99, revealing the detailed expression characteristics of different tissue subtypes. The constructed classification model also had good generalization ability because it yielded *MCC* of 0.985 on an independent test dataset. In addition, the superiority of this model was proved by comparing it to the SVM model using tissue enriched genes. A detailed analysis was also performed on the 23 most important genes among 432 key genes. As a complement to previous studies, our results demonstrate the ability to classify tissues through a series of quantitatively tissue-specific expressed genes, suggesting that these genes could also play important roles in tissue development and function.

## 2. Materials and Methods

### 2.1. Dataset

The expression profiles from different tissue samples obtained by RNA-Seq were downloaded from GTEx V6p (http://gtexportal.org/home/datasets) [9]. Tissues with sample sizes smaller than 80 were excluded, resulting in a total of 8436 samples from 25 tissues. These samples comprised a training dataset. For an easy description, each tissue was denoted as *T_i_* (*I* = 1, 2, …, 25). For the computational analysis, we extracted expression levels of 18,365 genes that are expressed (i.e., expression level was not zero) in at least one of 8436 samples from the file “GTEx_Analysis_v6p_RNA-seq_RNA-SeQCv1.1.8_gene_rpkm.gct”; that is, each sample was represented by 18,365 features.

Besides, an independent test dataset was constructed using the new samples added in GTEx v7 after GTEx v6p. This dataset included 3367 samples from the same 25 tissues. These 25 tissues and their sample sizes in training and independent test datasets are listed in Table 1.

### 2.2. Feature Ranking and Selection

The purpose of this study is to extract a group of genes that can contribute to tissue classification based on gene expression levels. To do that, the minimum redundancy maximum relevance (mRMR) method [12] proposed by Peng et al. was employed to analyze all the features and yield a feature list, named the mRMR feature list. This feature selection method has been applied to tackle various biological problems [13,14,15,16,17,18,19,20,21,22,23,24,25]. In the method, the discriminated power of a feature *f* is reflected by the relevance between it and a target class *c*, which is measured from their mutual information (*MI*). The *MI* can be evaluated according to the following equation (*x* and *y* represent two variables):

(1) $$\iint p\left(x,y\right)log\frac{p\left(x,y\right)}{p\left(x\right)p\left(y\right)}dxdy$$

where *p*(*x*) and *p*(*y*) are the marginal probabilistic density of *x* and *y* and *p*(*x*, *y*) is their joint probabilistic density. Additionally, the redundancy between two features *f*_1_ and *f*_2_ is also evaluated by their *MI*. To produce the mRMR feature list, the mRMR method repeatedly selects a non-selected feature that has a maximum relevance to the target and a minimum redundancy to the already selected features. The mRMR feature list ranks all the features according to their selection order. For the formulation, this list was denoted as follows:

(2) $$F=\left[f_{1},f_{2},\ldots f_{N}\right]$$

where *N* is the total number of features (*N* = 18,365 in this study). It is clear that the top features in this list are more important for identifying tissue types.

The mRMR method only provides a feature list, in which important features receive high ranks. However, we still do not know which features should be selected for classification. In view of this, the incremental feature selection (IFS) method was adopted in this study to select a group of features that can effectively classify the human tissues. In this method, a series of feature sets, denoted as $F_{1}$, $F_{2}$, …, $F_{i}$, …, $F_{N}$, were constructed according to the mRMR feature list *F*, where the subscript *i* in *F_i_* represents the top *i* features in *F* comprising the feature set *F_i_*, that is,

(3) $$F_{i}=\left[f_{1},f_{2},\ldots f_{i}\right]$$

Then, a classification model would be built on each feature set trained by a machine learning algorithm (e.g., SVM). Accordingly, the classification model yielding the best performance can be found and termed the optimal classification model. Features used in this optimal classification model comprise the optimal feature set. In this study, there were 18,365 features in total, meaning we could construct 18,365 classification models. However, due to our limited computational power, this would be time-consuming. Thus, we only examined feature sets that contained 4–500 features to construct the classification models and selected the model yielding the highest Matthews Correlation Coefficient, a powerful measurement to evaluate the performance of different models. For convenience, the obtained model was called optimal classification model.

### 2.3. Classification Algorithm

In the IFS method, a machine learning algorithm was used to build a classification model based on each feature set. Here, we selected one of the most classic machine learning algorithms, SVM [10,11]. The principle of SVM is to find a balance between the learning error and the minimum statistical risk. To date, several types of SVMs have been proposed to address different kinds of problems. In this study, we adopted an SVM that is trained by sequential minimum optimization (SMO) [26], which always breaks the quadratic programming (QP) problem into a series of the smallest possible sub-QP problems. Then, these sub-QP problems are solved analytically, thereby avoiding matrix storage and using numeric QP optimization steps. To quickly implement this type of SVM, a tool named “SMO” was employed in Weka (https://www.cs.waikato.ac.nz/ml/weka/) [27] and it was executed using its default settings, where the kernel was set as polynomial function and the tolerance parameter was set to 0.001.

### 2.4. Measurements

The prediction abilities of the constructed classification models were evaluated by a 10-fold cross-validation (10-CV) [16,28,29,30], which yields similar results to the stricter test called the Jackknife cross-validation (J-CV) [31,32] but saves significant computational resources.

In this study, 25 tissues were considered. Thus, based on the predicted results derived from the SVM on different feature sets and evaluated by 10-CV, we can compute the prediction accuracy for *j*-th tissue *T_j_*, which was defined as follows:

(4) $$ACC_{j}=\frac{n_{j}}{N_{j}},\;j\;=\;1,\;2,\;\ldots ,\;25$$

where *n_j_* represents the number of correctly predicted samples in *T_j_* and *N_j_* represents the total number of samples in *T_j_*. We can further compute the overall accuracy (*TACC*) with the following formula:

(5) $$TACC=\frac{\sum_{j=1}^{25}n_{j}}{\sum_{j=1}^{25}N_{j}}$$

We can see from Table 1 that some tissues were large (e.g., brain), while some tissues had much fewer samples (e.g., uterus). The overall accuracy may strongly rely on the prediction accuracy of the tissues with large sizes. Thus, it is not proper to evaluate the performance of models using *TACC*. In binary classification, *MCC* [33] is deemed as a balanced measurement even if the class sizes are of great differences. Later, the multi-class version—called *MCC* in multiclass—was developed by Gorodkin [34], which inherits the merits of original *MCC*. Here, we give a brief description of the *MCC* in multiclass. A more detailed description can be found in Gorodkin’s study [34].

Given a classification problem involving *n* samples, denoted by $s_{1},s_{2},\ldots ,s_{n}$ and *N* classes, represented by 1, 2, …, *N*. The true classes of all the samples can be formulated by the matrix $Y={\left(y_{ij}\right)}_{n\times N}$, where $y_{ij}=1$ if $s_{i}$ belongs to class *j*; otherwise, it is set to 0. For the predicted classes of all the samples, the classes can be used to a define another matrix, $X={\left(x_{ij}\right)}_{n\;\times \;N}$, which can be defined as follows: $x_{ij}=1$, if $s_{i}$ is predicted to be in class *j*, otherwise, $x_{ij}=0$. Then, the *MCC* in the multiclass is defined as follows:

(6) $$MCC=\frac{cov\left(X,Y\right)}{\sqrt{cov\left(X,X\right)cov\left(Y,Y\right)}}$$

where *cov*(*X*,*Y*) is the covariance function of *X* and *Y*, which can be computed with the following formula:

(7) $$\mathrm{cov}\left(X,Y\right)=\frac{1}{N}\overset{N}{\underset{k=1}{\sum }}cov\left(x_{k},y_{k}\right)=\frac{1}{N}\overset{N}{\underset{k=1}{\sum }}\overset{n}{\underset{i=1}{\sum }}\left(x_{ik}-\bar{x_{k}}\right)\left(y_{ik}-\bar{y_{k}}\right)$$

where $x_{k}$ and $y_{k}$ denote the *k*-th column of *X* and *Y*, respectively and $\bar{x_{k}}$ and $\bar{y_{k}}$ denote the mean values of the numbers in $x_{k}$ and $y_{k}$, respectively.

Like the original *MCC* value proposed by Matthews [33], the range of the *MCC* in the multiclass is between −1 and 1, where the higher the *MCC* value obtained, the better a classifier is (1 means the given classifier yields a perfect classification, 0 indicates a classification is no better than a random prediction and −1 represents a total misclassification). In this study, we used the *MCC* values in the multiclass as the key measurements and only called *MCC* in the following text for convenience.

## 3. Results

### 3.1. Results of Feature Ranking

To avoid the unbalanced data sizes among the different tissues, we collected the transcriptome data from 8436 samples originating from 25 tissues in the GTEx project [9]; tissues with sample sizes smaller than 80 were excluded from this study. To perform a comprehensive analysis, all 18,365 genes expressed in the 8436 samples, regardless of tissue specificity or expression, were used to construct the features. All the features were rigorously analyzed with the widely used mRMR method, inducing the mRMR feature list (Supplementary Material S1).

### 3.2. Results of Feature Selection

**Figure 1:**
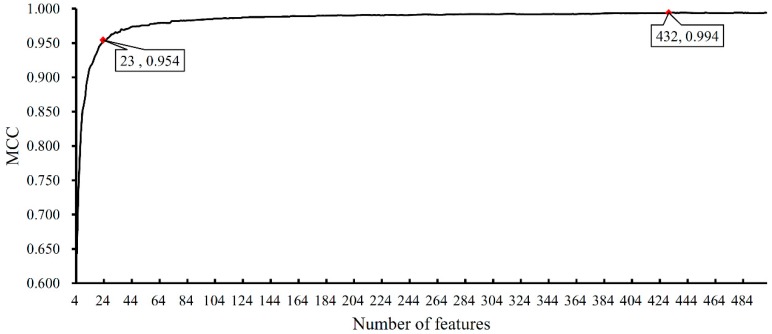
The incremental feature selection (IFS) curve illustrating the performance of the classification models using different numbers of features. Red diamonds represent the performance when the top 23 genes and 432 features were used for building the classification models.

**Figure 2:**
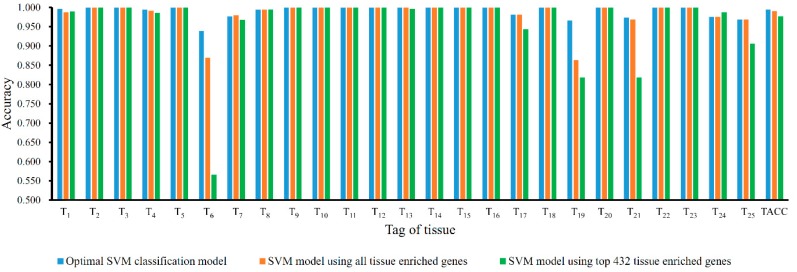
The performance of the optimal support vector machine (SVM) classification model, SVM model using all tissue enriched genes and SVM model using top 432 tissue enriched genes, including accuracy on each tissue and overall accuracy. The optimal SVM classification model gave better performance.

The obtained mRMR feature list was used with the IFS method to discover the optimal feature set for the SVM. However, because of our limited computational power, we only tried feature sets $F_{4},F_{5},\ldots ,F_{500}$. For each feature set, an SVM-based classification model was built, in which each sample was represented by the features in the set. Then, 10-CV was adopted to evaluate the performance of each classification model; the predicted results included the prediction accuracies of the 25 tissues, *TACC* and *MCC* (Supplementary Material S2). To clearly show the relationship between the number of used features and the performance of the corresponding classification model, the IFS curve was plotted and is shown in Figure 1 with the feature number as the X-axis and the *MCC* value as the Y-axis. The highest *MCC* value (0.994) reached almost 1.00 and was obtained when the top 432 features in the mRMR feature list were used, suggesting that these 432 features could be optimal features for the SVM for distinguishing the 25 tissues. Accordingly, the optimal classification model using 432 optimal features and SVM can be built. The detailed performance of this model, including accuracy on each tissue and *TACC*, is shown in Figure 2, from which we can see that 17 of 25 tissues received a perfect classification (*ACC_j_* = 1) and all accuracies are higher than 0.930, suggesting that the performance of the optimal classification model is quite stable on different tissues.

To examine whether these 432 genes are widely expressed or tissue-specific, we searched their annotations in the Human Protein Atlas [8]. Among the 432 genes, fourteen genes were annotated as tissue-enriched, tissue-enhanced or group-enriched, which suggests that these fourteen genes might be tissue-specific or group-specific (Supplementary Material S3). However, further examination revealed that only one gene, *ASAP2*, is annotated as tissue-enriched (in testis) in the GTEx data and that this gene shows no enriched or enhanced expression in the FANTOM5 database [35], another expression atlas for RNA levels. Both the GTEx and FANTOM5 annotations were obtained through the Human Protein Atlas database to keep the criteria consistent. Therefore, these 432 genes showed the ability to distinguish different tissues that are widely expressed across multiple tissues.

### 3.3. Comparison of SVM Model with Tissue Enriched Genes

As mentioned in Section 3.2, an SVM classification model was built to classify samples into 25 tissues, which yielded the *MCC* of 0.994. To indicate its effectiveness, we mapped the tissue enriched proteins retrieved from Human Protein Atlas onto the filtered GTEx expression dataset which excluded the non-expressed genes, resulting in 1981 genes (see Supplementary Material S4). The expression levels of these genes were used to represent each sample in the training dataset. Then, the SVM was executed on this data with its performance evaluated by 10-CV. The predicted results were counted as *MCC* of 0.990, which was lower than that obtained by the optimal SVM classification model. For detailed comparison, the detailed performance of these two models, including accuracy on each tissue and *TACC*, is illustrated in Figure 2. It can be observed that the optimal SVM classification model gave better or equal performance on almost all tissues (expect tissue *T*_7_, “Colon”) than SVM model using tissue enriched genes.

Besides, considering that the above two models using different number of genes, to give a fairer comparison, we also evaluated the importance of 1981 tissue enriched genes via mRMR method and extracted the top 432 genes to construct an SVM classification model and evaluate it on the training dataset, yielding *MCC* of 0.976, which was much lower than that obtained by the optimal SVM classification model. The detailed performance on each tissue and overall accuracy is shown in Figure 2, from which we can see that the optimal SVM classification model gave higher or equal accuracies on almost all tissues expect one tissue (*T*_24_, “Uterus”).

All these suggested that important genes for classification of samples into different tissues can be extracted via advanced computational methods used in this study and they can be adopted to build a better classification model.

### 3.4. Performance of the Optimal SVM Classification Model on Test Dataset

We constructed an optimal SVM classification model in Section 3.2 via mRMR and IFS methods. To test its generalization, we performed this model on the independent test dataset. The obtained *TACC* and *MCC* were 0.986 and 0.985, respectively, indicating that the prediction ability of the constructed model was quite strong. We also calculated the *MCC* of tissue enriched genes on the independent test dataset and it was 0.972, smaller than the *MCC* (0.985) obtained by the optimal SVM classification model.

### 3.5. Comparison of SVM Model with t-Test Genes

Beside the machine learning algorithms used in present study, there were other ways to identify the quantitatively tissue-specific expressed genes. Another more straightforward way was to identify the significantly highly expressed genes in each tissue by performing t test between one tissue and all the other tissues and then combine the highly expressed genes in each tissue to get the final quantitatively tissue-specific expressed genes. To make the t test result comparable with our result, we selected the top 19 significantly highly expressed genes from 25 tissues and obtained 422 unique *t*-test genes (see Supplementary Material S5), which had similar number of genes with the 432 optimal genes. We evaluated the performance of the 422 *t*-test genes on independent test dataset and its *MCC* was 0.984, slightly smaller than the *MCC* obtained by 432 optimal genes, 0.985. It was difficult to tell whether the increase of *MCC* from 0.984 to 0.985 was statistically significant, especially when the *MCC* was already so high and there was little space for improvement. But we strongly believe that the quantitative gene expression signatures identified either by *t*-test based method or mRMR and IFS based method, are better than traditional tissue specific gene lists that only consider the expression or not rather than the expression level differences. The results on the high-quality dataset GTEx had supported it. Although the GTEx project has ended, the Enhancing GTEx (eGTEx) project [36] carries on. We will work closely with the eGTEx Consortium to test the constructed model on larger new samples and update the model correspondingly.

## 4. Discussion

As we have analyzed above, RNA-Seq has been reported to be an effective classification tool for identifying cell types. Based on the transcriptome datasets from different human tissues presented in a recent study, we developed a new computational method and successfully identified 432 quantitatively tissue-specific expressed genes capable of classifying twenty-five human tissues with high accuracy (*MCC* > 0.99). To further examine the reliability of our results, we selected the top 23 genes in the mRMR feature list for detailed analyses (Table 2). The performance of the classification model built on these genes displayed an *MCC* value > 0.95 (Figure 1, Supplementary Material S2), suggesting the significant role of these genes among the 432 genes in the classification. As shown in Table 2, we searched the 23 genes in Human Protein Atlas [8] and Expression Atlas [37]. Based on Human Protein Atlas, 16 genes were “Expressed in all”; six genes were “Mixed”; only one gene was “Tissue enhanced (thyroid gland)”. Based on Expression Atlas, all genes were expressed in “Multiple tissues”. Both databases suggested these genes were expressed in multiple tissues. But our method can find their expression level difference rather than whether they were expressed. For clearly displaying the expression level of these 23 genes across 25 tissues, we gave a box plot for each gene, which is illustrated in Supplementary Figure S1. It can be observed that for almost all genes, their expression levels on different tissues are quite different, indicating that they can be important biomarkers.

The first gene, *ARAF*, is a potential proto-oncogene that can be clustered into the *RAF* subfamily of Ser/Thr protein kinases and contributes to the regulation of cell proliferation and tissue development [38]. For its tissue-specific expression pattern, this gene has been reported to be steadily expressed in multiple tissues, including most of tissue/cell subtypes incorporated in this study, suggesting a wide expression signature [39]. More importantly, it has also been confirmed to have a unique expression pattern in the skin; thus, though this gene is expressed widely in multiple tissues, the expression levels could be different from one another [39].

Another gene named *ITGA3* has been widely reported to function as a cell surface adhesion molecule and is involved in the malignant metastasis of certain tumor subtypes [40,41]. Therefore, it is expected that this gene has low expression in the blood, as many blood cells freely float [42], suggesting that this gene may have different expression patterns across tissues corresponding to their different cell adhesion requirements. Similarly, *SLAIN2*, a microtubule dynamics-associated regulator, has also been reported to be down-regulated in blood cells compared with other tissue subtypes in our candidates, indicating the distinctive roles of *SLAIN2* as well as microtubule dynamics in different tissues [43]. *ZNF532*, a nucleic acid binding-associated gene, has been confirmed to be involved in transcriptional regulation [44,45]. Based on recent publications, this gene has also been reported to be down-regulated in liver and whole blood cells compared with other candidate tissues, implying that this gene may be a potential marker for the identification of hepatocytes and blood cells [46].

*PPIC* encodes functional peptidylprolyl isomerase C (cyclophilin C), which has been confirmed to bind to the immunosuppressant cyclosporin A, implying that this gene is an immune-associated regulator [47,48]. This gene has been confirmed to have quite high expression levels in the tibial nerve but lower levels in the cortex, suggesting its differential expression levels across various cell types and indicating potential roles in tissue classification [47]. In contrast to *PPIC*, another nerve associated-gene, *NBL1*, has been confirmed to be highly expressed in the central nervous system (brain) but lowly expressed in the peripheral nervous system (such as the spinal cord) [49,50].

*KDELR1*, a potential endoplasmic reticulum (ER)-associated gene, has no direct evidence reporting any unique expression patterns. However, this gene has been reported to affect ion transmembrane transporter activity in the nervous system and blood cells [51,52], so it is likely that this gene may have specific expression patterns corresponding to divergent ion transmembrane transporter activity requirements in different tissues.

*PLP2*, which has not been reported to display unique expression patterns, has been found to increase neuronal apoptosis when down-regulated and promotes cell proliferation in leukemia when up-regulated, indicating that different expression levels are required in tissues based on their needs for cell apoptosis and proliferation [53,54].

*STAT6*, a transcription factor in the STAT family, has been confirmed to contribute to the proliferation and differentiation of T helper 2 cells, indicating its specific role in the immune system and certain cell types [55,56,57].

*ARHGAP23* is an effective component of the Rho GTPase family and has been widely reported to be involved in transmembrane receptor signal transduction [58]. With regards to its detailed expression, this gene has been confirmed to be down-regulated in blood and muscles, revealing a specific expression pattern [59]. Similarly, *LRIG3* also has quite low expression levels in the blood [60]. Moreover, *LRIG3* displays specific expression patterns in the nervous system as it regulates the normal functioning of the inner ear, implying that this gene may also contribute to the identification of nerve tissues [61,62].

As a gene encoding a functional component of the Hippo signaling pathway involved in development, growth, repair and homeostasis, *YAP1* has low expression in mature blood cells [63,64]. This gene, as well as another three genes *MANBAL*, *PTRPA* and *CLIC1* has also been reported to be differentially expressed across different human tissues [9].

Another membrane-associated gene, *TMEM109*, has also been predicted to be distinctively expressed in different tissues. Generally, this gene has been widely reported to mediate cellular responses to DNA damage, such as ultraviolet C-induced cell death [65,66]. Not present in the bone marrow or thymus, this gene may also participate in the identification of certain cell types [66].

*MOCS2*, a gene encoding the eukaryotic molybdoenzyme, has been widely reported to contribute to molybdenum cofactor synthesis [67]. For its expression pattern, this gene has been confirmed to be predominantly expressed in heart and skeletal muscle, thus contributing to the identification of muscle and heart cells [67,68,69].

*PTPRF*, a member of the protein tyrosine phosphatase (PTP) family, has been reported to participate in various core survival-associated biological processes, such as cell growth, differentiation and mitosis [70,71], which suggests that differential expression patterns could be found in different tissues due to divergent levels of cell growth and differentiation. For example, the expression of this gene has been reported to be down-regulated in tissues with low proliferation potential, such as the heart and brain [72,73].

Encoding a common protein in muscle, *MYO1C* could be up-regulated in tissues that contain smooth muscle, skeletal muscle or heart muscle, which would distinguish these tissues from other tissues such as blood and cartilage [74,75].

The *TRIP10* gene has been confirmed to have quite a low expression level in the blood and nervous system, implying its potential in tissue classification [76,77]. The abnormal expression of this gene usually indicates specific pathological processes such as cancer and leukemia [76].

The last two genes *SERPING1* and *TOM1L2* both have been found to be expressed in all tissues [8]. *SERPING1* encodes the plasma protease C1 inhibitor involved in regulating important physiological pathways, including complement activation, blood coagulation, fibrinolysis and the generation of kinins [78], while *TOM1L2* may regulate growth factor-induced mitogenic signaling [79]. These discovered functional roles suggest potential requirements for differential expression patterns across different tissues.

We have discussed 22 of the 23 genes (*FAM127B* has little information in terms of its function) and shown their potential for tissue classification based on previous studies. Some genes, such as *ARAF*, *PTPRF*, *ITGA3* and *SLAIN2*, are involved in crucial cell processes, including cell proliferation, cell adhesion, cell invasion and the regulation of microtubule dynamics, which have diverse roles in different tissues, indicating that these genes might have differential expression patterns across different tissues. Other genes were confirmed to have specific functions in particular tissues or cell types, suggesting their specific roles in these tissues or cell types. By comparing the GO annotations between the 432 quantitatively-specific expressed genes identified in this study and the 2355 tissue-enriched genes annotated previously by Uhlen et al. [3] through the online tool DAVID [80], we found that these two sets of genes showed diverse clustering on biological processes. The former ones are enriched in translational, transcriptional and posttranscriptional regulation processes, while the latter ones are enriched in those biological processes specific to cell-types or tissues, such as spermatogenesis, muscle filament sliding, cell differentiation and so forth (see Supplementary Material S6). This finding indicates those quantitatively tissue-specific genes possibly contribute to tissue-specific features through transcriptional, translational, posttranscriptional and posttranslational regulations.

## 5. Conclusions

In summary, this study identified quantitatively tissue-specific expressed genes with discriminating power for classifying different tissue types based on their expression patterns via machine-learning algorithms. As a complement to previous studies that have identified genes enriched in a single or a few tissues, our findings of genes that are commonly but also differentially expressed in multiple tissues will provide insights into a detailed understanding of tissue development and function. In addition, we provided tissue samples represented by expression profiles of 432 optimal genes in Supplementary Material S7, in which the instructions of how to use this file to build the classification model and further make predictions were also given.

## Figures and Tables

**Table 1:** The 25 tissue samples.

Tag	Tissue	Number of Samples		Tag	Tissue	Number of Samples	
		Training Dataset	Test Dataset			Training Dataset	Test Dataset
*T*_1_	Adipose tissue	577	237	*T*_2_	Adrenal gland	145	50
*T*_3_	Blood	511	54	*T*_4_	Blood vessel	689	242
*T*_5_	Brain	1259	455	*T*_6_	Breast	214	84
*T*_7_	Colon	345	169	*T*_8_	Esophagus	686	348
*T*_9_	Heart	412	201	*T*_10_	Liver	119	58
*T*_11_	Lung	320	123	*T*_12_	Muscle	430	155
*T*_13_	Nerve	304	122	*T*_14_	Ovary	97	39
*T*_15_	Pancreas	171	82	*T*_16_	Pituitary	103	82
*T*_17_	Prostate	106	48	*T*_18_	Skin	890	342
*T*_19_	Small intestine	88	52	*T*_20_	Spleen	104	60
*T*_21_	Stomach	192	75	*T*_22_	Testis	172	91
*T*_23_	Thyroid	323	139	*T*_24_	Uterus	83	32
*T*_25_	Vagina	96	27	Total	-	8436	3367

**Table 2:** The top 23 genes selected for further investigation via a literature review.

Rank	Gene	Description	The Human Protein Atlas [8]	Expression Atlas of EMBL-EBI [37]
1	*ARAF*	A-Raf Proto-Oncogene, Serine/Threonine Kinase	Expressed in all	Multiple tissues
2	*ITGA3*	Integrin Subunit Alpha 3	Mixed	Multiple tissues
3	*SLAIN2*	SLAIN Motif Family Member 2	Expressed in all	Multiple tissues
4	*ZNF532*	Zinc Finger Protein 532	Mixed	Multiple tissues
5	*PPIC*	Peptidylprolyl Isomerase C	Mixed	Multiple tissues
6	*KDELR1*	KDEL Endoplasmic Reticulum Protein Retention Receptor 1	Expressed in all	Multiple tissues
7	*NBL1*	Neuroblastoma 1, DAN Family BMP Antagonist	Expressed in all	Multiple tissues
8	*PLP2*	Proteolipid Protein 2	Expressed in all	Multiple tissues
9	*STAT6*	Signal Transducer and Activator of Transcription 6	Expressed in all	Multiple tissues
10	*ARHGAP23*	Rho GTPase Activating Protein 23	Mixed	Multiple tissues
11	*LRIG3*	Leucine Rich Repeats And Immunoglobulin Like Domains 3	Tissue enhanced (thyroid gland)	Multiple tissues
12	*MANBAL*	Mannosidase Beta Like	Expressed in all	Multiple tissues
13	*PTPRA*	Protein Tyrosine Phosphatase, Receptor Type A	Expressed in all	Multiple tissues
14	*YAP1*	Yes Associated Protein 1	Mixed	Multiple tissues
15	*CLIC1*	Chloride Intracellular Channel 1	Expressed in all	Multiple tissues
16	*TMEM109*	Transmembrane Protein 109	Expressed in all	Multiple tissues
17	*MOCS2*	Molybdenum Cofactor Synthesis 2	Expressed in all	Multiple tissues
18	*PTPRF*	Protein Tyrosine Phosphatase, Receptor Type F	Mixed	Multiple tissues
19	*MYO1C*	Myosin IC	Expressed in all	Multiple tissues
20	*FAM127B*	Family with Sequence Similarity 127 Member B	Expressed in all	Multiple tissues
21	*TRIP10*	Thyroid Hormone Receptor Interactor 10	Expressed in all	Multiple tissues
22	*SERPING1*	Serpin Family G Member 1	Expressed in all	Multiple tissues
23	*TOM1L2*	Target of Myb1 Like 2 Membrane Trafficking Protein	Expressed in all	Multiple tissues

## Supplementary Materials

The following are available online at http://www.mdpi.com/2073-4425/9/9/449/s1, Supplementary Material S1: The mRMR feature list yielded by the mRMR method; Supplementary Material S2: The performance of SVM classification model with different features; Supplementary Material S3: Fourteen important genes that were annotated as tissue-enriched, tissue-enhanced or group-enriched; Supplementary Material S4: Tissue enriched genes retrieved from Human Protein Atlas; Supplementary Material S5: Significantly highly expressed genes in each tissue obtained by the t test between one tissue and all other tissues. Supplementary Material S6: The GO enrichment analysis on 432 quantitatively-specific expressed genes identified in this study and 2355 tissue-enriched genes annotated previously by Uhlen et al.; Supplementary Material S7: Data and instructions used to construct the optimal SVM classification model and make predictions. Supplementary Figure S1: Box plots to show the expression level of 23 top genes across different tissues.
