# Supplementary material for: A Computational Method for Classifying Different Human Tissues with Quantitatively Tissue-Specific Expressed Genes
Source: Genes (Basel). 2018 Sep 7;9(9):449. doi: 10.3390/genes9090449 (PMC6162521; doi:10.3390/genes9090449)
Supplement: Supplementary file 1 [file genes-09-00449-s001.zip › Supplementary-Figure-S1.pdf]

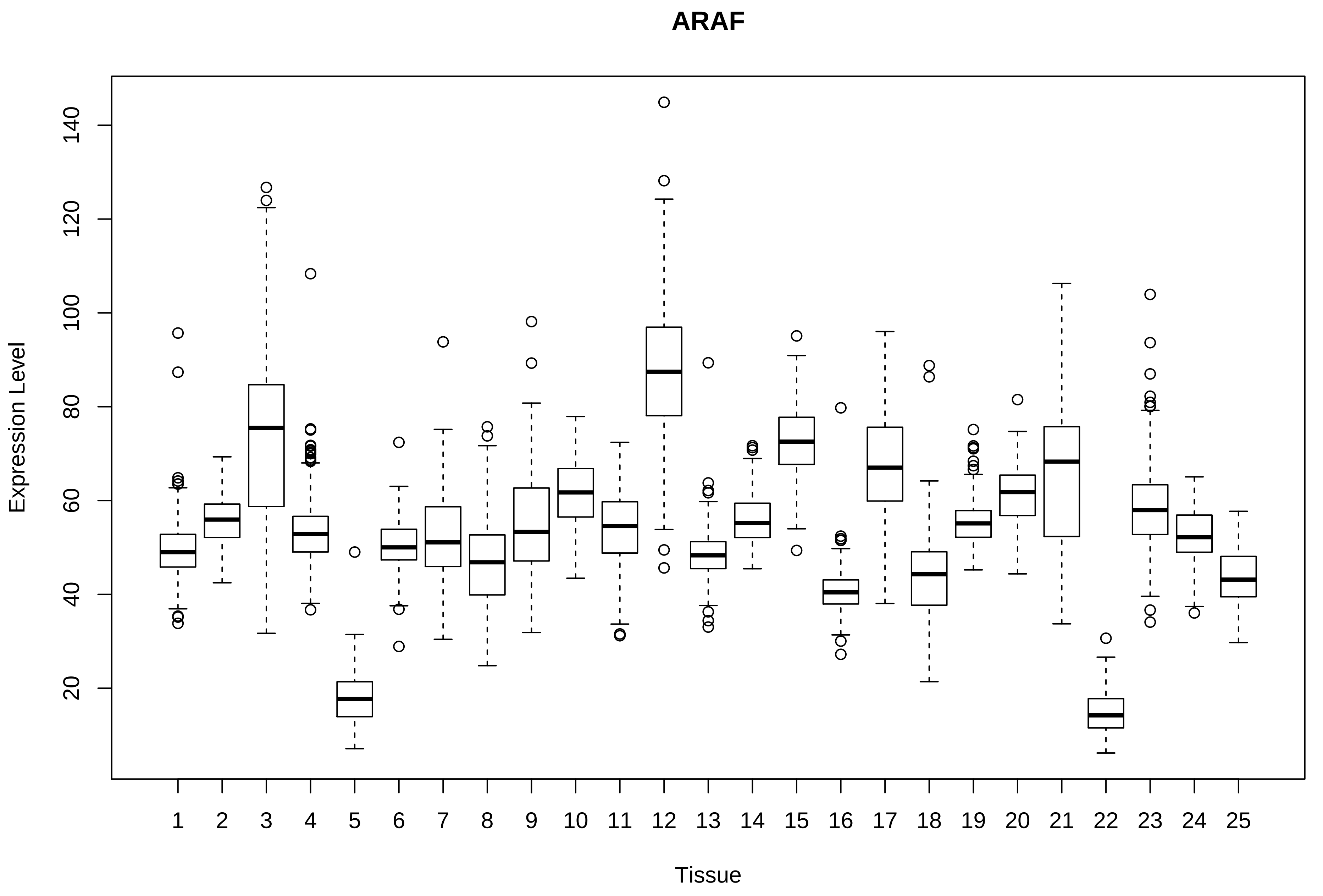
ARAF
Expression Level
20
40
60
80
100
120
140
Tissue
1
2
3
4
5
6
7
8
9
10
11
12
13
14
15
16
17
18
19
20
21
22
23
24
25

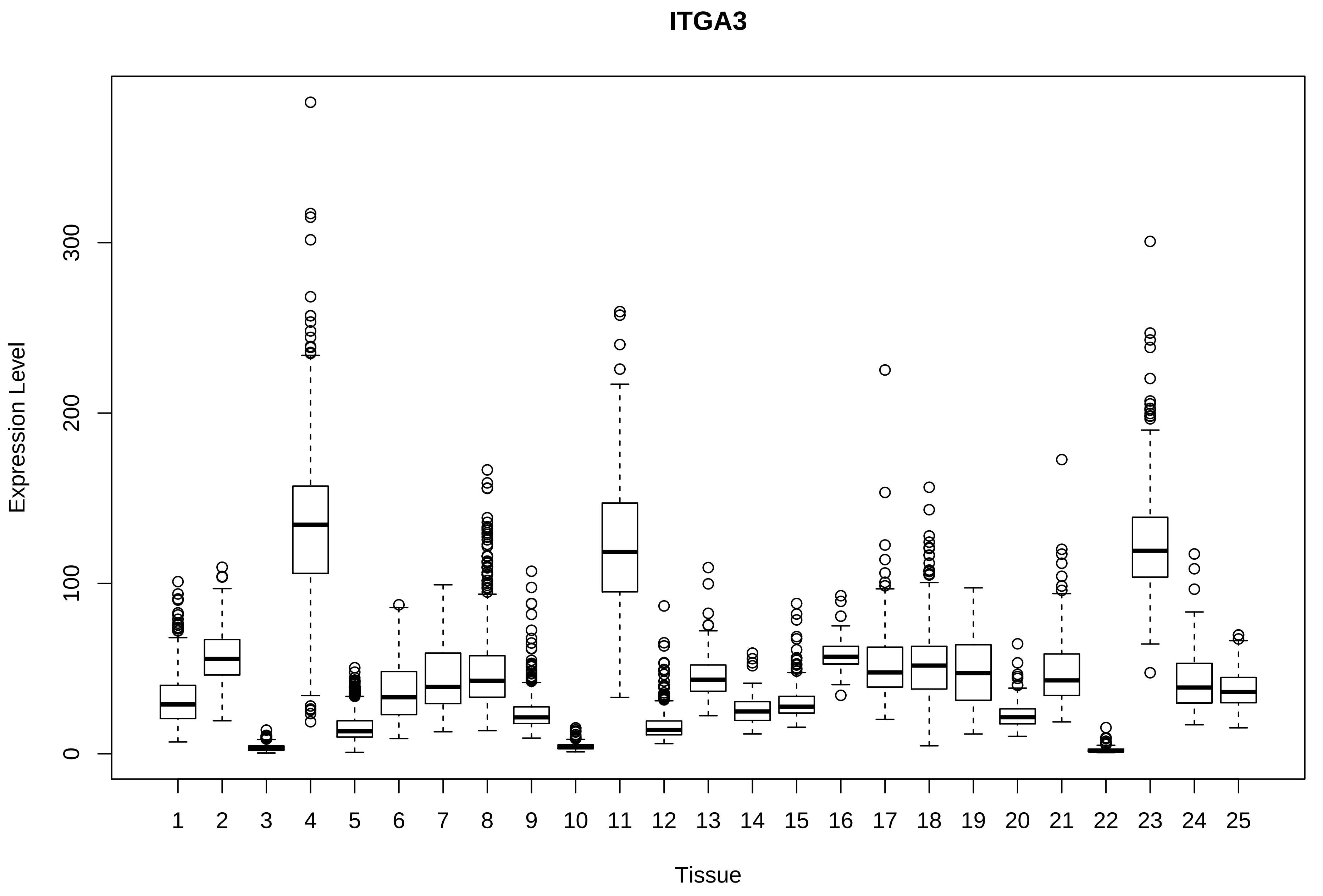
ITGA3
Expression Level
0
100
200
300
Tissue
1
2
3
4
5
6
7
8
9
10
11
12
13
14
15
16
17
18
19
20
21
22
23
24
25

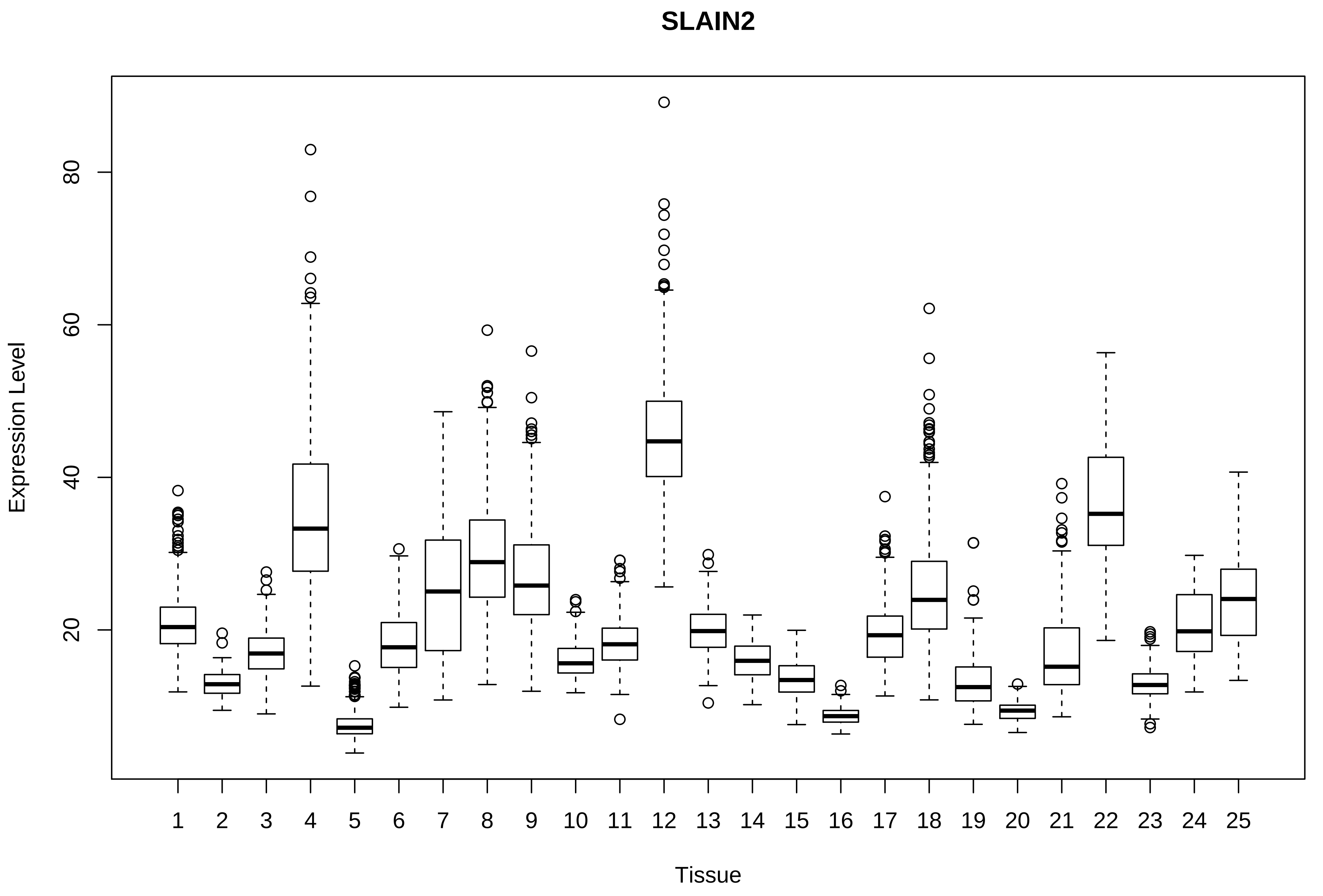
SLAIN2
Expression Level
Tissue
20
40
60
80
1 2 3 4 5 6 7 8 9 10 11 12 13 14 15 16 17 18 19 20 21 22 23 24 25

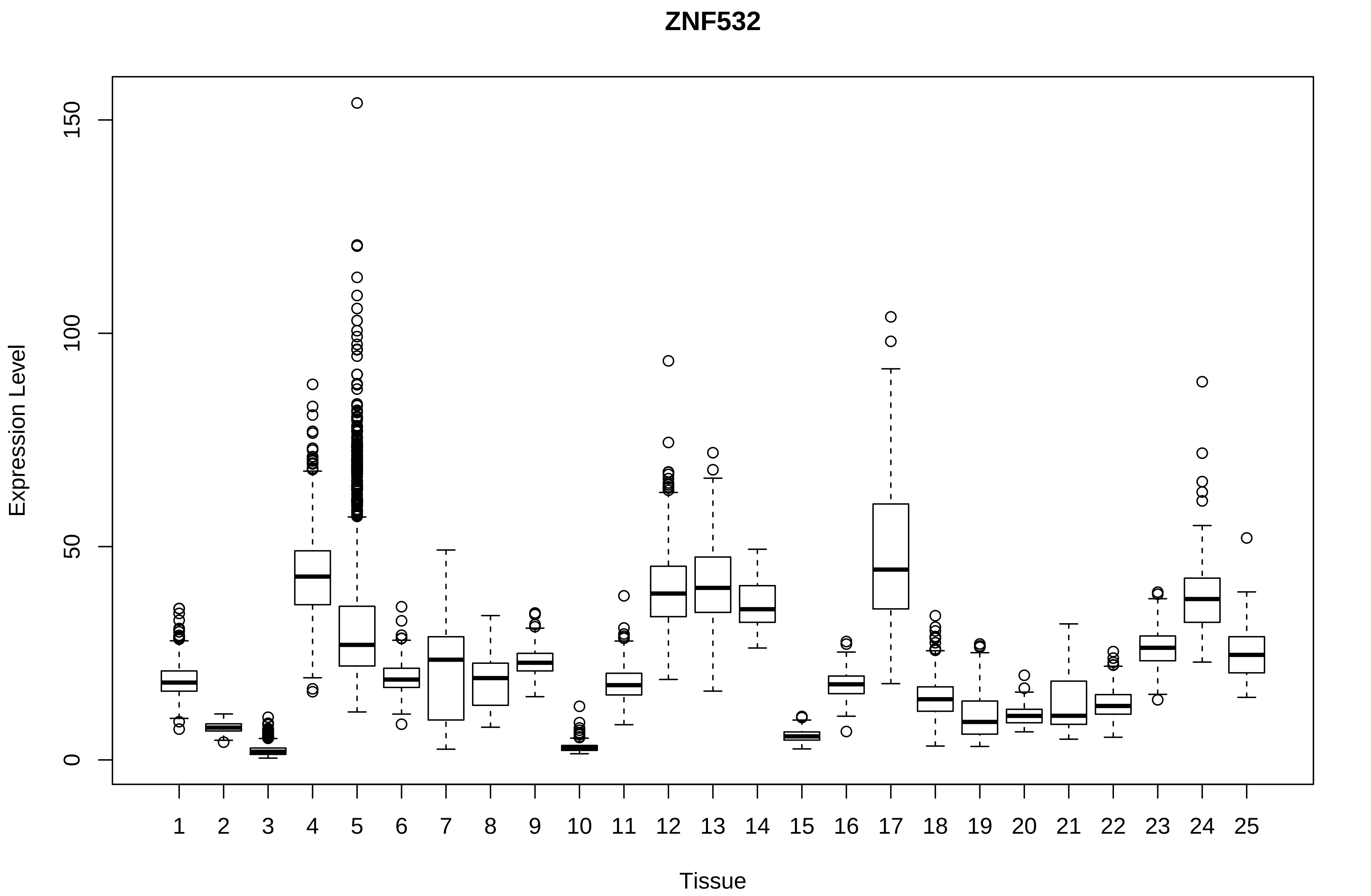
ZNF532
Expression Level
0
50
100
150
Tissue
1
2
3
4
5
6
7
8
9
10
11
12
13
14
15
16
17
18
19
20
21
22
23
24
25

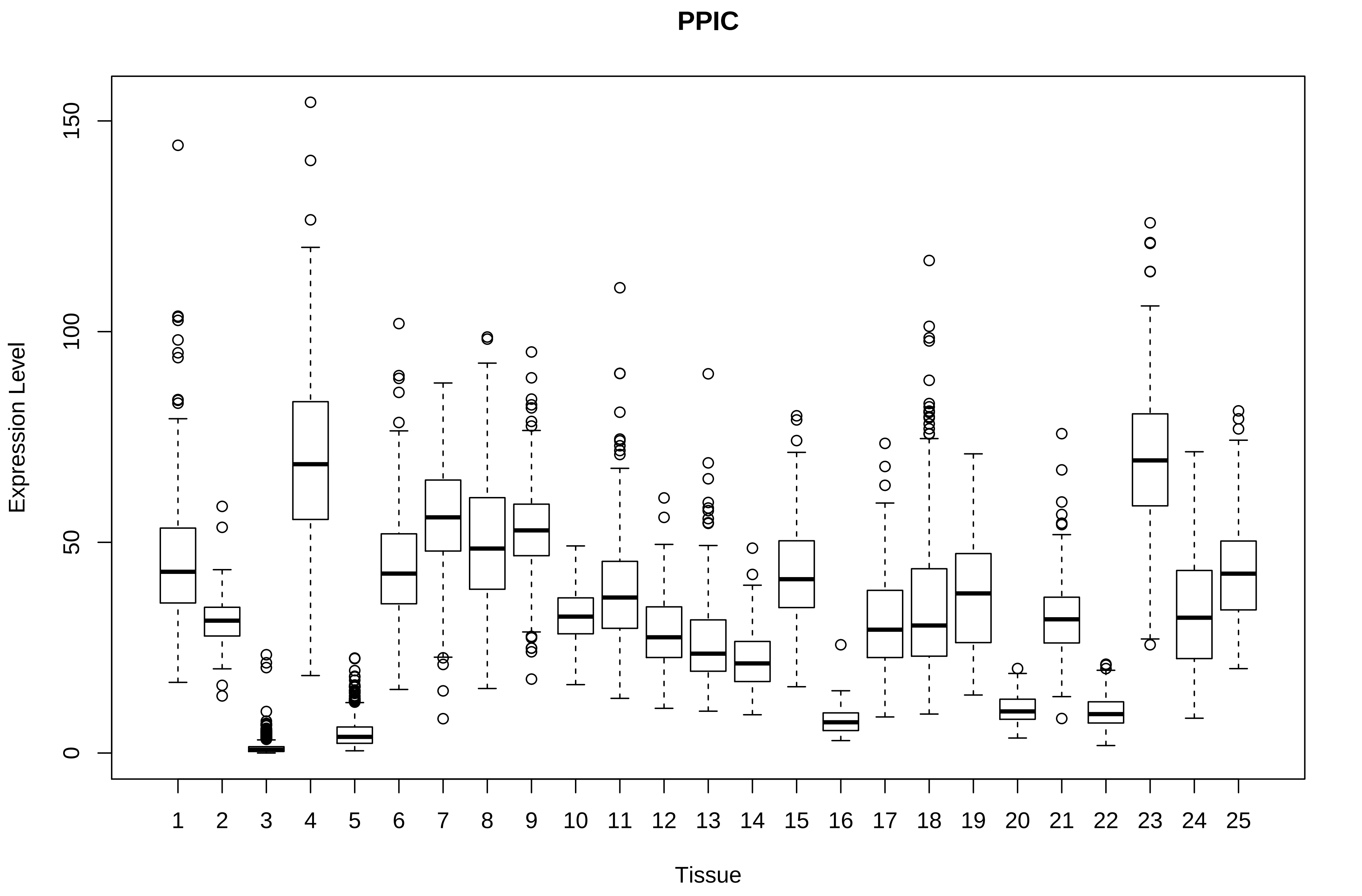
PPIC
Expression Level
0
50
100
150
1 2 3 4 5 6 7 8 9 10 11 12 13 14 15 16 17 18 19 20 21 22 23 24 25
Tissue

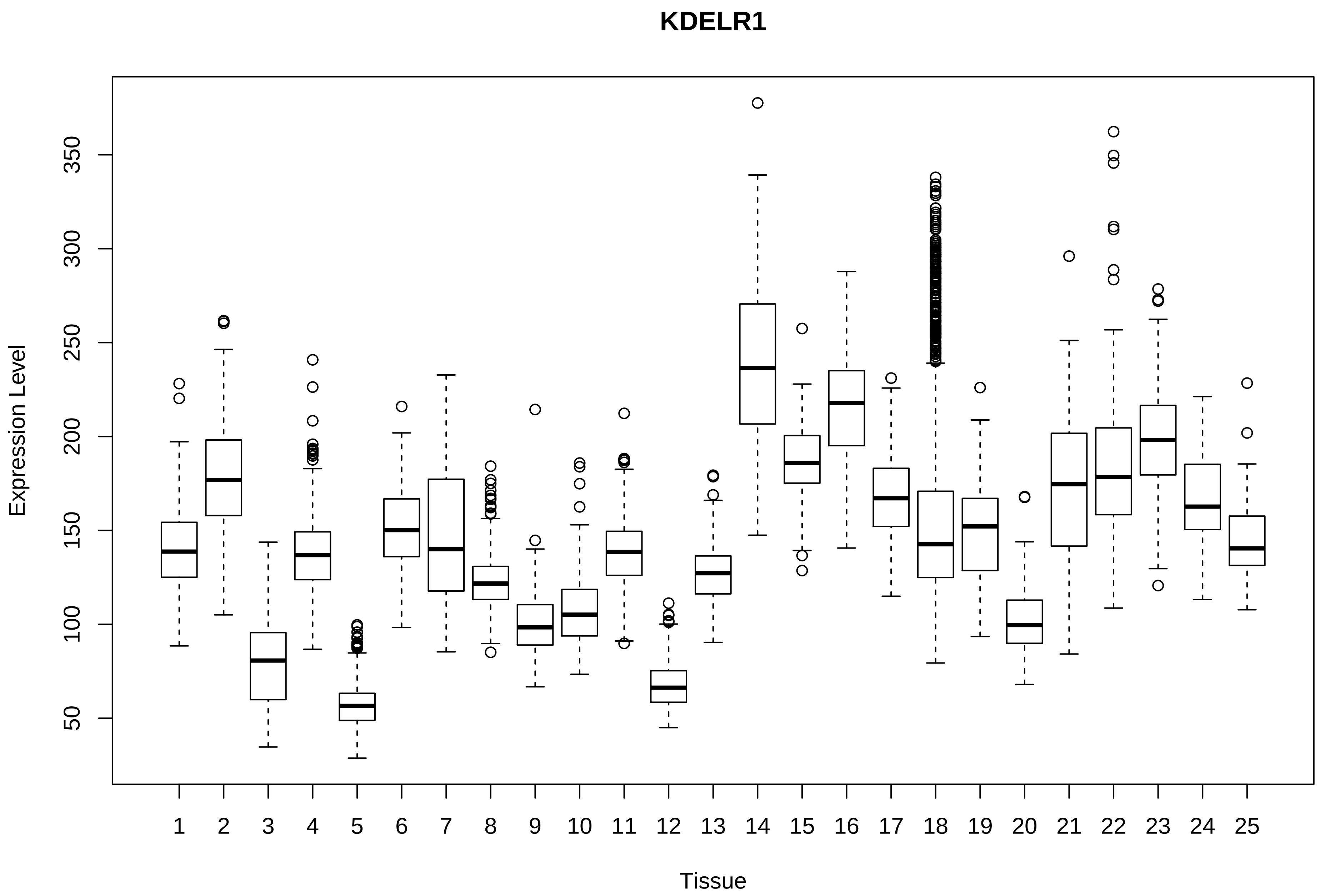

KDELR1
Expression Level
50
100
150
200
250
300
350
Tissue
1
2
3
4
5
6
7
8
9
10
11
12
13
14
15
16
17
18
19
20
21
22
23
24
25

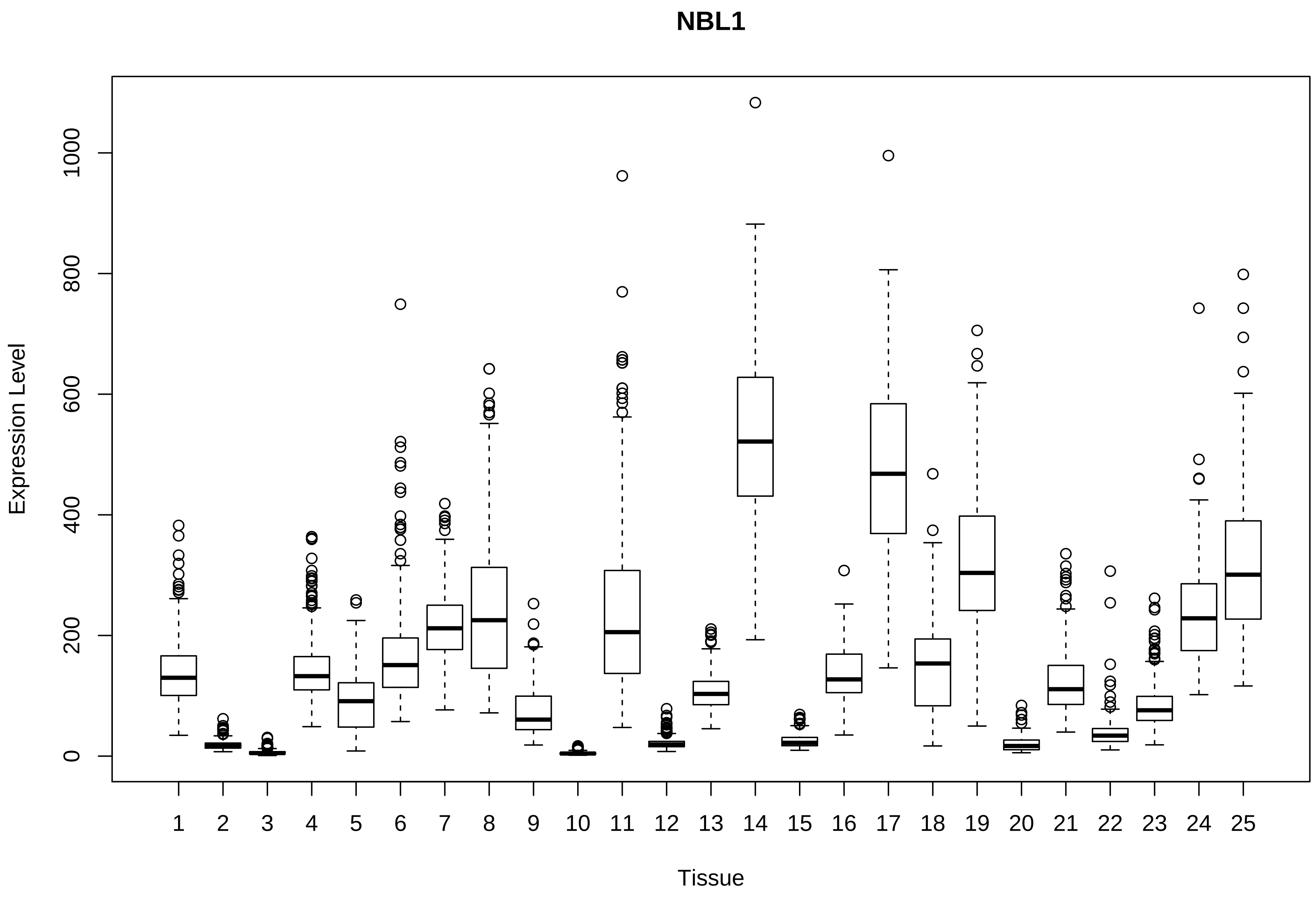

NBL1
Expression Level
0
200
400
600
800
1000
1 2 3 4 5 6 7 8 9 10 11 12 13 14 15 16 17 18 19 20 21 22 23 24 25
Tissue

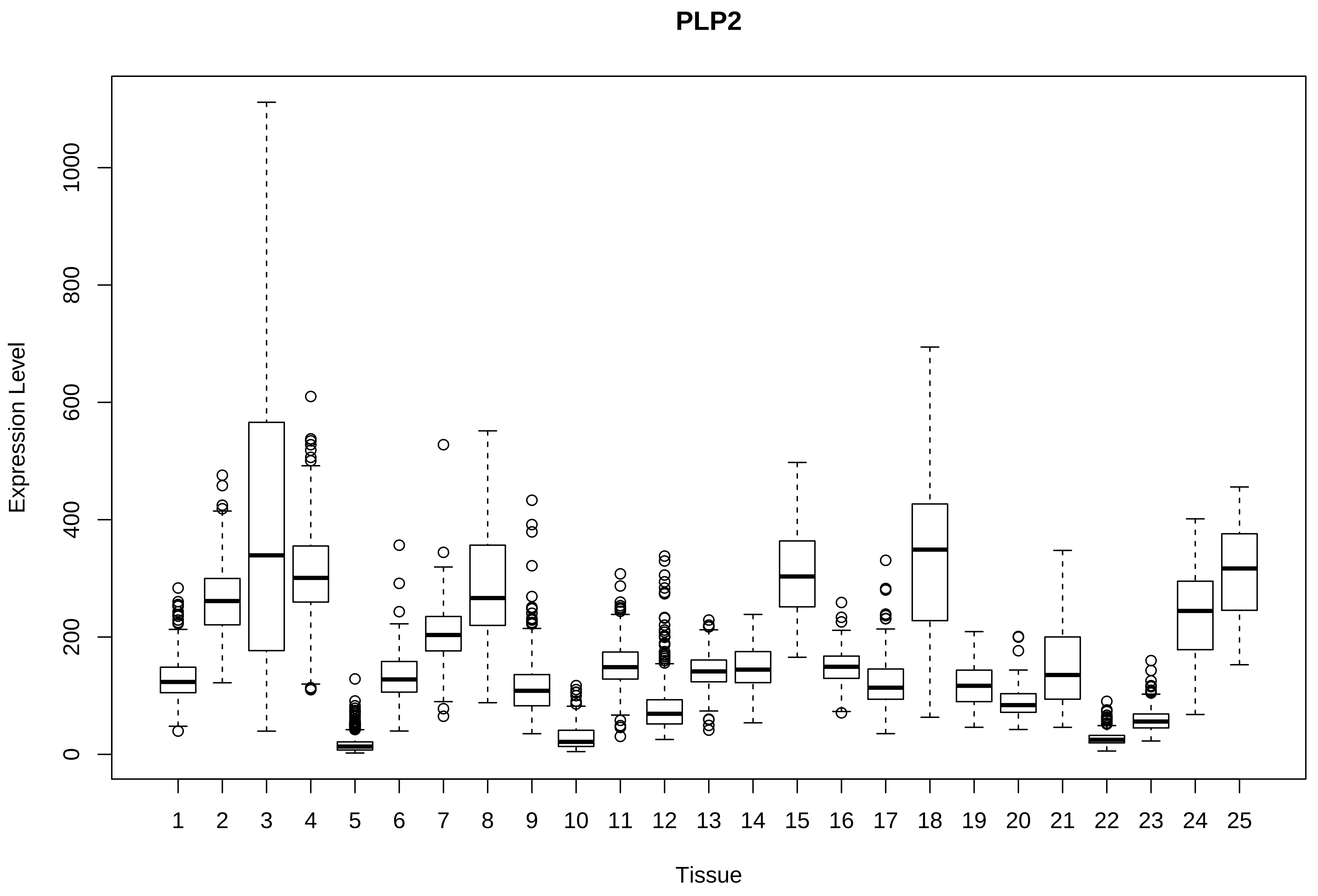
PLP2
Expression Level
0
200
400
600
800
1000
Tissue
1
2
3
4
5
6
7
8
9
10
11
12
13
14
15
16
17
18
19
20
21
22
23
24
25

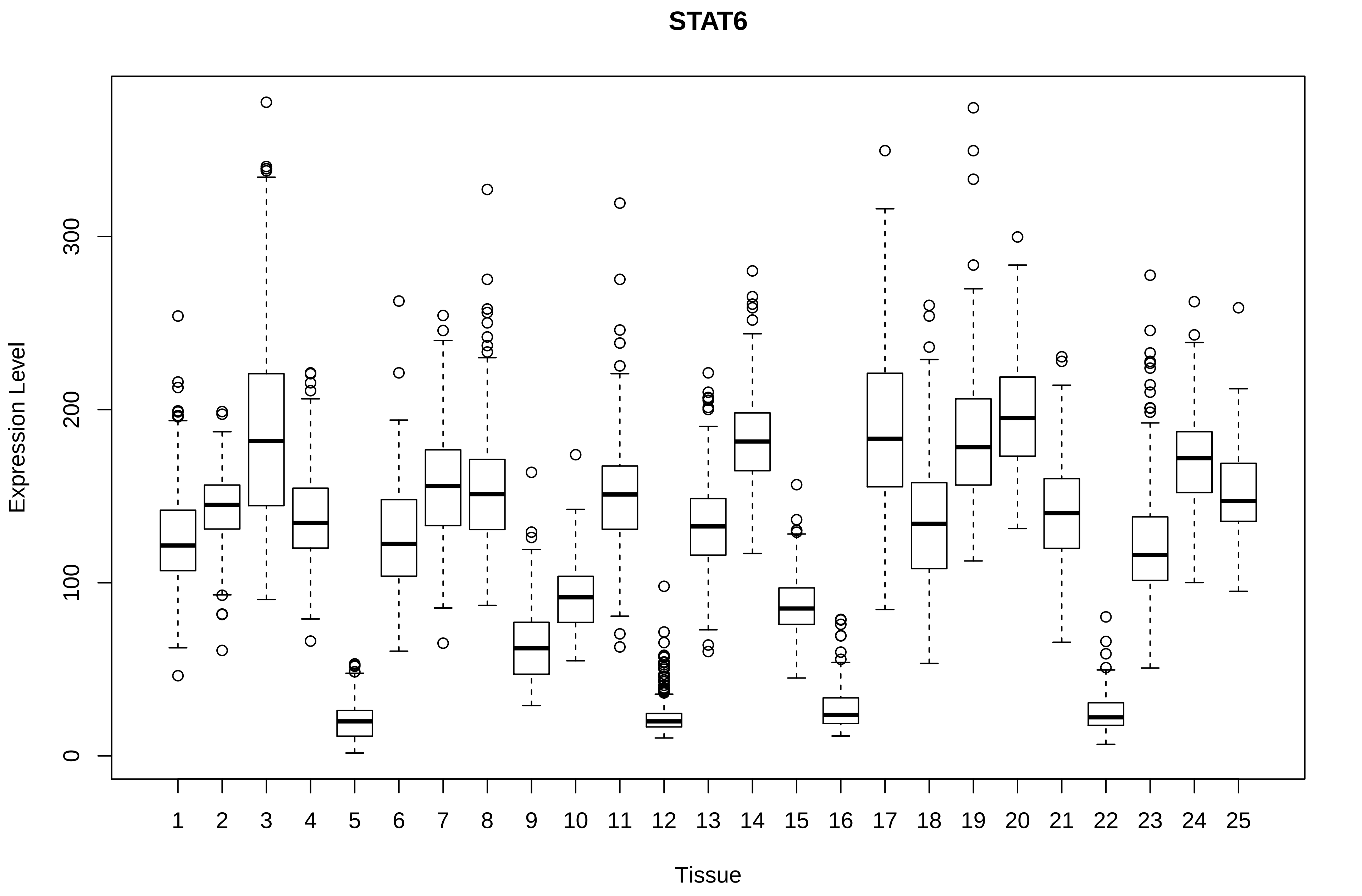
STAT6
Expression Level
0
100
200
300
Tissue
1
2
3
4
5
6
7
8
9
10
11
12
13
14
15
16
17
18
19
20
21
22
23
24
25

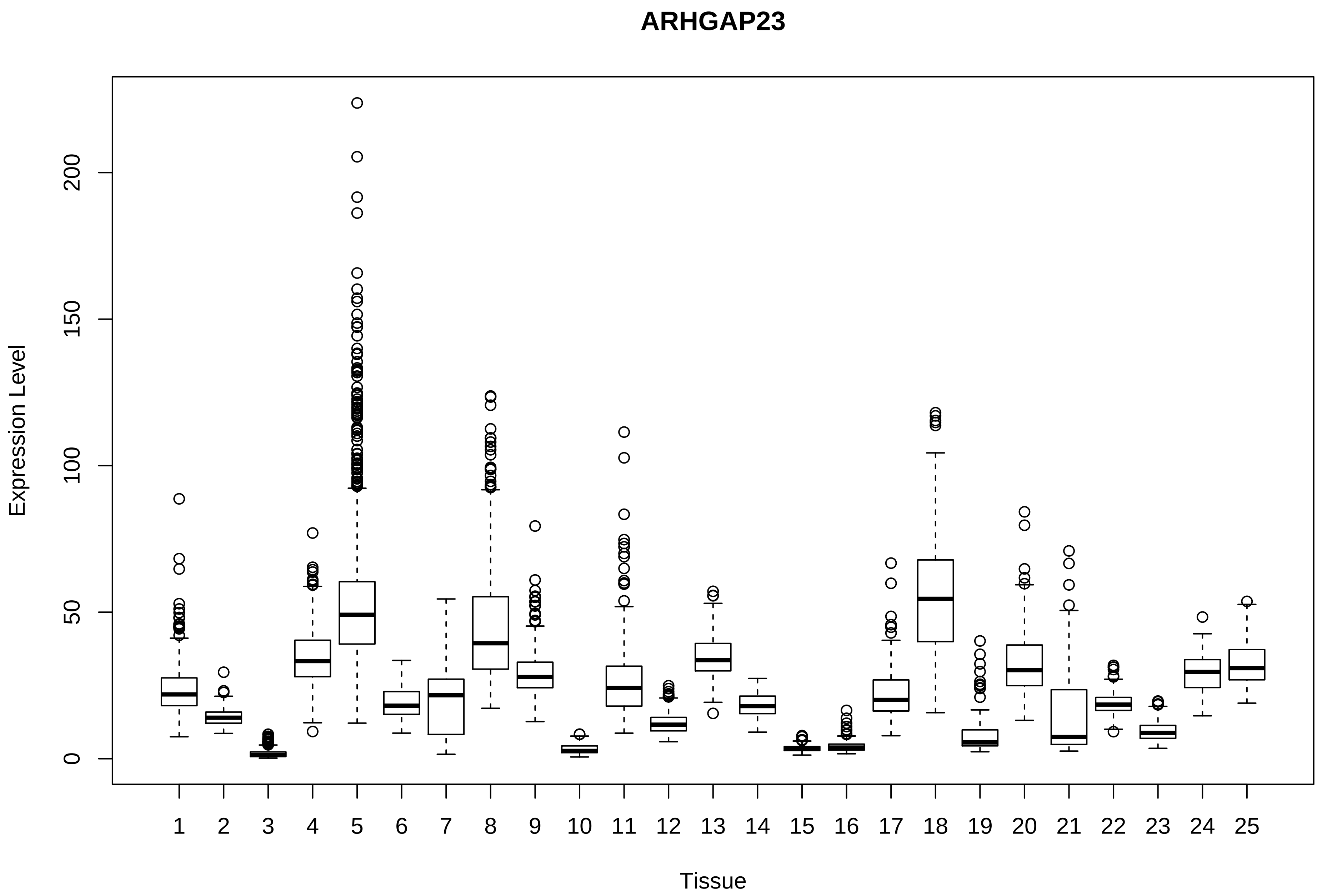
ARHGAP23
Expression Level
0
50
100
150
200
1
2
3
4
5
6
7
8
9
10
11
12
13
14
15
16
17
18
19
20
21
22
23
24
25
Tissue

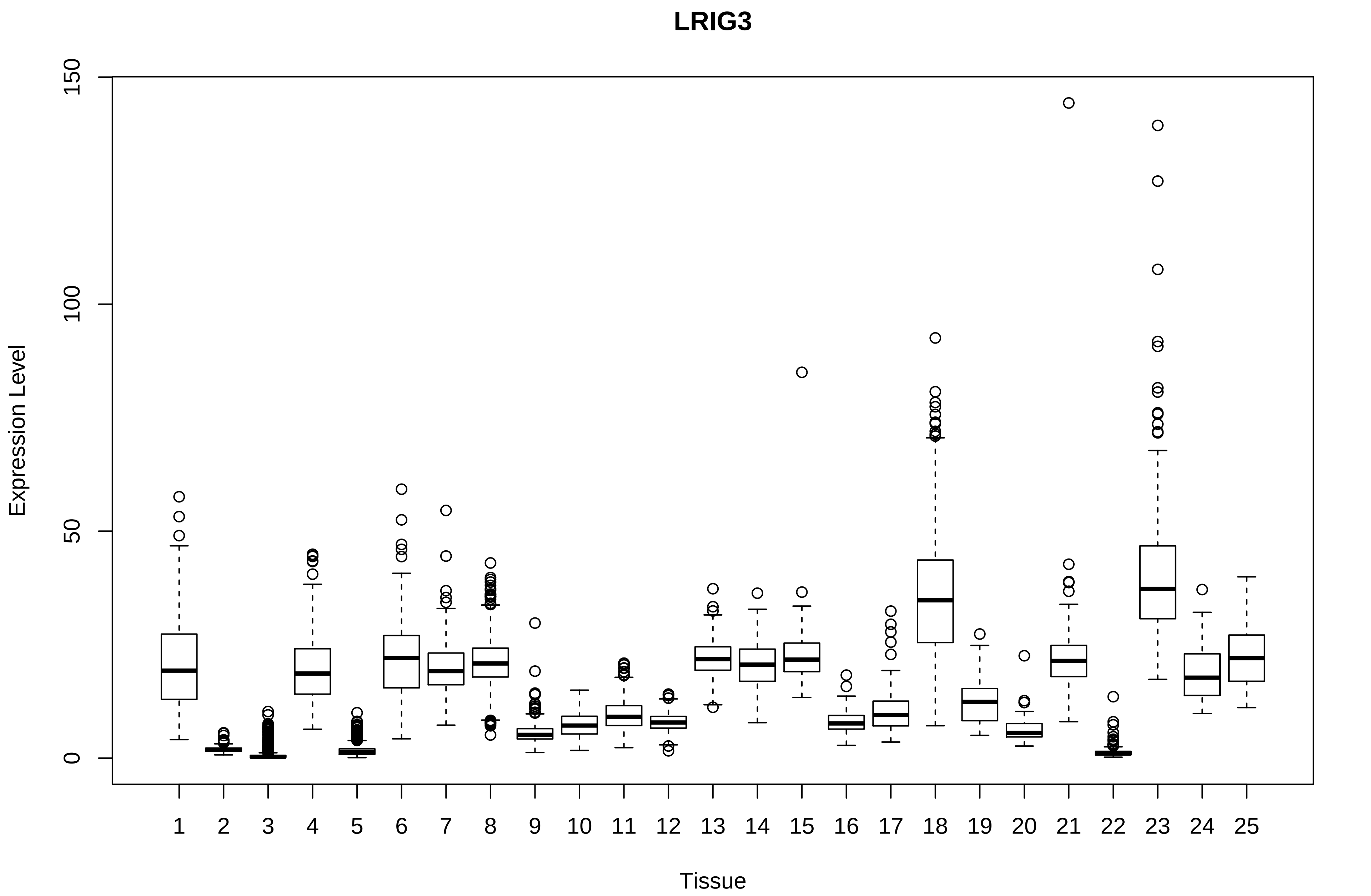
LRIG3
Expression Level
150
100
50
0
1
2
3
4
5
6
7
8
9
10
11
12
13
14
15
16
17
18
19
20
21
22
23
24
25
Tissue

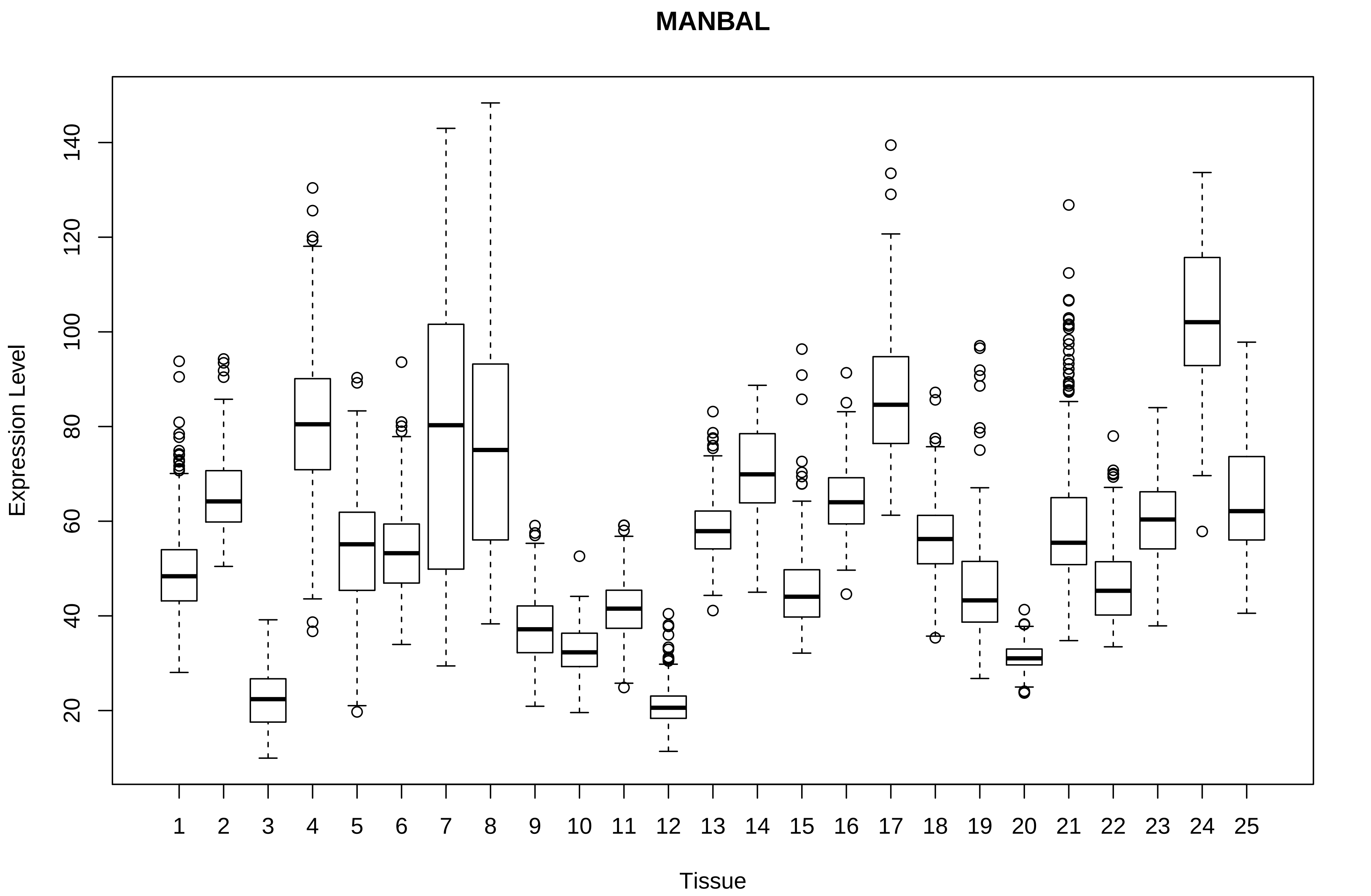
MANBAL
Expression Level
Tissue
20
40
60
80
100
120
140
1 2 3 4 5 6 7 8 9 10 11 12 13 14 15 16 17 18 19 20 21 22 23 24 25

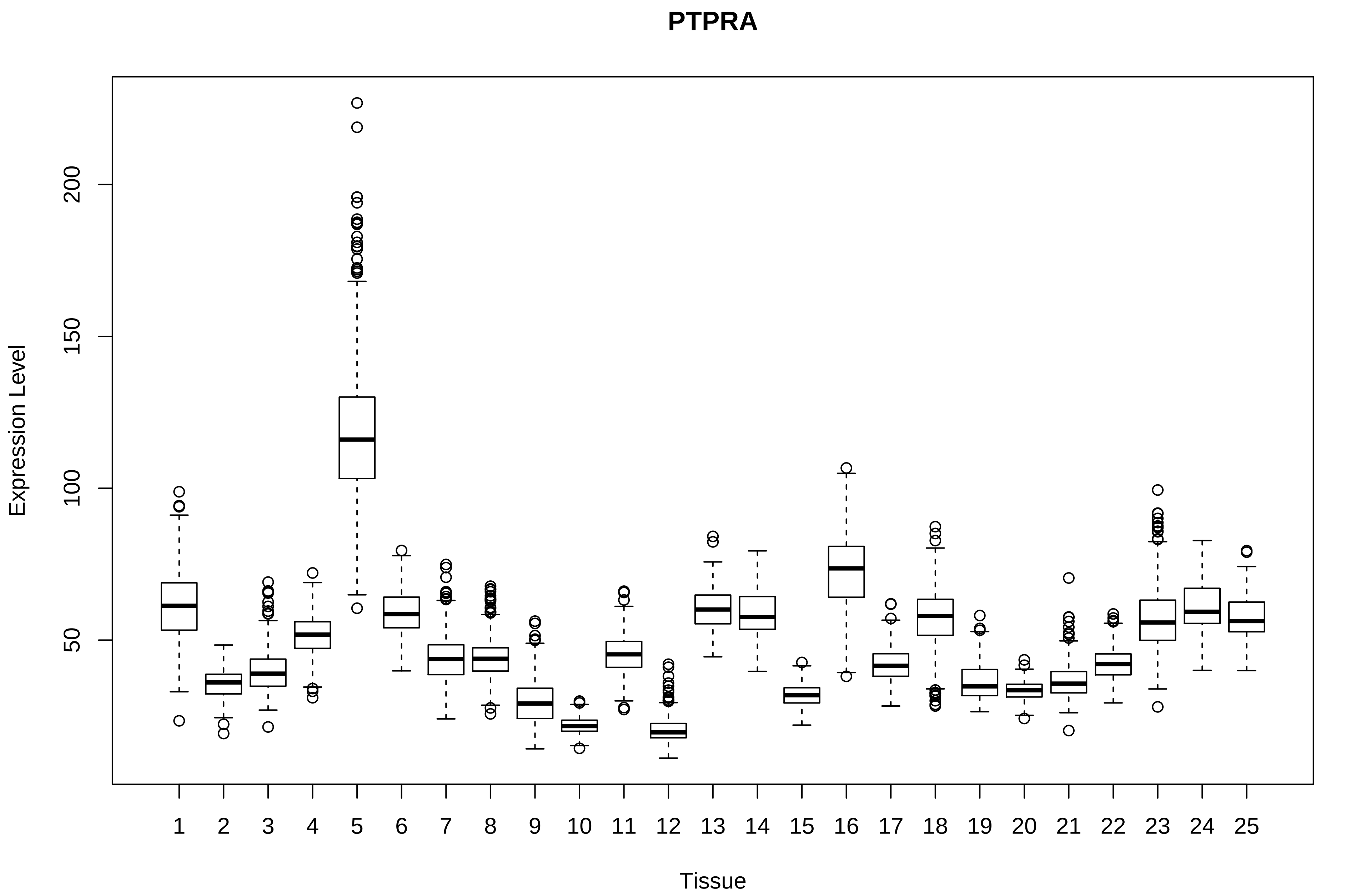
PTPRA
Expression Level
Tissue
50
100
150
200
1
2
3
4
5
6
7
8
9
10
11
12
13
14
15
16
17
18
19
20
21
22
23
24
25

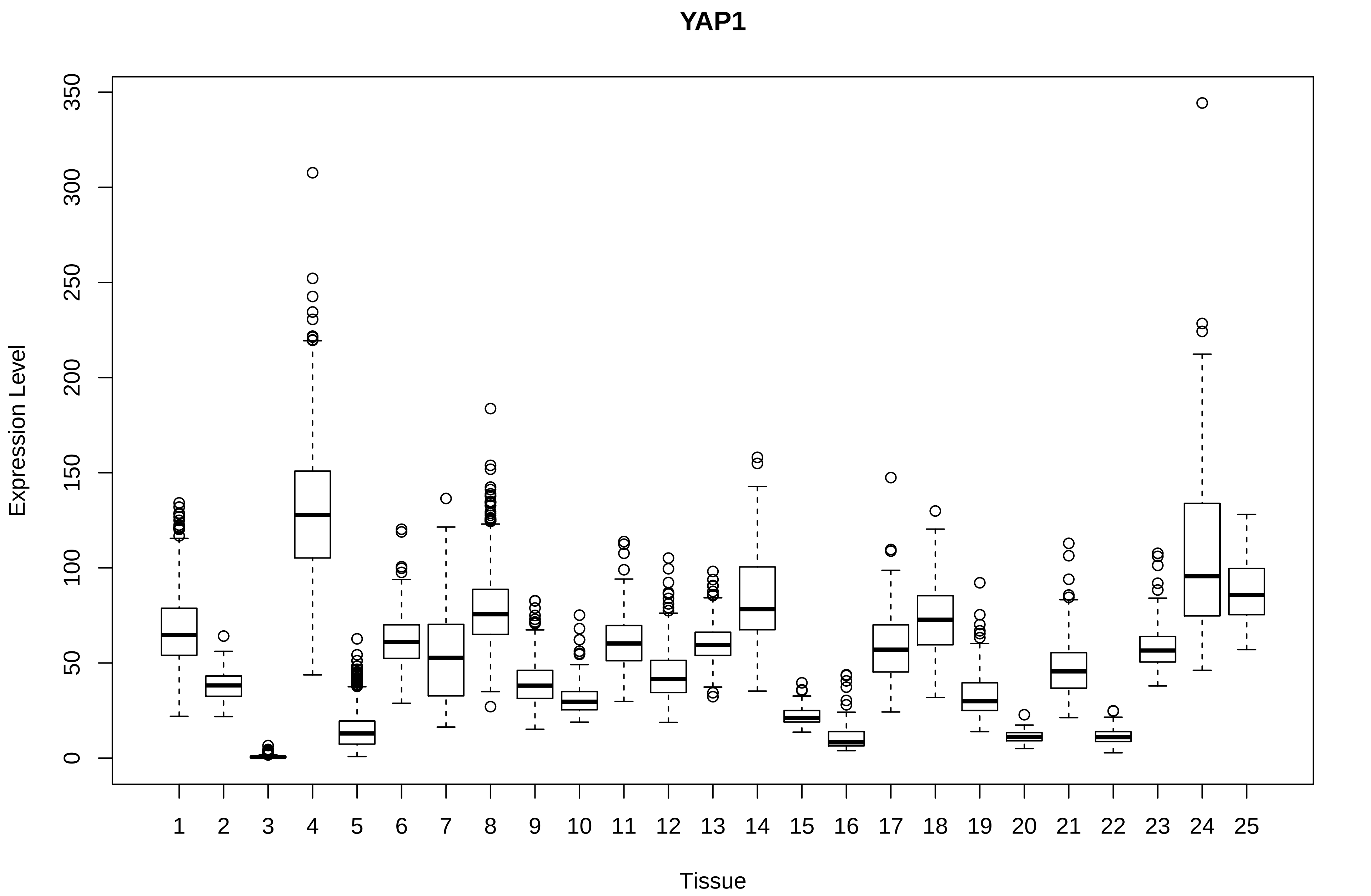
YAP1
Expression Level
0
50
100
150
200
250
300
350
Tissue
1
2
3
4
5
6
7
8
9
10
11
12
13
14
15
16
17
18
19
20
21
22
23
24
25

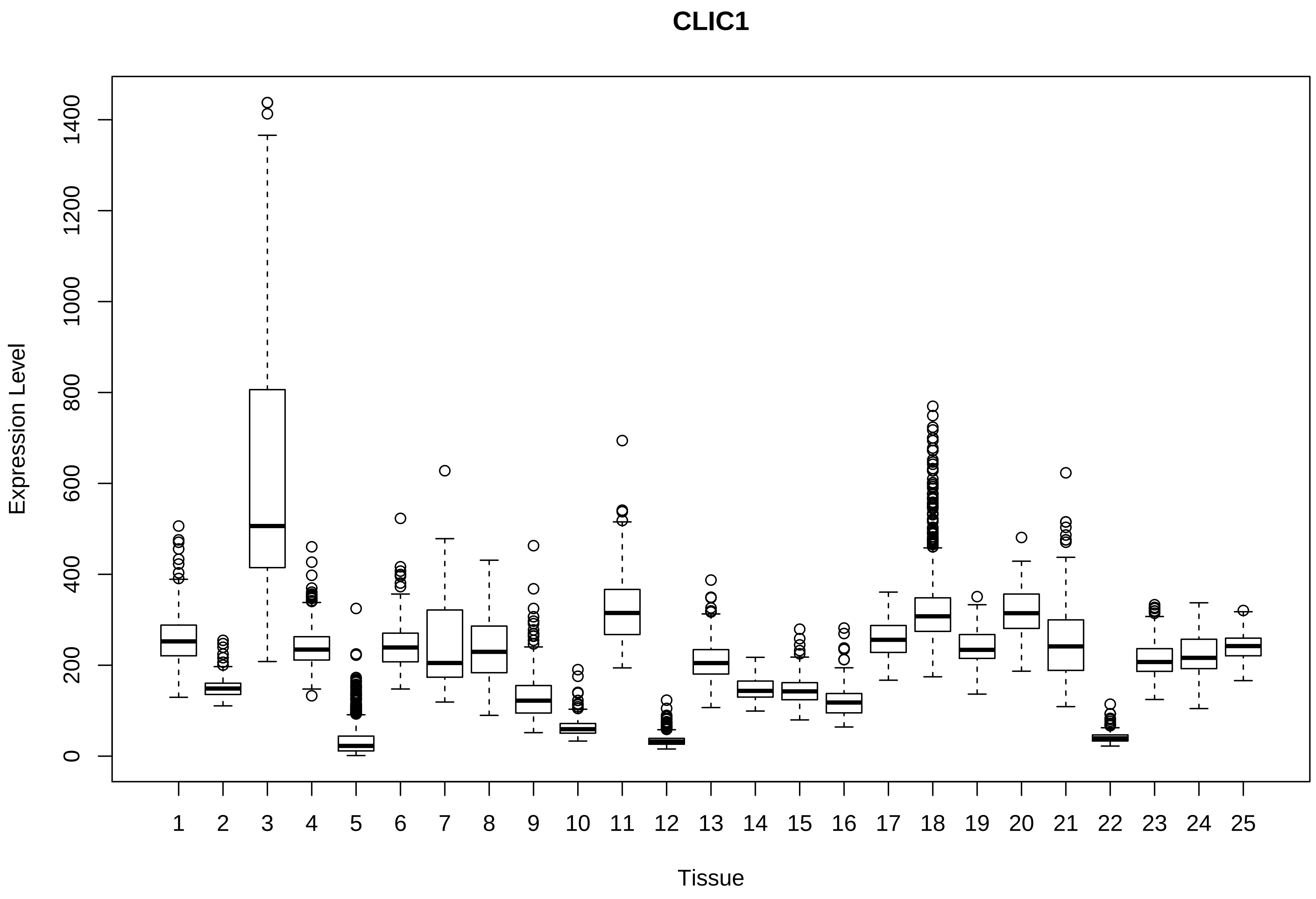
CLIC1
Expression Level
0
200
400
600
800
1000
1200
1400
Tissue
1
2
3
4
5
6
7
8
9
10
11
12
13
14
15
16
17
18
19
20
21
22
23
24
25

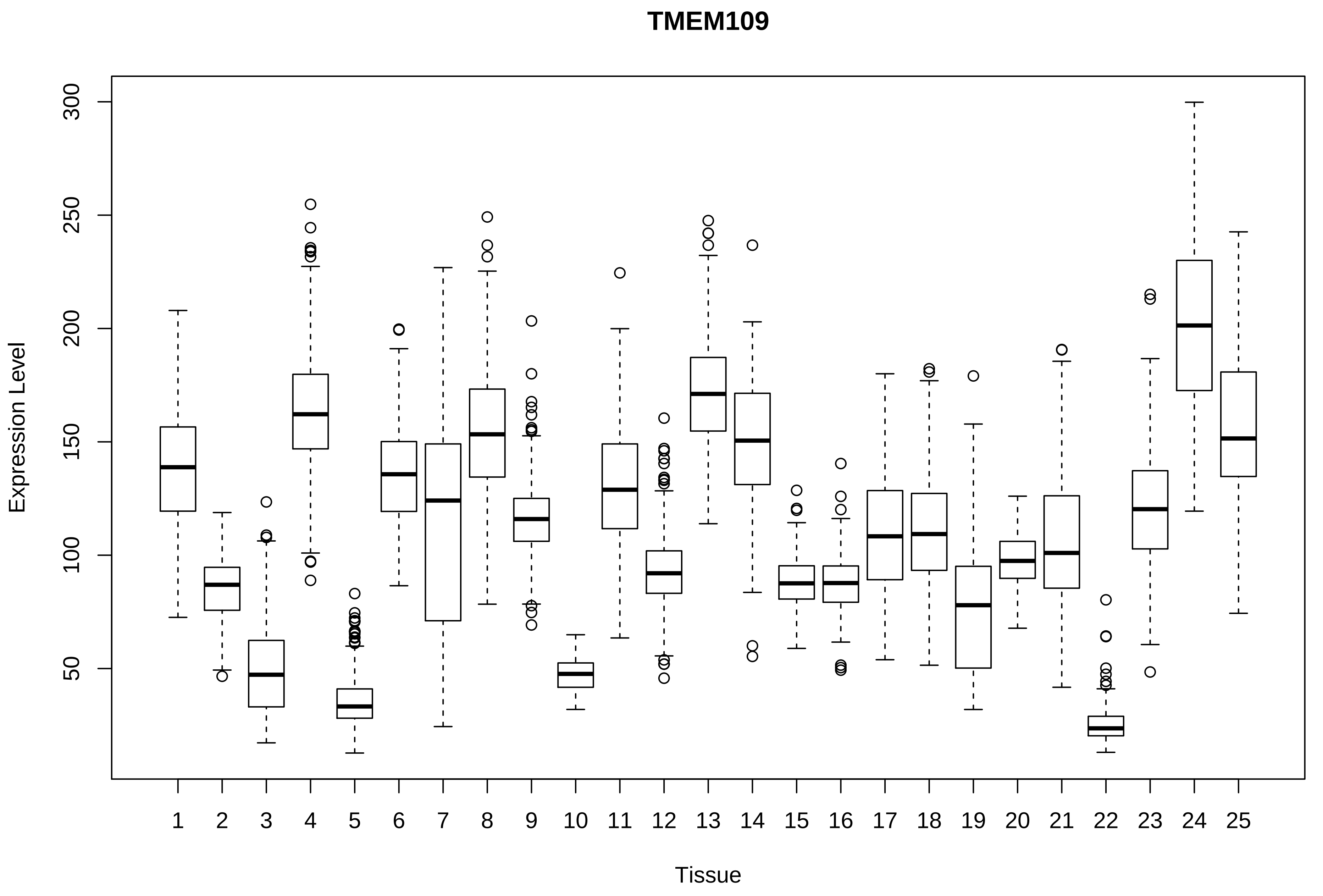
TMEM109
Expression Level
300
250
200
150
100
50
Tissue
1
2
3
4
5
6
7
8
9
10
11
12
13
14
15
16
17
18
19
20
21
22
23
24
25

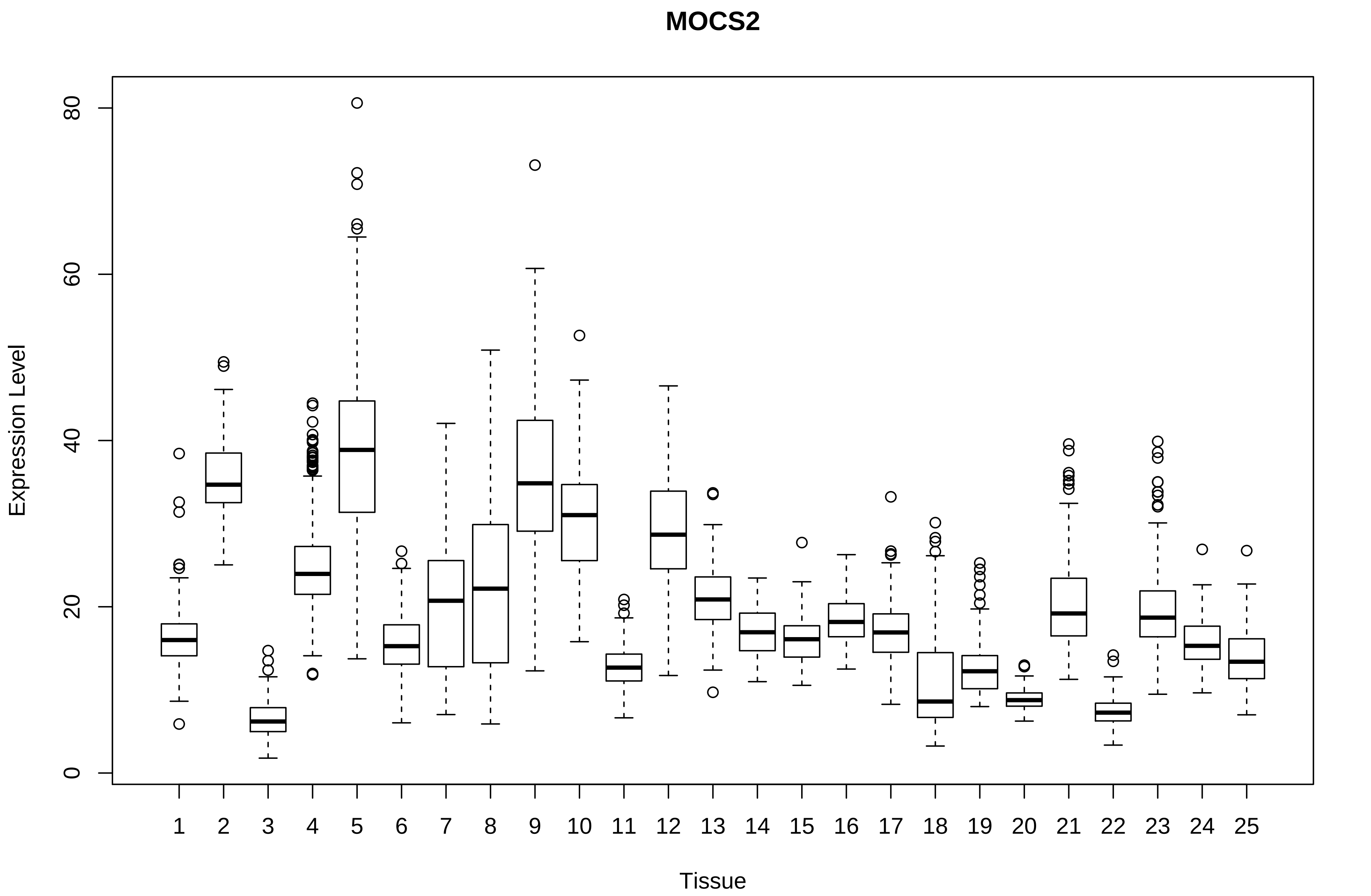

MOCS2
Expression Level
0
20
40
60
80
Tissue
1
2
3
4
5
6
7
8
9
10
11
12
13
14
15
16
17
18
19
20
21
22
23
24
25

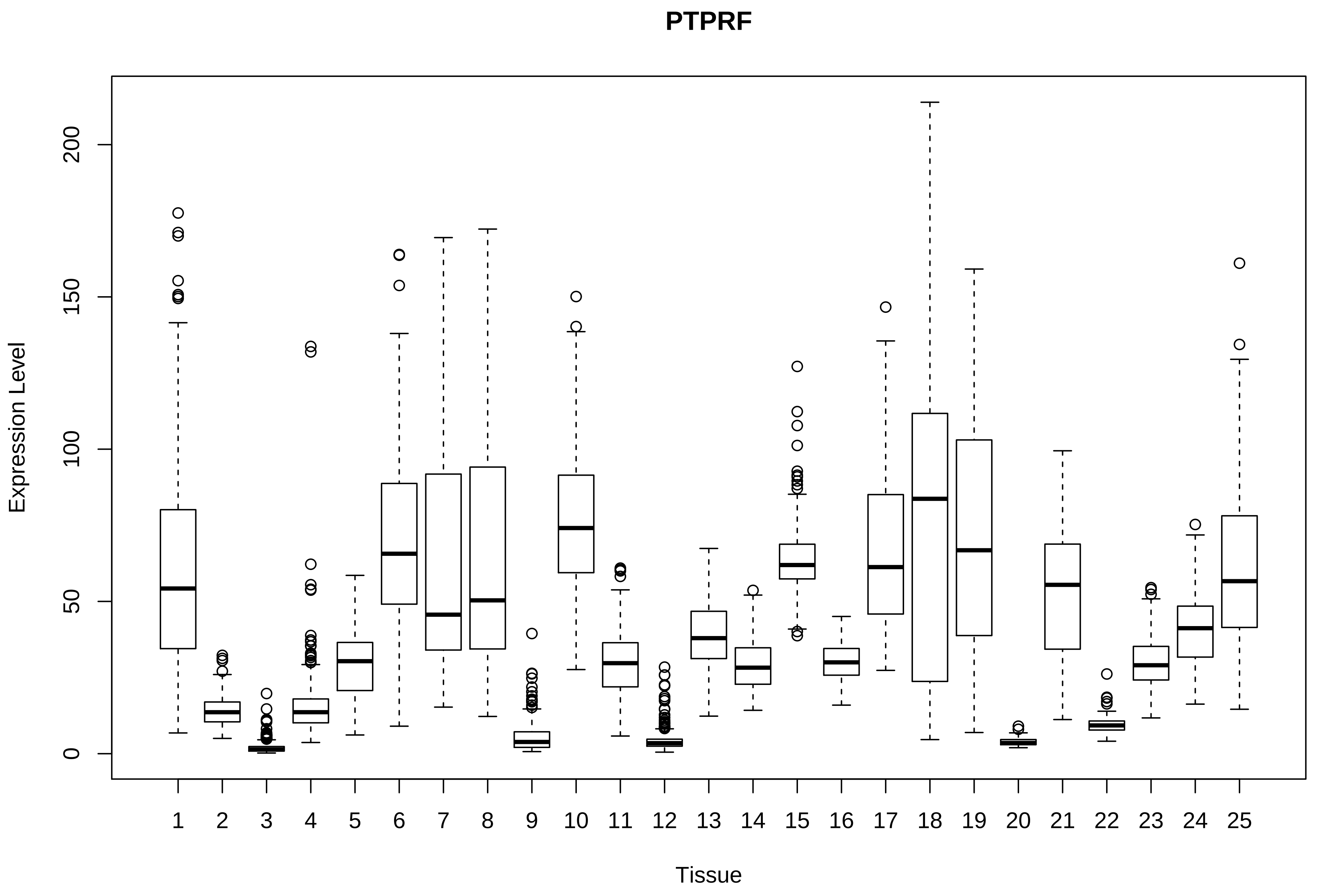
PTPRF
Expression Level
0
50
100
150
200
1
2
3
4
5
6
7
8
9
10
11
12
13
14
15
16
17
18
19
20
21
22
23
24
25
Tissue

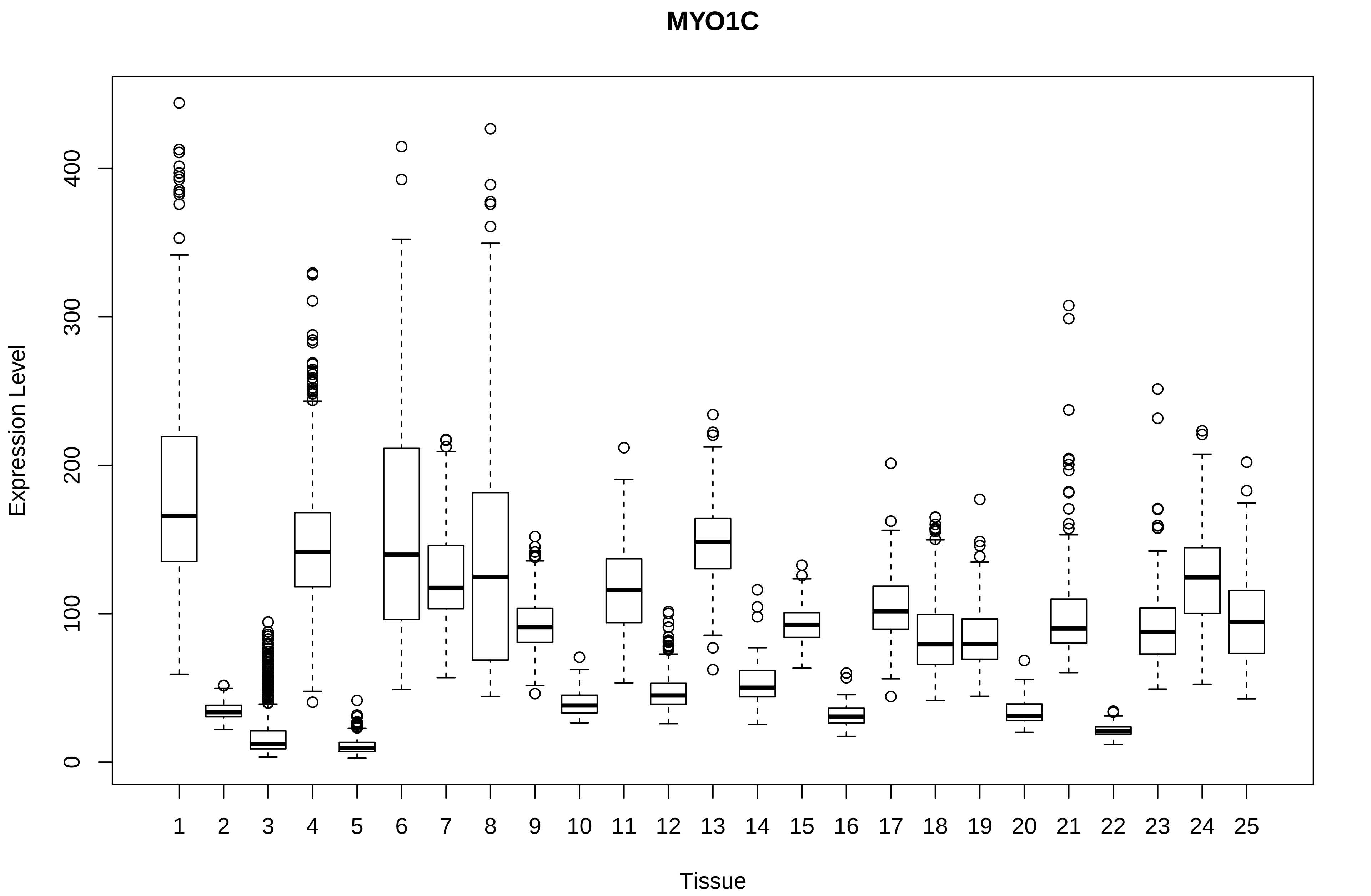
MYO1C
Expression Level
0
100
200
300
400
Tissue
1
2
3
4
5
6
7
8
9
10
11
12
13
14
15
16
17
18
19
20
21
22
23
24
25

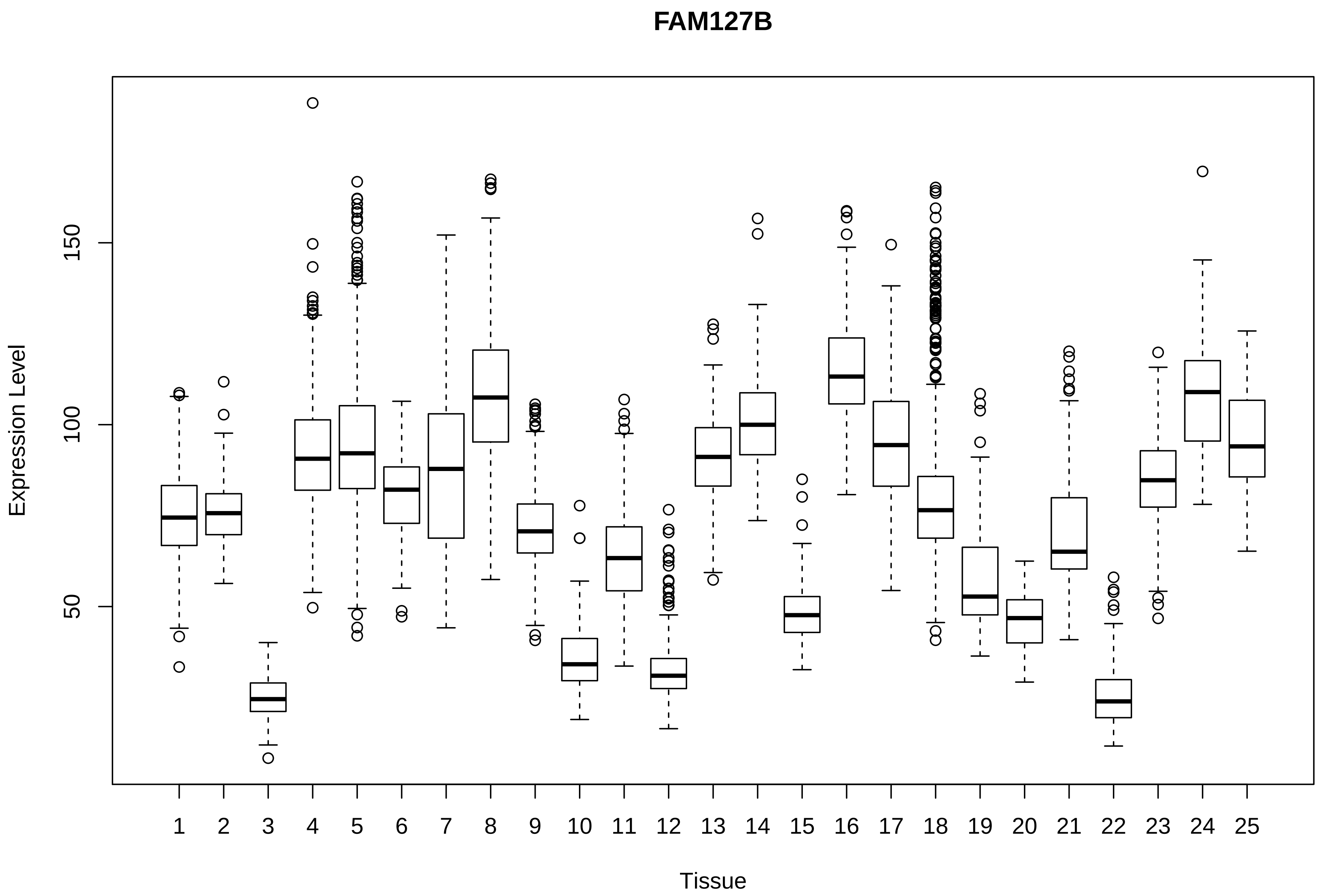

FAM127B
Expression Level
50
100
150
Tissue
1 2 3 4 5 6 7 8 9 10 11 12 13 14 15 16 17 18 19 20 21 22 23 24 25

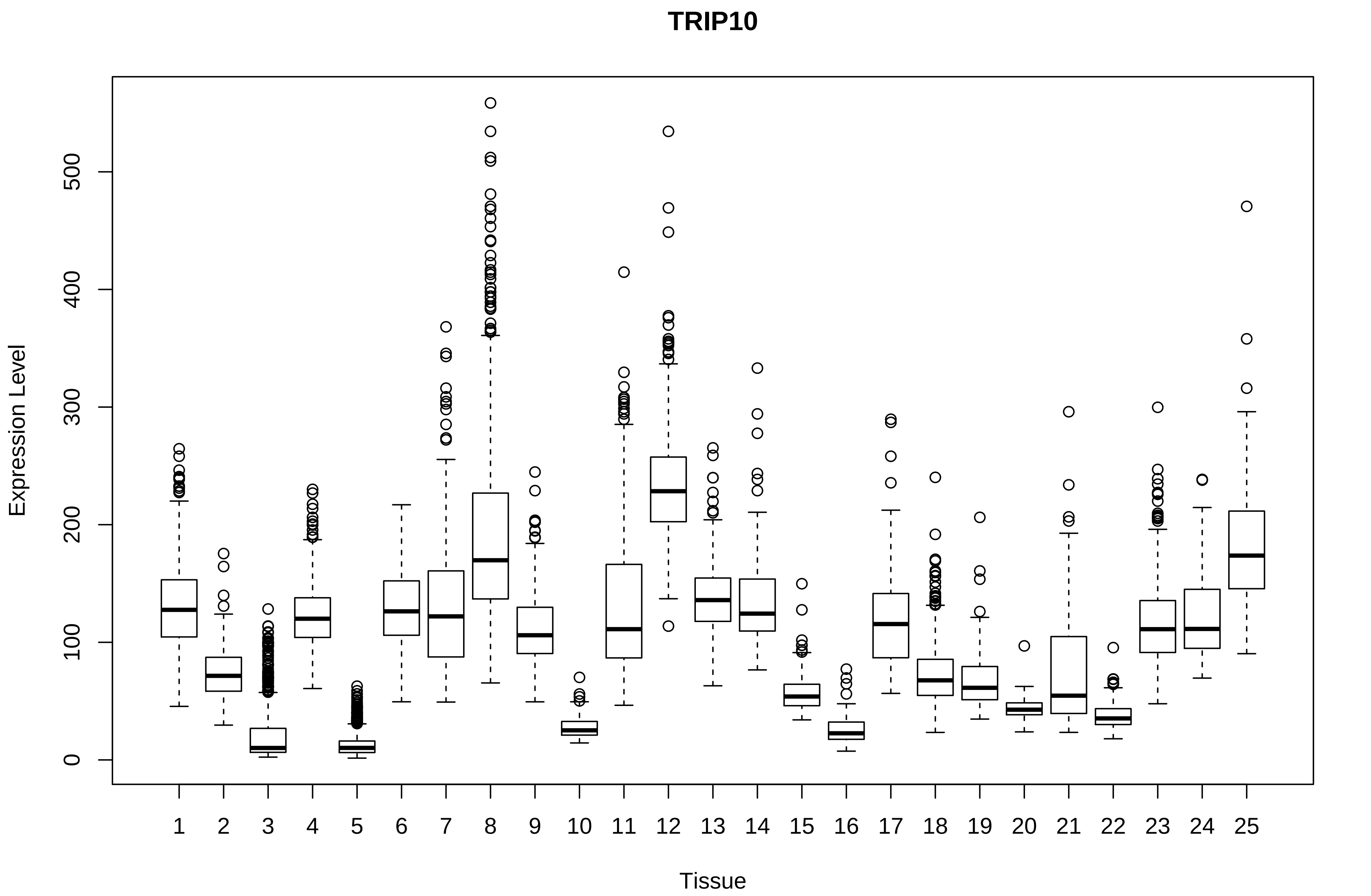

TRIP10
Expression Level
0
100
200
300
400
500
Tissue
1
2
3
4
5
6
7
8
9
10
11
12
13
14
15
16
17
18
19
20
21
22
23
24
25

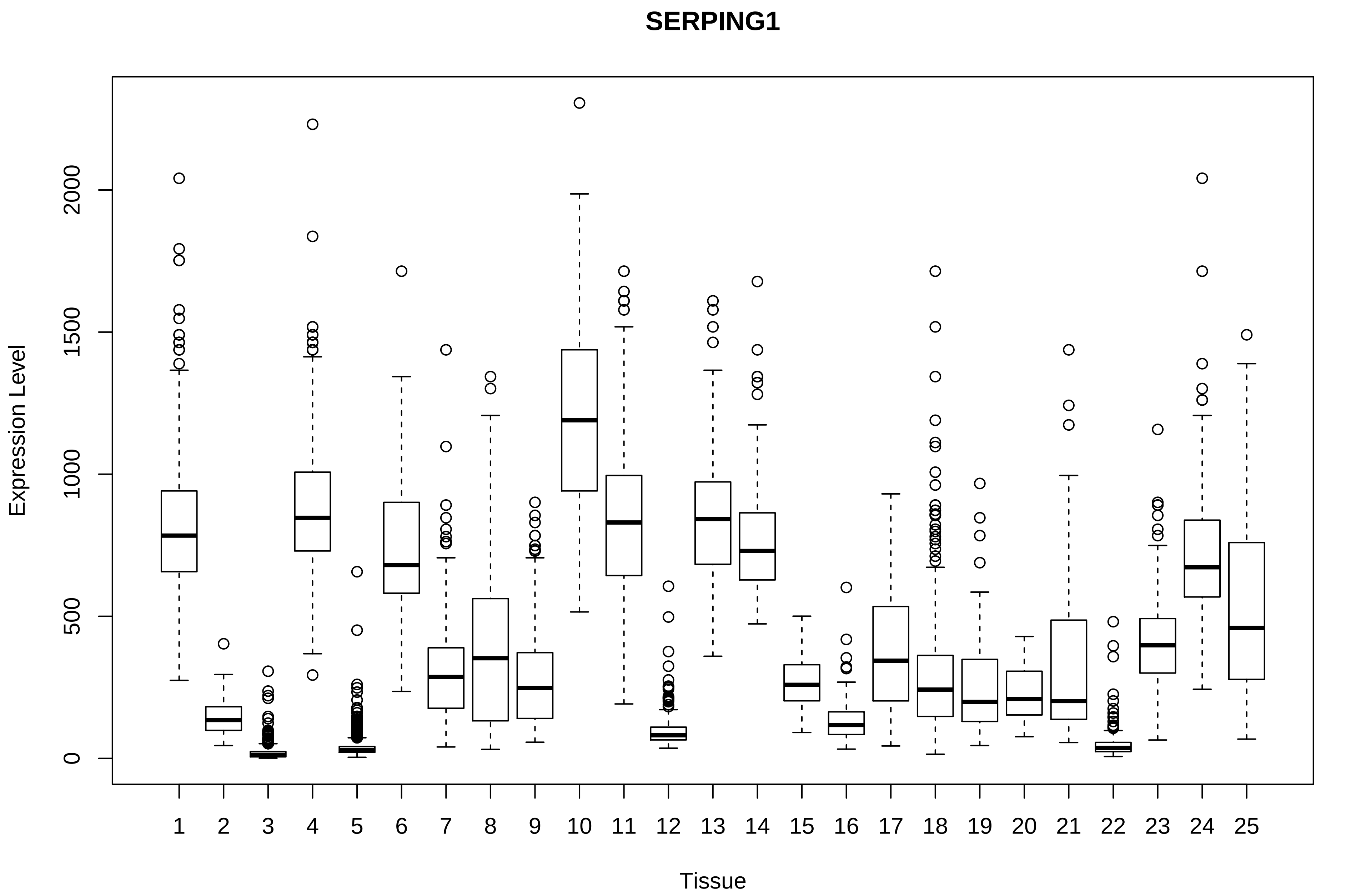
SERPING1
Expression Level
0
500
1000
1500
2000
Tissue
1 2 3 4 5 6 7 8 9 10 11 12 13 14 15 16 17 18 19 20 21 22 23 24 25

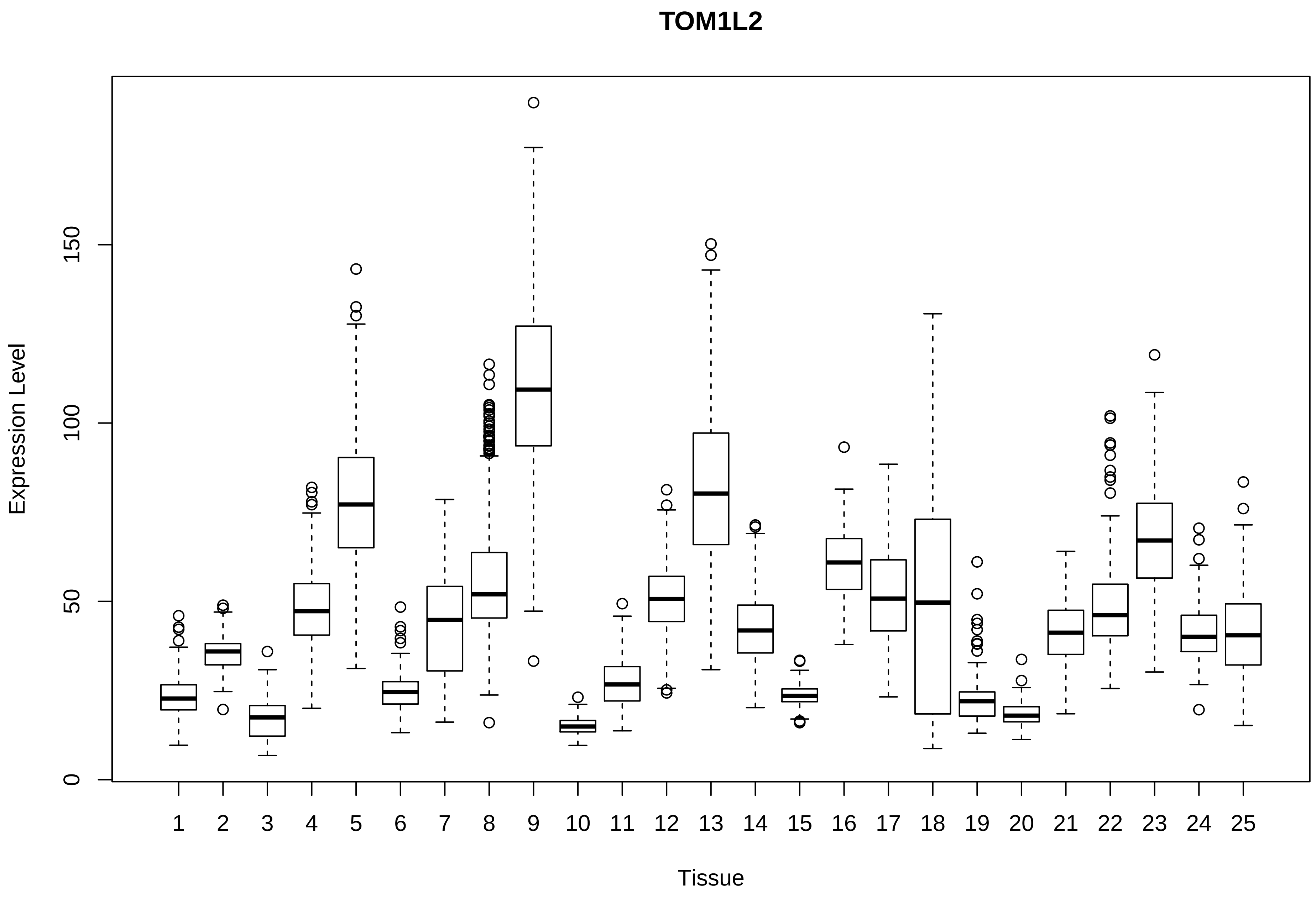
TOM1L2
Expression Level
0
50
100
150
Tissue
1
2
3
4
5
6
7
8
9
10
11
12
13
14
15
16
17
18
19
20
21
22
23
24
25
