# Supplementary material for: A Computational Method for Classifying Different Human Tissues with Quantitatively Tissue-Specific Expressed Genes
Source: Genes (Basel). 2018 Sep 7;9(9):449. doi: 10.3390/genes9090449 (PMC6162521; doi:10.3390/genes9090449)
Supplement: Supplementary file 1 [file genes-09-00449-s001.zip › Supplementary-Material-S4.docx]

**Supplementary Material S4.** Tissue enriched genes retrieved from Human Protein Atlas.

√: This symbol indicates that the gene is included in GTEx.

×: This symbol indicates that the gene is not included in GTEx.

| **Gene symbol** | **Ensembl ID** | **RNA tissue category** | **Present in GTEx** |
| --- | --- | --- | --- |
| WNT16 | ENSG00000002745 | Tissue enriched | √ |
| AC004381.6 | ENSG00000005189 | Tissue enriched | × |
| MPO | ENSG00000005381 | Tissue enriched | √ |
| PON1 | ENSG00000005421 | Tissue enriched | √ |
| ABCB4 | ENSG00000005471 | Tissue enriched | √ |
| SOX8 | ENSG00000005513 | Tissue enriched | √ |
| YBX2 | ENSG00000006047 | Tissue enriched | √ |
| CACNG3 | ENSG00000006116 | Tissue enriched | √ |
| LGALS14 | ENSG00000006659 | Tissue enriched | √ |
| UPP2 | ENSG00000007001 | Tissue enriched | √ |
| PRSS21 | ENSG00000007038 | Tissue enriched | √ |
| DNAH9 | ENSG00000007174 | Tissue enriched | √ |
| TKTL1 | ENSG00000007350 | Tissue enriched | √ |
| CACNA2D2 | ENSG00000007402 | Tissue enriched | √ |
| FMO3 | ENSG00000007933 | Tissue enriched | √ |
| SYN1 | ENSG00000008056 | Tissue enriched | √ |
| PGLYRP1 | ENSG00000008438 | Tissue enriched | √ |
| MAPK8IP2 | ENSG00000008735 | Tissue enriched | √ |
| MASP2 | ENSG00000009724 | Tissue enriched | √ |
| IYD | ENSG00000009765 | Tissue enriched | √ |
| PHF7 | ENSG00000010318 | Tissue enriched | √ |
| SLC6A13 | ENSG00000010379 | Tissue enriched | √ |
| PRSS3 | ENSG00000010438 | Tissue enriched | √ |
| FMO1 | ENSG00000010932 | Tissue enriched | √ |
| DPF1 | ENSG00000011332 | Tissue enriched | √ |
| ZBTB32 | ENSG00000011590 | Tissue enriched | √ |
| GABRA3 | ENSG00000011677 | Tissue enriched | √ |
| SLC7A14 | ENSG00000013293 | Tissue enriched | √ |
| ACPP | ENSG00000014257 | Tissue enriched | √ |
| STMN4 | ENSG00000015592 | Tissue enriched | √ |
| HGF | ENSG00000019991 | Tissue enriched | √ |
| NCDN | ENSG00000020129 | Tissue enriched | √ |
| CYP3A43 | ENSG00000021461 | Tissue enriched | √ |
| C8B | ENSG00000021852 | Tissue enriched | √ |
| GABRA1 | ENSG00000022355 | Tissue enriched | √ |
| HSD17B6 | ENSG00000025423 | Tissue enriched | √ |
| TBPL1 | ENSG00000028839 | Tissue enriched | √ |
| CYP46A1 | ENSG00000036530 | Tissue enriched | √ |
| SLC18A1 | ENSG00000036565 | Tissue enriched | √ |
| C6 | ENSG00000039537 | Tissue enriched | √ |
| SOX30 | ENSG00000039600 | Tissue enriched | √ |
| ZPBP | ENSG00000042813 | Tissue enriched | √ |
| TG | ENSG00000042832 | Tissue enriched | √ |
| DCUN1D1 | ENSG00000043093 | Tissue enriched | √ |
| GPM6B | ENSG00000046653 | Tissue enriched | √ |
| MAGEC2 | ENSG00000046774 | Tissue enriched | √ |
| CP | ENSG00000047457 | Tissue enriched | √ |
| ROS1 | ENSG00000047936 | Tissue enriched | √ |
| GUCA1A | ENSG00000048545 | Tissue enriched | √ |
| FOXC1 | ENSG00000054598 | Tissue enriched | √ |
| SPO11 | ENSG00000054796 | Tissue enriched | √ |
| MCOLN3 | ENSG00000055732 | Tissue enriched | √ |
| ITIH4 | ENSG00000055955 | Tissue enriched | √ |
| ITIH1 | ENSG00000055957 | Tissue enriched | √ |
| NPFFR2 | ENSG00000056291 | Tissue enriched | √ |
| GYG2 | ENSG00000056998 | Tissue enriched | √ |
| SERPINB3 | ENSG00000057149 | Tissue enriched | √ |
| SOAT1 | ENSG00000057252 | Tissue enriched | √ |
| MSH4 | ENSG00000057468 | Tissue enriched | √ |
| F7 | ENSG00000057593 | Tissue enriched | √ |
| SEZ6 | ENSG00000063015 | Tissue enriched | √ |
| SLC6A16 | ENSG00000063127 | Tissue enriched | √ |
| AHRR | ENSG00000063438 | Tissue enriched | √ |
| GSC2 | ENSG00000063515 | Tissue enriched | √ |
| DDX20 | ENSG00000064703 | Tissue enriched | √ |
| ROPN1 | ENSG00000065371 | Tissue enriched | √ |
| COL17A1 | ENSG00000065618 | Tissue enriched | √ |
| CACNB1 | ENSG00000067191 | Tissue enriched | √ |
| SYT1 | ENSG00000067715 | Tissue enriched | √ |
| PDZD4 | ENSG00000067840 | Tissue enriched | √ |
| FGFR3 | ENSG00000068078 | Tissue enriched | √ |
| PYGM | ENSG00000068976 | Tissue enriched | √ |
| PAGE1 | ENSG00000068985 | Tissue enriched | √ |
| FGF22 | ENSG00000070388 | Tissue enriched | √ |
| CHAT | ENSG00000070748 | Tissue enriched | √ |
| SLC12A3 | ENSG00000070915 | Tissue enriched | √ |
| RDH11 | ENSG00000072042 | Tissue enriched | √ |
| SPP2 | ENSG00000072080 | Tissue enriched | √ |
| FCGR2B | ENSG00000072694 | Tissue enriched | √ |
| TMEM38A | ENSG00000072954 | Tissue enriched | √ |
| MOV10L1 | ENSG00000073146 | Tissue enriched | √ |
| FNDC8 | ENSG00000073598 | Tissue enriched | √ |
| CD5L | ENSG00000073754 | Tissue enriched | √ |
| NSF | ENSG00000073969 | Tissue enriched | √ |
| SNCB | ENSG00000074317 | Tissue enriched | √ |
| SLC12A1 | ENSG00000074803 | Tissue enriched | √ |
| TUBA3D | ENSG00000075886 | Tissue enriched | √ |
| ACACB | ENSG00000076555 | Tissue enriched | √ |
| ACTL6B | ENSG00000077080 | Tissue enriched | √ |
| CAPN6 | ENSG00000077274 | Tissue enriched | √ |
| TYR | ENSG00000077498 | Tissue enriched | √ |
| FKBP6 | ENSG00000077800 | Tissue enriched | √ |
| SMC1B | ENSG00000077935 | Tissue enriched | √ |
| CST7 | ENSG00000077984 | Tissue enriched | √ |
| MAP2 | ENSG00000078018 | Tissue enriched | √ |
| PIAS2 | ENSG00000078043 | Tissue enriched | √ |
| AMPH | ENSG00000078053 | Tissue enriched | √ |
| LAMP3 | ENSG00000078081 | Tissue enriched | √ |
| ZCWPW1 | ENSG00000078487 | Tissue enriched | √ |
| BRINP1 | ENSG00000078725 | Tissue enriched | × |
| PKD2L2 | ENSG00000078795 | Tissue enriched | √ |
| SLC1A3 | ENSG00000079215 | Tissue enriched | √ |
| DUSP13 | ENSG00000079393 | Tissue enriched | √ |
| LIPE | ENSG00000079435 | Tissue enriched | √ |
| AFM | ENSG00000079557 | Tissue enriched | √ |
| DDX43 | ENSG00000080007 | Tissue enriched | √ |
| DCT | ENSG00000080166 | Tissue enriched | √ |
| CPB2 | ENSG00000080618 | Tissue enriched | √ |
| CHRNA3 | ENSG00000080644 | Tissue enriched | √ |
| CFHR2 | ENSG00000080910 | Tissue enriched | √ |
| IMPG2 | ENSG00000081148 | Tissue enriched | √ |
| CACNA1S | ENSG00000081248 | Tissue enriched | √ |
| KCNK2 | ENSG00000082482 | Tissue enriched | √ |
| EPYC | ENSG00000083782 | Tissue enriched | √ |
| SLC27A5 | ENSG00000083807 | Tissue enriched | √ |
| AGBL5 | ENSG00000084693 | Tissue enriched | √ |
| EFR3B | ENSG00000084710 | Tissue enriched | √ |
| KIF3C | ENSG00000084731 | Tissue enriched | √ |
| OVGP1 | ENSG00000085465 | Tissue enriched | √ |
| AKR1B1 | ENSG00000085662 | Tissue enriched | √ |
| AQP6 | ENSG00000086159 | Tissue enriched | √ |
| HBQ1 | ENSG00000086506 | Tissue enriched | √ |
| MYBPC2 | ENSG00000086967 | Tissue enriched | √ |
| NOX4 | ENSG00000086991 | Tissue enriched | √ |
| ACHE | ENSG00000087085 | Tissue enriched | √ |
| TMPRSS11E | ENSG00000087128 | Tissue enriched | × |
| MT3 | ENSG00000087250 | Tissue enriched | √ |
| GNAO1 | ENSG00000087258 | Tissue enriched | √ |
| F11 | ENSG00000088926 | Tissue enriched | √ |
| RPH3A | ENSG00000089169 | Tissue enriched | √ |
| CHGB | ENSG00000089199 | Tissue enriched | √ |
| NOS1 | ENSG00000089250 | Tissue enriched | √ |
| CMTM1 | ENSG00000089505 | Tissue enriched | √ |
| FETUB | ENSG00000090512 | Tissue enriched | √ |
| THPO | ENSG00000090534 | Tissue enriched | √ |
| RAB11FIP3 | ENSG00000090565 | Tissue enriched | √ |
| DLL3 | ENSG00000090932 | Tissue enriched | √ |
| LAMB4 | ENSG00000091128 | Tissue enriched | √ |
| SLC26A4 | ENSG00000091137 | Tissue enriched | √ |
| TF | ENSG00000091513 | Tissue enriched | √ |
| APOH | ENSG00000091583 | Tissue enriched | √ |
| SLC17A6 | ENSG00000091664 | Tissue enriched | √ |
| CPA1 | ENSG00000091704 | Tissue enriched | √ |
| CEBPE | ENSG00000092067 | Tissue enriched | √ |
| RPGRIP1 | ENSG00000092200 | Tissue enriched | √ |
| TGM1 | ENSG00000092295 | Tissue enriched | √ |
| DAZL | ENSG00000092345 | Tissue enriched | √ |
| TBL1Y | ENSG00000092377 | Tissue enriched | √ |
| CYP26A1 | ENSG00000095596 | Tissue enriched | √ |
| TDRD1 | ENSG00000095627 | Tissue enriched | √ |
| MYO3A | ENSG00000095777 | Tissue enriched | √ |
| PGC | ENSG00000096088 | Tissue enriched | √ |
| MLN | ENSG00000096395 | Tissue enriched | √ |
| CATSPERG | ENSG00000099338 | Tissue enriched | √ |
| STX1B | ENSG00000099365 | Tissue enriched | √ |
| MAGEB2 | ENSG00000099399 | Tissue enriched | √ |
| IZUMO4 | ENSG00000099840 | Tissue enriched | × |
| SERPIND1 | ENSG00000099937 | Tissue enriched | √ |
| CRYBB3 | ENSG00000100053 | Tissue enriched | √ |
| 3×Sep | ENSG00000100167 | Tissue enriched | × |
| C22orf31 | ENSG00000100249 | Tissue enriched | √ |
| MIOX | ENSG00000100253 | Tissue enriched | √ |
| RASL10A | ENSG00000100276 | Tissue enriched | √ |
| NEFH | ENSG00000100285 | Tissue enriched | √ |
| ACR | ENSG00000100312 | Tissue enriched | √ |
| CTSG | ENSG00000100448 | Tissue enriched | √ |
| COCH | ENSG00000100473 | Tissue enriched | √ |
| TRIM9 | ENSG00000100505 | Tissue enriched | √ |
| C14orf166B | ENSG00000100565 | Tissue enriched | √ |
| ISM2 | ENSG00000100593 | Tissue enriched | √ |
| SLC10A1 | ENSG00000100652 | Tissue enriched | √ |
| CPNE6 | ENSG00000100884 | Tissue enriched | √ |
| HRH3 | ENSG00000101180 | Tissue enriched | √ |
| MRGBP | ENSG00000101189 | Tissue enriched | × |
| MYLK2 | ENSG00000101306 | Tissue enriched | √ |
| HAO1 | ENSG00000101323 | Tissue enriched | √ |
| PDYN | ENSG00000101327 | Tissue enriched | √ |
| CCM2L | ENSG00000101331 | Tissue enriched | × |
| BPI | ENSG00000101425 | Tissue enriched | √ |
| CST9L | ENSG00000101435 | Tissue enriched | √ |
| SLC32A1 | ENSG00000101438 | Tissue enriched | √ |
| CST4 | ENSG00000101441 | Tissue enriched | √ |
| EPPIN | ENSG00000101448 | Tissue enriched | × |
| TNNC2 | ENSG00000101470 | Tissue enriched | √ |
| CELF4 | ENSG00000101489 | Tissue enriched | √ |
| H2BFM | ENSG00000101812 | Tissue enriched | √ |
| RHOXF1 | ENSG00000101883 | Tissue enriched | √ |
| ATP1B4 | ENSG00000101892 | Tissue enriched | √ |
| PAGE4 | ENSG00000101951 | Tissue enriched | √ |
| F9 | ENSG00000101981 | Tissue enriched | √ |
| SYP | ENSG00000102003 | Tissue enriched | √ |
| LUZP4 | ENSG00000102021 | Tissue enriched | √ |
| TEX28P2 | ENSG00000102080 | Tissue enriched | × |
| GATA1 | ENSG00000102145 | Tissue enriched | √ |
| VGLL1 | ENSG00000102243 | Tissue enriched | √ |
| DRP2 | ENSG00000102385 | Tissue enriched | √ |
| TAF7L | ENSG00000102387 | Tissue enriched | √ |
| HTR2A | ENSG00000102468 | Tissue enriched | √ |
| FLT1 | ENSG00000102755 | Tissue enriched | √ |
| IRG1 | ENSG00000102794 | Tissue enriched | × |
| MSLN | ENSG00000102854 | Tissue enriched | √ |
| PRSS54 | ENSG00000103023 | Tissue enriched | √ |
| PRSS33 | ENSG00000103355 | Tissue enriched | √ |
| SCG3 | ENSG00000104112 | Tissue enriched | √ |
| CALB1 | ENSG00000104327 | Tissue enriched | √ |
| JPH1 | ENSG00000104369 | Tissue enriched | √ |
| GML | ENSG00000104499 | Tissue enriched | √ |
| ADAM2 | ENSG00000104755 | Tissue enriched | √ |
| TULP2 | ENSG00000104804 | Tissue enriched | √ |
| CGB2 | ENSG00000104818 | Tissue enriched | √ |
| CGB | ENSG00000104827 | Tissue enriched | √ |
| KCNA7 | ENSG00000104848 | Tissue enriched | √ |
| CKM | ENSG00000104879 | Tissue enriched | √ |
| SLC17A7 | ENSG00000104888 | Tissue enriched | √ |
| DKKL1 | ENSG00000104901 | Tissue enriched | √ |
| RETN | ENSG00000104918 | Tissue enriched | √ |
| RSPH6A | ENSG00000104941 | Tissue enriched | √ |
| TNNT1 | ENSG00000105048 | Tissue enriched | √ |
| CASP14 | ENSG00000105141 | Tissue enriched | √ |
| AURKC | ENSG00000105146 | Tissue enriched | √ |
| LGALS13 | ENSG00000105198 | Tissue enriched | √ |
| EBI3 | ENSG00000105246 | Tissue enriched | √ |
| OVOL3 | ENSG00000105261 | Tissue enriched | × |
| APLP1 | ENSG00000105290 | Tissue enriched | √ |
| ZNRF4 | ENSG00000105428 | Tissue enriched | √ |
| SYNGR4 | ENSG00000105467 | Tissue enriched | √ |
| CLEC11A | ENSG00000105472 | Tissue enriched | √ |
| SIGLEC6 | ENSG00000105492 | Tissue enriched | √ |
| CAPS | ENSG00000105519 | Tissue enriched | √ |
| THEG | ENSG00000105549 | Tissue enriched | √ |
| FGF21 | ENSG00000105550 | Tissue enriched | √ |
| CACNG7 | ENSG00000105605 | Tissue enriched | √ |
| KLF1 | ENSG00000105610 | Tissue enriched | √ |
| MAST1 | ENSG00000105613 | Tissue enriched | √ |
| KCNN1 | ENSG00000105642 | Tissue enriched | √ |
| RAB3A | ENSG00000105649 | Tissue enriched | √ |
| ATP4A | ENSG00000105675 | Tissue enriched | √ |
| GAPDHS | ENSG00000105679 | Tissue enriched | √ |
| TMEM59L | ENSG00000105696 | Tissue enriched | √ |
| HAMP | ENSG00000105697 | Tissue enriched | √ |
| TFPI2 | ENSG00000105825 | Tissue enriched | √ |
| PON3 | ENSG00000105852 | Tissue enriched | √ |
| RNF32 | ENSG00000105982 | Tissue enriched | √ |
| ANKRD7 | ENSG00000106013 | Tissue enriched | √ |
| EPHB6 | ENSG00000106123 | Tissue enriched | √ |
| SPAM1 | ENSG00000106304 | Tissue enriched | √ |
| TFR2 | ENSG00000106327 | Tissue enriched | √ |
| FSCN3 | ENSG00000106328 | Tissue enriched | √ |
| FBXO24 | ENSG00000106336 | Tissue enriched | √ |
| AGFG2 | ENSG00000106351 | Tissue enriched | √ |
| MEST | ENSG00000106484 | Tissue enriched | √ |
| MYL7 | ENSG00000106631 | Tissue enriched | √ |
| TBL2 | ENSG00000106638 | Tissue enriched | √ |
| GALNTL5 | ENSG00000106648 | Tissue enriched | √ |
| LHX2 | ENSG00000106689 | Tissue enriched | √ |
| C5 | ENSG00000106804 | Tissue enriched | √ |
| AMBP | ENSG00000106927 | Tissue enriched | √ |
| DNM1 | ENSG00000106976 | Tissue enriched | √ |
| RLN1 | ENSG00000107018 | Tissue enriched | √ |
| SH3GL2 | ENSG00000107295 | Tissue enriched | √ |
| DNTT | ENSG00000107447 | Tissue enriched | √ |
| PITX3 | ENSG00000107859 | Tissue enriched | √ |
| LGI1 | ENSG00000108231 | Tissue enriched | √ |
| RUNDC3A | ENSG00000108309 | Tissue enriched | √ |
| WNT3 | ENSG00000108379 | Tissue enriched | √ |
| ENO3 | ENSG00000108515 | Tissue enriched | √ |
| CHRNE | ENSG00000108556 | Tissue enriched | √ |
| KRT32 | ENSG00000108759 | Tissue enriched | √ |
| HSD17B1 | ENSG00000108786 | Tissue enriched | √ |
| DLX4 | ENSG00000108813 | Tissue enriched | √ |
| PPY | ENSG00000108849 | Tissue enriched | √ |
| CACNG1 | ENSG00000108878 | Tissue enriched | √ |
| MYH1 | ENSG00000109061 | Tissue enriched | √ |
| VTN | ENSG00000109072 | Tissue enriched | √ |
| PHOX2B | ENSG00000109132 | Tissue enriched | √ |
| UGT2B10 | ENSG00000109181 | Tissue enriched | √ |
| ODAM | ENSG00000109205 | Tissue enriched | √ |
| SMR3A | ENSG00000109208 | Tissue enriched | √ |
| UCP1 | ENSG00000109424 | Tissue enriched | √ |
| ANXA10 | ENSG00000109511 | Tissue enriched | √ |
| HGFAC | ENSG00000109758 | Tissue enriched | √ |
| B3GAT1 | ENSG00000109956 | Tissue enriched | √ |
| NRXN2 | ENSG00000110076 | Tissue enriched | √ |
| HPX | ENSG00000110169 | Tissue enriched | √ |
| APOA5 | ENSG00000110243 | Tissue enriched | √ |
| UPK2 | ENSG00000110375 | Tissue enriched | √ |
| KIAA1549L | ENSG00000110427 | Tissue enriched | × |
| SLC1A2 | ENSG00000110436 | Tissue enriched | √ |
| SCGB2A2 | ENSG00000110484 | Tissue enriched | √ |
| CALCA | ENSG00000110680 | Tissue enriched | √ |
| PTPN5 | ENSG00000110786 | Tissue enriched | √ |
| ASIC1 | ENSG00000110881 | Tissue enriched | × |
| MYF6 | ENSG00000111046 | Tissue enriched | √ |
| PRR4 | ENSG00000111215 | Tissue enriched | √ |
| PRMT8 | ENSG00000111218 | Tissue enriched | √ |
| FGF6 | ENSG00000111241 | Tissue enriched | √ |
| AKAP3 | ENSG00000111254 | Tissue enriched | √ |
| KCNA1 | ENSG00000111262 | Tissue enriched | √ |
| GSG1 | ENSG00000111305 | Tissue enriched | √ |
| ACRBP | ENSG00000111644 | Tissue enriched | √ |
| SLCO1B3 | ENSG00000111700 | Tissue enriched | √ |
| GYS2 | ENSG00000111713 | Tissue enriched | √ |
| RSPH4A | ENSG00000111834 | Tissue enriched | √ |
| SLC26A8 | ENSG00000112053 | Tissue enriched | √ |
| RHAG | ENSG00000112077 | Tissue enriched | √ |
| TSPO2 | ENSG00000112212 | Tissue enriched | √ |
| SIM1 | ENSG00000112246 | Tissue enriched | √ |
| HDGFL1 | ENSG00000112273 | Tissue enriched | √ |
| B3GAT2 | ENSG00000112309 | Tissue enriched | √ |
| NR2E1 | ENSG00000112333 | Tissue enriched | √ |
| SLC17A2 | ENSG00000112337 | Tissue enriched | √ |
| SLC22A2 | ENSG00000112499 | Tissue enriched | √ |
| SLC4A9 | ENSG00000113073 | Tissue enriched | √ |
| PDE8B | ENSG00000113231 | Tissue enriched | √ |
| GABRG2 | ENSG00000113327 | Tissue enriched | √ |
| NUP155 | ENSG00000113569 | Tissue enriched | √ |
| C9 | ENSG00000113600 | Tissue enriched | √ |
| HRG | ENSG00000113905 | Tissue enriched | √ |
| CLDN16 | ENSG00000113946 | Tissue enriched | √ |
| SERPINI2 | ENSG00000114204 | Tissue enriched | √ |
| COL7A1 | ENSG00000114270 | Tissue enriched | √ |
| FGF12 | ENSG00000114279 | Tissue enriched | √ |
| MORC1 | ENSG00000114487 | Tissue enriched | √ |
| ROPN1B | ENSG00000114547 | Tissue enriched | √ |
| CSPG5 | ENSG00000114646 | Tissue enriched | √ |
| KIAA1257 | ENSG00000114656 | Tissue enriched | √ |
| PEX5L | ENSG00000114757 | Tissue enriched | √ |
| PCSK4 | ENSG00000115257 | Tissue enriched | √ |
| GCG | ENSG00000115263 | Tissue enriched | √ |
| APC2 | ENSG00000115266 | Tissue enriched | √ |
| KCNJ13 | ENSG00000115474 | Tissue enriched | √ |
| PLCD4 | ENSG00000115556 | Tissue enriched | √ |
| PRKAG3 | ENSG00000115592 | Tissue enriched | √ |
| IL1RL1 | ENSG00000115602 | Tissue enriched | √ |
| TPO | ENSG00000115705 | Tissue enriched | √ |
| PROC | ENSG00000115718 | Tissue enriched | √ |
| TNR | ENSG00000116147 | Tissue enriched | √ |
| PAPPA2 | ENSG00000116183 | Tissue enriched | √ |
| NPHS2 | ENSG00000116218 | Tissue enriched | √ |
| SWT1 | ENSG00000116668 | Tissue enriched | × |
| DNAJC6 | ENSG00000116675 | Tissue enriched | √ |
| PRG4 | ENSG00000116690 | Tissue enriched | √ |
| AMPD1 | ENSG00000116748 | Tissue enriched | √ |
| TNNI3K | ENSG00000116783 | Tissue enriched | √ |
| CFHR3 | ENSG00000116785 | Tissue enriched | √ |
| HPCAL4 | ENSG00000116983 | Tissue enriched | √ |
| RIMS3 | ENSG00000117016 | Tissue enriched | √ |
| KDM5B | ENSG00000117139 | Tissue enriched | √ |
| ACTL8 | ENSG00000117148 | Tissue enriched | √ |
| FMO6P | ENSG00000117507 | Tissue enriched | √ |
| HSD11B1 | ENSG00000117594 | Tissue enriched | √ |
| LPPR4 | ENSG00000117600 | Tissue enriched | √ |
| SERPINC1 | ENSG00000117601 | Tissue enriched | √ |
| NEK2 | ENSG00000117650 | Tissue enriched | √ |
| MUC5B | ENSG00000117983 | Tissue enriched | √ |
| A4GNT | ENSG00000118017 | Tissue enriched | √ |
| MMP8 | ENSG00000118113 | Tissue enriched | √ |
| ZNF541 | ENSG00000118156 | Tissue enriched | √ |
| SLC8A2 | ENSG00000118160 | Tissue enriched | √ |
| TNNT2 | ENSG00000118194 | Tissue enriched | √ |
| CRYGD | ENSG00000118231 | Tissue enriched | √ |
| TNP1 | ENSG00000118245 | Tissue enriched | √ |
| TTR | ENSG00000118271 | Tissue enriched | √ |
| SPACA1 | ENSG00000118434 | Tissue enriched | √ |
| ZC2HC1B | ENSG00000118491 | Tissue enriched | × |
| PMFBP1 | ENSG00000118557 | Tissue enriched | √ |
| CASQ2 | ENSG00000118729 | Tissue enriched | √ |
| OLFM3 | ENSG00000118733 | Tissue enriched | √ |
| TRIM67 | ENSG00000119283 | Tissue enriched | √ |
| GPR75 | ENSG00000119737 | Tissue enriched | √ |
| CPN1 | ENSG00000120054 | Tissue enriched | √ |
| EQTN | ENSG00000120160 | Tissue enriched | × |
| INSL6 | ENSG00000120210 | Tissue enriched | √ |
| INSL4 | ENSG00000120211 | Tissue enriched | √ |
| MLANA | ENSG00000120215 | Tissue enriched | √ |
| GRIA2 | ENSG00000120251 | Tissue enriched | √ |
| CCDC170 | ENSG00000120262 | Tissue enriched | × |
| MAGEB4 | ENSG00000120289 | Tissue enriched | √ |
| SLC25A2 | ENSG00000120329 | Tissue enriched | √ |
| TTLL2 | ENSG00000120440 | Tissue enriched | √ |
| LYZL1 | ENSG00000120563 | Tissue enriched | √ |
| SOHLH2 | ENSG00000120669 | Tissue enriched | √ |
| MYOT | ENSG00000120729 | Tissue enriched | √ |
| CHRNA2 | ENSG00000120903 | Tissue enriched | √ |
| NPPB | ENSG00000120937 | Tissue enriched | √ |
| EPX | ENSG00000121053 | Tissue enriched | √ |
| COIL | ENSG00000121058 | Tissue enriched | √ |
| TEX14 | ENSG00000121101 | Tissue enriched | √ |
| PRB2 | ENSG00000121335 | Tissue enriched | √ |
| IAPP | ENSG00000121351 | Tissue enriched | √ |
| RP11×408E5.4 | ENSG00000121388 | Tissue enriched | × |
| A1BG | ENSG00000121410 | Tissue enriched | √ |
| RGSL1 | ENSG00000121446 | Tissue enriched | √ |
| POPDC2 | ENSG00000121577 | Tissue enriched | √ |
| GJA8 | ENSG00000121634 | Tissue enriched | √ |
| BAI2 | ENSG00000121753 | Tissue enriched | √ |
| HPCA | ENSG00000121905 | Tissue enriched | √ |
| MYOG | ENSG00000122180 | Tissue enriched | √ |
| PLG | ENSG00000122194 | Tissue enriched | √ |
| HS3ST2 | ENSG00000122254 | Tissue enriched | √ |
| PRM2 | ENSG00000122304 | Tissue enriched | √ |
| AKR1D1 | ENSG00000122787 | Tissue enriched | √ |
| SFTPA1 | ENSG00000122852 | Tissue enriched | √ |
| NECAB1 | ENSG00000123119 | Tissue enriched | √ |
| ACTRT1 | ENSG00000123165 | Tissue enriched | √ |
| CCDC70 | ENSG00000123171 | Tissue enriched | √ |
| NEUROD4 | ENSG00000123307 | Tissue enriched | √ |
| HOXC13 | ENSG00000123364 | Tissue enriched | √ |
| NFE2 | ENSG00000123405 | Tissue enriched | √ |
| DBH | ENSG00000123454 | Tissue enriched | √ |
| PLP1 | ENSG00000123560 | Tissue enriched | √ |
| SERPINA7 | ENSG00000123561 | Tissue enriched | √ |
| ESX1 | ENSG00000123576 | Tissue enriched | √ |
| MAGEA9B | ENSG00000123584 | Tissue enriched | √ |
| ATXN3L | ENSG00000123594 | Tissue enriched | √ |
| ACVR1C | ENSG00000123612 | Tissue enriched | √ |
| C4BPA | ENSG00000123838 | Tissue enriched | √ |
| MOGAT1 | ENSG00000124003 | Tissue enriched | √ |
| CTCFL | ENSG00000124092 | Tissue enriched | √ |
| FAM209A | ENSG00000124103 | Tissue enriched | × |
| WFDC3 | ENSG00000124116 | Tissue enriched | √ |
| SLC12A5 | ENSG00000124140 | Tissue enriched | √ |
| GDAP1L1 | ENSG00000124194 | Tissue enriched | √ |
| GTSF1L | ENSG00000124196 | Tissue enriched | √ |
| ANKRD60 | ENSG00000124227 | Tissue enriched | × |
| RBPJL | ENSG00000124232 | Tissue enriched | √ |
| C20orf85 | ENSG00000124237 | Tissue enriched | √ |
| TP53TG5 | ENSG00000124251 | Tissue enriched | √ |
| MAGEA10 | ENSG00000124260 | Tissue enriched | √ |
| IRGC | ENSG00000124449 | Tissue enriched | √ |
| PSG8 | ENSG00000124467 | Tissue enriched | √ |
| CEACAM8 | ENSG00000124469 | Tissue enriched | √ |
| CRISP2 | ENSG00000124490 | Tissue enriched | √ |
| PACSIN1 | ENSG00000124507 | Tissue enriched | √ |
| HIST1H1D | ENSG00000124575 | Tissue enriched | √ |
| TCP11 | ENSG00000124678 | Tissue enriched | √ |
| HIST1H3B | ENSG00000124693 | Tissue enriched | √ |
| DNAH8 | ENSG00000124721 | Tissue enriched | √ |
| CRISP1 | ENSG00000124812 | Tissue enriched | √ |
| OPN5 | ENSG00000124818 | Tissue enriched | √ |
| EREG | ENSG00000124882 | Tissue enriched | √ |
| PIWIL1 | ENSG00000125207 | Tissue enriched | √ |
| GRK4 | ENSG00000125388 | Tissue enriched | √ |
| MYH2 | ENSG00000125414 | Tissue enriched | √ |
| C1orf61 | ENSG00000125462 | Tissue enriched | √ |
| C20orf195 | ENSG00000125531 | Tissue enriched | √ |
| PLGLB2 | ENSG00000125551 | Tissue enriched | √ |
| IL37 | ENSG00000125571 | Tissue enriched | × |
| PAX8 | ENSG00000125618 | Tissue enriched | √ |
| C3 | ENSG00000125730 | Tissue enriched | √ |
| RTN2 | ENSG00000125744 | Tissue enriched | √ |
| TGM3 | ENSG00000125780 | Tissue enriched | √ |
| PAX1 | ENSG00000125813 | Tissue enriched | √ |
| NAPB | ENSG00000125814 | Tissue enriched | √ |
| CST8 | ENSG00000125815 | Tissue enriched | √ |
| CSTL1 | ENSG00000125823 | Tissue enriched | √ |
| LRRN4 | ENSG00000125872 | Tissue enriched | √ |
| BANF2 | ENSG00000125888 | Tissue enriched | √ |
| C20orf187 | ENSG00000125899 | Tissue enriched | × |
| SIRPD | ENSG00000125900 | Tissue enriched | √ |
| CITED1 | ENSG00000125931 | Tissue enriched | √ |
| GDF5 | ENSG00000125965 | Tissue enriched | √ |
| C20orf173 | ENSG00000125975 | Tissue enriched | √ |
| GRPR | ENSG00000126010 | Tissue enriched | √ |
| KIRREL2 | ENSG00000126259 | Tissue enriched | √ |
| TSKS | ENSG00000126467 | Tissue enriched | √ |
| CSN1S1 | ENSG00000126545 | Tissue enriched | √ |
| STATH | ENSG00000126549 | Tissue enriched | √ |
| HTN1 | ENSG00000126550 | Tissue enriched | √ |
| PRKCG | ENSG00000126583 | Tissue enriched | √ |
| SSX1 | ENSG00000126752 | Tissue enriched | √ |
| PRDM7 | ENSG00000126856 | Tissue enriched | √ |
| NXF5 | ENSG00000126952 | Tissue enriched | √ |
| CRYGN | ENSG00000127377 | Tissue enriched | √ |
| TRPV5 | ENSG00000127412 | Tissue enriched | √ |
| GNGT1 | ENSG00000127928 | Tissue enriched | √ |
| SPINK2 | ENSG00000128040 | Tissue enriched | √ |
| RFPL2 | ENSG00000128253 | Tissue enriched | √ |
| RFPL3 | ENSG00000128276 | Tissue enriched | √ |
| C22orf23 | ENSG00000128346 | Tissue enriched | √ |
| CPA4 | ENSG00000128510 | Tissue enriched | √ |
| CDHR3 | ENSG00000128536 | Tissue enriched | √ |
| VGF | ENSG00000128564 | Tissue enriched | √ |
| CCDC136 | ENSG00000128596 | Tissue enriched | √ |
| FEZF1 | ENSG00000128610 | Tissue enriched | √ |
| CHN1 | ENSG00000128656 | Tissue enriched | √ |
| GAD1 | ENSG00000128683 | Tissue enriched | √ |
| GDF2 | ENSG00000128802 | Tissue enriched | √ |
| TMOD2 | ENSG00000128872 | Tissue enriched | √ |
| MYOD1 | ENSG00000129152 | Tissue enriched | √ |
| KCNC1 | ENSG00000129159 | Tissue enriched | √ |
| ATP1B2 | ENSG00000129244 | Tissue enriched | √ |
| KLK14 | ENSG00000129437 | Tissue enriched | √ |
| FOXJ1 | ENSG00000129654 | Tissue enriched | √ |
| ART1 | ENSG00000129744 | Tissue enriched | √ |
| CHRNA10 | ENSG00000129749 | Tissue enriched | √ |
| VCY1B | ENSG00000129862 | Tissue enriched | × |
| VCY | ENSG00000129864 | Tissue enriched | √ |
| CDY2B | ENSG00000129873 | Tissue enriched | √ |
| CDH15 | ENSG00000129910 | Tissue enriched | √ |
| INS×IGF2 | ENSG00000129965 | Tissue enriched | √ |
| LBP | ENSG00000129988 | Tissue enriched | √ |
| SYT5 | ENSG00000129990 | Tissue enriched | √ |
| TNNI3 | ENSG00000129991 | Tissue enriched | √ |
| TSPAN16 | ENSG00000130167 | Tissue enriched | √ |
| C19orf80 | ENSG00000130173 | Tissue enriched | × |
| APOC1 | ENSG00000130208 | Tissue enriched | √ |
| NCAN | ENSG00000130287 | Tissue enriched | √ |
| KIF1A | ENSG00000130294 | Tissue enriched | √ |
| ACSBG2 | ENSG00000130377 | Tissue enriched | √ |
| SULT4A1 | ENSG00000130540 | Tissue enriched | √ |
| OLFM1 | ENSG00000130558 | Tissue enriched | √ |
| TNNT3 | ENSG00000130595 | Tissue enriched | √ |
| TNNI2 | ENSG00000130598 | Tissue enriched | √ |
| CYP2E1 | ENSG00000130649 | Tissue enriched | √ |
| CCDC62 | ENSG00000130783 | Tissue enriched | √ |
| SLC7A10 | ENSG00000130876 | Tissue enriched | √ |
| PKDREJ | ENSG00000130943 | Tissue enriched | √ |
| HSD17B3 | ENSG00000130948 | Tissue enriched | √ |
| NUTM2F | ENSG00000130950 | Tissue enriched | × |
| FBP2 | ENSG00000130957 | Tissue enriched | √ |
| BPIFA2 | ENSG00000131050 | Tissue enriched | × |
| BPIFA3 | ENSG00000131059 | Tissue enriched | × |
| C1QL1 | ENSG00000131094 | Tissue enriched | √ |
| GFAP | ENSG00000131095 | Tissue enriched | √ |
| TEX101 | ENSG00000131126 | Tissue enriched | √ |
| SLC34A1 | ENSG00000131183 | Tissue enriched | √ |
| F12 | ENSG00000131187 | Tissue enriched | √ |
| C3orf20 | ENSG00000131379 | Tissue enriched | √ |
| NAPSA | ENSG00000131400 | Tissue enriched | √ |
| LRRC4B | ENSG00000131409 | Tissue enriched | √ |
| KREMEN2 | ENSG00000131650 | Tissue enriched | √ |
| BARX1 | ENSG00000131668 | Tissue enriched | √ |
| CA6 | ENSG00000131686 | Tissue enriched | √ |
| RHOXF2 | ENSG00000131721 | Tissue enriched | × |
| USP29 | ENSG00000131864 | Tissue enriched | √ |
| LIN28A | ENSG00000131914 | Tissue enriched | √ |
| ABHD12B | ENSG00000131969 | Tissue enriched | √ |
| SPATA6 | ENSG00000132122 | Tissue enriched | √ |
| LHX1 | ENSG00000132130 | Tissue enriched | √ |
| GAS2L2 | ENSG00000132139 | Tissue enriched | √ |
| CCT6B | ENSG00000132141 | Tissue enriched | √ |
| FTHL17 | ENSG00000132446 | Tissue enriched | √ |
| SCP2D1 | ENSG00000132631 | Tissue enriched | × |
| SNAP25 | ENSG00000132639 | Tissue enriched | √ |
| BCAN | ENSG00000132692 | Tissue enriched | √ |
| HAPLN2 | ENSG00000132702 | Tissue enriched | √ |
| APCS | ENSG00000132703 | Tissue enriched | √ |
| SYT11 | ENSG00000132718 | Tissue enriched | √ |
| MTL5 | ENSG00000132749 | Tissue enriched | √ |
| ANGPTL3 | ENSG00000132855 | Tissue enriched | √ |
| MTUS2 | ENSG00000132938 | Tissue enriched | √ |
| TPTE2 | ENSG00000132958 | Tissue enriched | √ |
| RNF17 | ENSG00000132972 | Tissue enriched | √ |
| STOML3 | ENSG00000133115 | Tissue enriched | √ |
| KL | ENSG00000133116 | Tissue enriched | √ |
| TEX13A | ENSG00000133149 | Tissue enriched | √ |
| PRAM1 | ENSG00000133246 | Tissue enriched | √ |
| LGALS12 | ENSG00000133317 | Tissue enriched | √ |
| SFTPD | ENSG00000133661 | Tissue enriched | √ |
| DYDC2 | ENSG00000133665 | Tissue enriched | √ |
| ADAM20 | ENSG00000134007 | Tissue enriched | √ |
| GNAT2 | ENSG00000134183 | Tissue enriched | √ |
| ADAM30 | ENSG00000134249 | Tissue enriched | √ |
| CFHR4 | ENSG00000134365 | Tissue enriched | √ |
| CFHR5 | ENSG00000134389 | Tissue enriched | √ |
| SLCO1B1 | ENSG00000134538 | Tissue enriched | √ |
| PRH2 | ENSG00000134551 | Tissue enriched | √ |
| MYBPC3 | ENSG00000134571 | Tissue enriched | √ |
| SOX3 | ENSG00000134595 | Tissue enriched | √ |
| DSG1 | ENSG00000134760 | Tissue enriched | √ |
| DSC1 | ENSG00000134765 | Tissue enriched | √ |
| GIF | ENSG00000134812 | Tissue enriched | √ |
| GGACT | ENSG00000134864 | Tissue enriched | × |
| ACRV1 | ENSG00000134940 | Tissue enriched | √ |
| SDS | ENSG00000135094 | Tissue enriched | √ |
| CSN2 | ENSG00000135222 | Tissue enriched | √ |
| UGT2B28 | ENSG00000135226 | Tissue enriched | √ |
| FAM71F1 | ENSG00000135248 | Tissue enriched | √ |
| BAI3 | ENSG00000135298 | Tissue enriched | √ |
| CGA | ENSG00000135346 | Tissue enriched | √ |
| ELF5 | ENSG00000135374 | Tissue enriched | √ |
| LACRT | ENSG00000135413 | Tissue enriched | √ |
| GLS2 | ENSG00000135423 | Tissue enriched | √ |
| FAM186B | ENSG00000135436 | Tissue enriched | √ |
| B4GALNT1 | ENSG00000135454 | Tissue enriched | √ |
| EMX1 | ENSG00000135638 | Tissue enriched | √ |
| CHRND | ENSG00000135902 | Tissue enriched | √ |
| USP44 | ENSG00000136014 | Tissue enriched | √ |
| PCDH8 | ENSG00000136099 | Tissue enriched | √ |
| SPDYE1 | ENSG00000136206 | Tissue enriched | √ |
| MYCBPAP | ENSG00000136449 | Tissue enriched | √ |
| GH2 | ENSG00000136487 | Tissue enriched | √ |
| CSH1 | ENSG00000136488 | Tissue enriched | √ |
| SCN2A | ENSG00000136531 | Tissue enriched | √ |
| TBR1 | ENSG00000136535 | Tissue enriched | √ |
| ERMN | ENSG00000136541 | Tissue enriched | √ |
| IL36G | ENSG00000136688 | Tissue enriched | × |
| BIN1 | ENSG00000136717 | Tissue enriched | √ |
| GAD2 | ENSG00000136750 | Tissue enriched | √ |
| ODF2 | ENSG00000136811 | Tissue enriched | √ |
| STXBP1 | ENSG00000136854 | Tissue enriched | √ |
| SLC31A2 | ENSG00000136867 | Tissue enriched | √ |
| BAAT | ENSG00000136881 | Tissue enriched | √ |
| WDR38 | ENSG00000136918 | Tissue enriched | √ |
| GABBR2 | ENSG00000136928 | Tissue enriched | √ |
| LMX1B | ENSG00000136944 | Tissue enriched | √ |
| NOV | ENSG00000136999 | Tissue enriched | √ |
| DMRT1 | ENSG00000137090 | Tissue enriched | √ |
| IGFBPL1 | ENSG00000137142 | Tissue enriched | √ |
| CAPN11 | ENSG00000137225 | Tissue enriched | √ |
| TINAG | ENSG00000137251 | Tissue enriched | √ |
| TUBB2A | ENSG00000137267 | Tissue enriched | √ |
| GCM1 | ENSG00000137270 | Tissue enriched | √ |
| TUBB2B | ENSG00000137285 | Tissue enriched | √ |
| CLPS | ENSG00000137392 | Tissue enriched | √ |
| FGFBP1 | ENSG00000137440 | Tissue enriched | √ |
| MGARP | ENSG00000137463 | Tissue enriched | × |
| TTPA | ENSG00000137561 | Tissue enriched | √ |
| MMP20 | ENSG00000137674 | Tissue enriched | √ |
| BTG4 | ENSG00000137707 | Tissue enriched | √ |
| POU2F3 | ENSG00000137709 | Tissue enriched | √ |
| FDX1 | ENSG00000137714 | Tissue enriched | √ |
| CYP19A1 | ENSG00000137869 | Tissue enriched | √ |
| BRDT | ENSG00000137948 | Tissue enriched | √ |
| DNASE2B | ENSG00000137976 | Tissue enriched | √ |
| CYP2C9 | ENSG00000138109 | Tissue enriched | √ |
| CYP2C8 | ENSG00000138115 | Tissue enriched | √ |
| LBX1 | ENSG00000138136 | Tissue enriched | √ |
| CUZD1 | ENSG00000138161 | Tissue enriched | √ |
| RBP4 | ENSG00000138207 | Tissue enriched | √ |
| MSMB | ENSG00000138294 | Tissue enriched | √ |
| OIT3 | ENSG00000138315 | Tissue enriched | √ |
| CCDC54 | ENSG00000138483 | Tissue enriched | √ |
| APH1B | ENSG00000138613 | Tissue enriched | √ |
| C4orf17 | ENSG00000138813 | Tissue enriched | √ |
| FBN2 | ENSG00000138829 | Tissue enriched | √ |
| ERP27 | ENSG00000139055 | Tissue enriched | √ |
| PLCZ1 | ENSG00000139151 | Tissue enriched | √ |
| SLCO1C1 | ENSG00000139155 | Tissue enriched | √ |
| SLC38A4 | ENSG00000139209 | Tissue enriched | √ |
| INHBE | ENSG00000139269 | Tissue enriched | √ |
| SYCP3 | ENSG00000139351 | Tissue enriched | √ |
| ASCL1 | ENSG00000139352 | Tissue enriched | √ |
| FOXN4 | ENSG00000139445 | Tissue enriched | √ |
| PDX1 | ENSG00000139515 | Tissue enriched | √ |
| RDH16 | ENSG00000139547 | Tissue enriched | √ |
| DHH | ENSG00000139549 | Tissue enriched | √ |
| SMIM2 | ENSG00000139656 | Tissue enriched | × |
| SRRM4 | ENSG00000139767 | Tissue enriched | √ |
| RNF113B | ENSG00000139797 | Tissue enriched | √ |
| TSSK4 | ENSG00000139908 | Tissue enriched | √ |
| RTN1 | ENSG00000139970 | Tissue enriched | √ |
| ADAM21 | ENSG00000139985 | Tissue enriched | √ |
| SERPINA10 | ENSG00000140093 | Tissue enriched | √ |
| SLC25A47 | ENSG00000140107 | Tissue enriched | × |
| GOLGA6D | ENSG00000140478 | Tissue enriched | √ |
| CYP1A2 | ENSG00000140505 | Tissue enriched | √ |
| RLBP1 | ENSG00000140522 | Tissue enriched | √ |
| 12×Sep | ENSG00000140623 | Tissue enriched | × |
| SLC5A2 | ENSG00000140675 | Tissue enriched | √ |
| ABCC12 | ENSG00000140798 | Tissue enriched | √ |
| CHST4 | ENSG00000140835 | Tissue enriched | √ |
| ADAMTS18 | ENSG00000140873 | Tissue enriched | √ |
| CMTM2 | ENSG00000140932 | Tissue enriched | √ |
| ADAD2 | ENSG00000140955 | Tissue enriched | √ |
| CTRL | ENSG00000141086 | Tissue enriched | √ |
| DPEP3 | ENSG00000141096 | Tissue enriched | √ |
| KIF2B | ENSG00000141200 | Tissue enriched | √ |
| SPATA22 | ENSG00000141255 | Tissue enriched | √ |
| C17orf64 | ENSG00000141371 | Tissue enriched | √ |
| SLC25A52 | ENSG00000141437 | Tissue enriched | × |
| ASGR1 | ENSG00000141505 | Tissue enriched | √ |
| CBLN2 | ENSG00000141668 | Tissue enriched | √ |
| PNMT | ENSG00000141744 | Tissue enriched | √ |
| FEM1A | ENSG00000141965 | Tissue enriched | √ |
| DMRTC2 | ENSG00000142025 | Tissue enriched | √ |
| LMTK3 | ENSG00000142235 | Tissue enriched | √ |
| CACNG8 | ENSG00000142408 | Tissue enriched | √ |
| GPR32 | ENSG00000142511 | Tissue enriched | √ |
| KLK3 | ENSG00000142515 | Tissue enriched | √ |
| FAM71E1 | ENSG00000142530 | Tissue enriched | √ |
| CELA2A | ENSG00000142615 | Tissue enriched | √ |
| PADI1 | ENSG00000142623 | Tissue enriched | √ |
| C1orf94 | ENSG00000142698 | Tissue enriched | √ |
| CELA3A | ENSG00000142789 | Tissue enriched | √ |
| DMRTB1 | ENSG00000143006 | Tissue enriched | √ |
| SYPL2 | ENSG00000143028 | Tissue enriched | √ |
| BARHL2 | ENSG00000143032 | Tissue enriched | √ |
| FNDC7 | ENSG00000143107 | Tissue enriched | √ |
| RXRG | ENSG00000143171 | Tissue enriched | √ |
| MAEL | ENSG00000143194 | Tissue enriched | √ |
| ADCY10 | ENSG00000143199 | Tissue enriched | √ |
| NR1I3 | ENSG00000143257 | Tissue enriched | √ |
| F13B | ENSG00000143278 | Tissue enriched | √ |
| CASQ1 | ENSG00000143318 | Tissue enriched | √ |
| CRABP2 | ENSG00000143320 | Tissue enriched | √ |
| ECM1 | ENSG00000143369 | Tissue enriched | √ |
| SEMA6C | ENSG00000143434 | Tissue enriched | √ |
| C1orf56 | ENSG00000143443 | Tissue enriched | √ |
| OAZ3 | ENSG00000143450 | Tissue enriched | √ |
| HORMAD1 | ENSG00000143452 | Tissue enriched | √ |
| KCNH1 | ENSG00000143473 | Tissue enriched | √ |
| FLG2 | ENSG00000143520 | Tissue enriched | √ |
| CRNN | ENSG00000143536 | Tissue enriched | √ |
| TPM3 | ENSG00000143549 | Tissue enriched | √ |
| NUP210L | ENSG00000143552 | Tissue enriched | √ |
| FLG | ENSG00000143631 | Tissue enriched | √ |
| ACTA1 | ENSG00000143632 | Tissue enriched | √ |
| ABHD1 | ENSG00000143994 | Tissue enriched | √ |
| NAT8 | ENSG00000144035 | Tissue enriched | √ |
| DQX1 | ENSG00000144045 | Tissue enriched | √ |
| C1QL2 | ENSG00000144119 | Tissue enriched | √ |
| GPR17 | ENSG00000144230 | Tissue enriched | √ |
| SLC4A10 | ENSG00000144290 | Tissue enriched | √ |
| PTH2R | ENSG00000144407 | Tissue enriched | √ |
| CPO | ENSG00000144410 | Tissue enriched | √ |
| ABCA12 | ENSG00000144452 | Tissue enriched | √ |
| GADL1 | ENSG00000144644 | Tissue enriched | √ |
| ACKR2 | ENSG00000144648 | Tissue enriched | × |
| SLC22A14 | ENSG00000144671 | Tissue enriched | √ |
| TAGLN3 | ENSG00000144834 | Tissue enriched | √ |
| SPATA16 | ENSG00000144962 | Tissue enriched | √ |
| UCN2 | ENSG00000145040 | Tissue enriched | √ |
| AHSG | ENSG00000145192 | Tissue enriched | √ |
| VWA5B2 | ENSG00000145198 | Tissue enriched | √ |
| CORIN | ENSG00000145244 | Tissue enriched | √ |
| CABS1 | ENSG00000145309 | Tissue enriched | × |
| DDIT4L | ENSG00000145358 | Tissue enriched | √ |
| GLRA3 | ENSG00000145451 | Tissue enriched | √ |
| ANKRD31 | ENSG00000145700 | Tissue enriched | √ |
| LIX1 | ENSG00000145721 | Tissue enriched | √ |
| SPATA9 | ENSG00000145757 | Tissue enriched | √ |
| MEGF10 | ENSG00000145794 | Tissue enriched | √ |
| LECT2 | ENSG00000145826 | Tissue enriched | √ |
| GABRB2 | ENSG00000145864 | Tissue enriched | √ |
| TENM2 | ENSG00000145934 | Tissue enriched | × |
| FAM217A | ENSG00000145975 | Tissue enriched | × |
| PSD2 | ENSG00000146005 | Tissue enriched | √ |
| LRRTM2 | ENSG00000146006 | Tissue enriched | √ |
| HIST1H2BA | ENSG00000146047 | Tissue enriched | √ |
| SCUBE3 | ENSG00000146197 | Tissue enriched | √ |
| GABRR1 | ENSG00000146276 | Tissue enriched | √ |
| TAAR1 | ENSG00000146399 | Tissue enriched | √ |
| ASB10 | ENSG00000146926 | Tissue enriched | √ |
| TMEM27 | ENSG00000147003 | Tissue enriched | √ |
| AKAP4 | ENSG00000147081 | Tissue enriched | √ |
| CPXCR1 | ENSG00000147183 | Tissue enriched | √ |
| HTR2C | ENSG00000147246 | Tissue enriched | √ |
| ARHGAP36 | ENSG00000147256 | Tissue enriched | √ |
| GPC3 | ENSG00000147257 | Tissue enriched | √ |
| FATE1 | ENSG00000147378 | Tissue enriched | √ |
| MAGEA4 | ENSG00000147381 | Tissue enriched | √ |
| STAR | ENSG00000147465 | Tissue enriched | √ |
| ST18 | ENSG00000147488 | Tissue enriched | √ |
| DNAJC5B | ENSG00000147570 | Tissue enriched | √ |
| CRH | ENSG00000147571 | Tissue enriched | √ |
| PMP2 | ENSG00000147588 | Tissue enriched | √ |
| SLC26A7 | ENSG00000147606 | Tissue enriched | √ |
| ATP6V0D2 | ENSG00000147614 | Tissue enriched | √ |
| FAM83A | ENSG00000147689 | Tissue enriched | √ |
| SPATA31A3 | ENSG00000147926 | Tissue enriched | × |
| ACTL7B | ENSG00000148156 | Tissue enriched | √ |
| NR6A1 | ENSG00000148200 | Tissue enriched | √ |
| IDI2 | ENSG00000148377 | Tissue enriched | √ |
| SLC39A12 | ENSG00000148482 | Tissue enriched | √ |
| LRIT1 | ENSG00000148602 | Tissue enriched | √ |
| RGR | ENSG00000148604 | Tissue enriched | √ |
| ANKRD1 | ENSG00000148677 | Tissue enriched | √ |
| HABP2 | ENSG00000148702 | Tissue enriched | √ |
| CYP17A1 | ENSG00000148795 | Tissue enriched | √ |
| ADAM12 | ENSG00000148848 | Tissue enriched | √ |
| LRRC4C | ENSG00000148948 | Tissue enriched | √ |
| SAA4 | ENSG00000148965 | Tissue enriched | √ |
| SCGB1A1 | ENSG00000149021 | Tissue enriched | √ |
| C20orf78 | ENSG00000149443 | Tissue enriched | × |
| SLC22A8 | ENSG00000149452 | Tissue enriched | √ |
| ZP1 | ENSG00000149506 | Tissue enriched | √ |
| OOSP2 | ENSG00000149507 | Tissue enriched | × |
| MS4A3 | ENSG00000149516 | Tissue enriched | √ |
| FEZ1 | ENSG00000149557 | Tissue enriched | √ |
| KIRREL3 | ENSG00000149571 | Tissue enriched | √ |
| C20orf144 | ENSG00000149609 | Tissue enriched | √ |
| CNBD2 | ENSG00000149646 | Tissue enriched | × |
| CABLES2 | ENSG00000149679 | Tissue enriched | √ |
| GPHA2 | ENSG00000149735 | Tissue enriched | √ |
| SLC22A9 | ENSG00000149742 | Tissue enriched | √ |
| FAM57B | ENSG00000149926 | Tissue enriched | √ |
| CNKSR2 | ENSG00000149970 | Tissue enriched | √ |
| C10ORF68 | ENSG00000150076 | Tissue enriched | × |
| FXYD4 | ENSG00000150201 | Tissue enriched | √ |
| CDH8 | ENSG00000150394 | Tissue enriched | √ |
| LYPD1 | ENSG00000150551 | Tissue enriched | √ |
| GPM6A | ENSG00000150625 | Tissue enriched | √ |
| SPATA4 | ENSG00000150628 | Tissue enriched | √ |
| CCDC83 | ENSG00000150676 | Tissue enriched | √ |
| TEX12 | ENSG00000150783 | Tissue enriched | √ |
| C2orf50 | ENSG00000150873 | Tissue enriched | √ |
| TKTL2 | ENSG00000151005 | Tissue enriched | √ |
| ENKUR | ENSG00000151023 | Tissue enriched | √ |
| GPR158 | ENSG00000151025 | Tissue enriched | √ |
| LYZL2 | ENSG00000151033 | Tissue enriched | √ |
| KCNA6 | ENSG00000151079 | Tissue enriched | √ |
| RAD9B | ENSG00000151164 | Tissue enriched | √ |
| MAT1A | ENSG00000151224 | Tissue enriched | √ |
| TEX30 | ENSG00000151287 | Tissue enriched | × |
| ALLC | ENSG00000151360 | Tissue enriched | √ |
| ATP6V1G3 | ENSG00000151418 | Tissue enriched | √ |
| SLC25A31 | ENSG00000151475 | Tissue enriched | √ |
| NCAPD3 | ENSG00000151503 | Tissue enriched | √ |
| POU4F2 | ENSG00000151615 | Tissue enriched | √ |
| ITIH2 | ENSG00000151655 | Tissue enriched | √ |
| KCNJ1 | ENSG00000151704 | Tissue enriched | √ |
| TDO2 | ENSG00000151790 | Tissue enriched | √ |
| GABRA2 | ENSG00000151834 | Tissue enriched | √ |
| CCL28 | ENSG00000151882 | Tissue enriched | √ |
| BEND6 | ENSG00000151917 | Tissue enriched | √ |
| TMEM132D | ENSG00000151952 | Tissue enriched | √ |
| RBM46 | ENSG00000151962 | Tissue enriched | √ |
| MCHR2 | ENSG00000152034 | Tissue enriched | √ |
| TUBA3E | ENSG00000152086 | Tissue enriched | √ |
| GRID2 | ENSG00000152208 | Tissue enriched | √ |
| RIT2 | ENSG00000152214 | Tissue enriched | √ |
| G6PC2 | ENSG00000152254 | Tissue enriched | √ |
| BOLL | ENSG00000152430 | Tissue enriched | √ |
| SUV39H2 | ENSG00000152455 | Tissue enriched | √ |
| TRIM36 | ENSG00000152503 | Tissue enriched | √ |
| GRIA4 | ENSG00000152578 | Tissue enriched | √ |
| MEPE | ENSG00000152595 | Tissue enriched | √ |
| CAPSL | ENSG00000152611 | Tissue enriched | √ |
| NADK2 | ENSG00000152620 | Tissue enriched | × |
| DDX4 | ENSG00000152670 | Tissue enriched | √ |
| CATSPER3 | ENSG00000152705 | Tissue enriched | √ |
| SLC16A12 | ENSG00000152779 | Tissue enriched | √ |
| CNTNAP4 | ENSG00000152910 | Tissue enriched | √ |
| NRSN1 | ENSG00000152954 | Tissue enriched | √ |
| CPB1 | ENSG00000153002 | Tissue enriched | √ |
| TEKT5 | ENSG00000153060 | Tissue enriched | √ |
| FEZF2 | ENSG00000153266 | Tissue enriched | √ |
| TEX29 | ENSG00000153495 | Tissue enriched | × |
| SPACA7 | ENSG00000153498 | Tissue enriched | × |
| GOLGA8F | ENSG00000153684 | Tissue enriched | √ |
| TGIF2LX | ENSG00000153779 | Tissue enriched | √ |
| FAM92B | ENSG00000153789 | Tissue enriched | √ |
| C7orf31 | ENSG00000153790 | Tissue enriched | √ |
| ASB17 | ENSG00000154007 | Tissue enriched | √ |
| SLC5A10 | ENSG00000154025 | Tissue enriched | √ |
| AK5 | ENSG00000154027 | Tissue enriched | √ |
| CABYR | ENSG00000154040 | Tissue enriched | √ |
| JPH3 | ENSG00000154118 | Tissue enriched | √ |
| NRGN | ENSG00000154146 | Tissue enriched | √ |
| OBSCN | ENSG00000154358 | Tissue enriched | √ |
| ASZ1 | ENSG00000154438 | Tissue enriched | √ |
| GPR26 | ENSG00000154478 | Tissue enriched | √ |
| CCDC173 | ENSG00000154479 | Tissue enriched | × |
| PSMA8 | ENSG00000154611 | Tissue enriched | √ |
| TMPRSS15 | ENSG00000154646 | Tissue enriched | √ |
| C17orf50 | ENSG00000154768 | Tissue enriched | √ |
| RAB6B | ENSG00000154917 | Tissue enriched | √ |
| 14×Sep | ENSG00000154997 | Tissue enriched | × |
| ODF1 | ENSG00000155087 | Tissue enriched | √ |
| MAGEC1 | ENSG00000155495 | Tissue enriched | √ |
| GRIA1 | ENSG00000155511 | Tissue enriched | √ |
| XAGE2B | ENSG00000155622 | Tissue enriched | × |
| PDZD9 | ENSG00000155714 | Tissue enriched | √ |
| ALS2CR11 | ENSG00000155754 | Tissue enriched | √ |
| CYLC2 | ENSG00000155833 | Tissue enriched | √ |
| FAM154A | ENSG00000155875 | Tissue enriched | √ |
| TRIM42 | ENSG00000155890 | Tissue enriched | √ |
| RAET1L | ENSG00000155918 | Tissue enriched | √ |
| KIF5A | ENSG00000155980 | Tissue enriched | √ |
| MAGEA8 | ENSG00000156009 | Tissue enriched | √ |
| UGT2B4 | ENSG00000156096 | Tissue enriched | √ |
| CLDN17 | ENSG00000156282 | Tissue enriched | √ |
| FBXO43 | ENSG00000156509 | Tissue enriched | √ |
| PRG3 | ENSG00000156575 | Tissue enriched | √ |
| LHFPL4 | ENSG00000156959 | Tissue enriched | √ |
| GHRL | ENSG00000157017 | Tissue enriched | √ |
| SHCBP1L | ENSG00000157060 | Tissue enriched | × |
| LYZL4 | ENSG00000157093 | Tissue enriched | √ |
| KLHL40 | ENSG00000157119 | Tissue enriched | × |
| C8A | ENSG00000157131 | Tissue enriched | √ |
| TIMP4 | ENSG00000157150 | Tissue enriched | √ |
| STEAP2 | ENSG00000157214 | Tissue enriched | √ |
| ARMC12 | ENSG00000157343 | Tissue enriched | × |
| MUM1L1 | ENSG00000157502 | Tissue enriched | √ |
| LCA5L | ENSG00000157578 | Tissue enriched | √ |
| C9orf43 | ENSG00000157653 | Tissue enriched | √ |
| SLC34A2 | ENSG00000157765 | Tissue enriched | √ |
| CABP1 | ENSG00000157782 | Tissue enriched | √ |
| CIB4 | ENSG00000157884 | Tissue enriched | √ |
| GALNT14 | ENSG00000158089 | Tissue enriched | √ |
| SLC13A3 | ENSG00000158296 | Tissue enriched | √ |
| CPA2 | ENSG00000158516 | Tissue enriched | √ |
| CPA5 | ENSG00000158525 | Tissue enriched | √ |
| POM121L2 | ENSG00000158553 | Tissue enriched | √ |
| ALAS2 | ENSG00000158578 | Tissue enriched | √ |
| PAGE5 | ENSG00000158639 | Tissue enriched | √ |
| SLC45A3 | ENSG00000158715 | Tissue enriched | √ |
| APOA2 | ENSG00000158874 | Tissue enriched | √ |
| SV2A | ENSG00000159164 | Tissue enriched | √ |
| TNNI1 | ENSG00000159173 | Tissue enriched | √ |
| KCNE2 | ENSG00000159197 | Tissue enriched | √ |
| ACTC1 | ENSG00000159251 | Tissue enriched | √ |
| GOLGA6A | ENSG00000159289 | Tissue enriched | √ |
| PADI4 | ENSG00000159339 | Tissue enriched | √ |
| LCE2B | ENSG00000159455 | Tissue enriched | √ |
| RGL4 | ENSG00000159496 | Tissue enriched | √ |
| SPRR2G | ENSG00000159516 | Tissue enriched | √ |
| CCDC17 | ENSG00000159588 | Tissue enriched | √ |
| UROC1 | ENSG00000159650 | Tissue enriched | √ |
| PIP | ENSG00000159763 | Tissue enriched | √ |
| FAM131B | ENSG00000159784 | Tissue enriched | √ |
| ANKLE1 | ENSG00000160117 | Tissue enriched | √ |
| TFF1 | ENSG00000160182 | Tissue enriched | √ |
| RSPH1 | ENSG00000160188 | Tissue enriched | √ |
| CRYAA | ENSG00000160202 | Tissue enriched | √ |
| HSF2BP | ENSG00000160207 | Tissue enriched | √ |
| FCN2 | ENSG00000160339 | Tissue enriched | √ |
| LCN1 | ENSG00000160349 | Tissue enriched | √ |
| HIPK4 | ENSG00000160396 | Tissue enriched | √ |
| C9orf117 | ENSG00000160401 | Tissue enriched | √ |
| BRSK1 | ENSG00000160469 | Tissue enriched | √ |
| COX6B2 | ENSG00000160471 | Tissue enriched | √ |
| TMEM190 | ENSG00000160472 | Tissue enriched | √ |
| CHRNB2 | ENSG00000160716 | Tissue enriched | √ |
| PAQR6 | ENSG00000160781 | Tissue enriched | √ |
| PTH1R | ENSG00000160801 | Tissue enriched | √ |
| CYP11B1 | ENSG00000160882 | Tissue enriched | √ |
| CCDC105 | ENSG00000160994 | Tissue enriched | √ |
| PGLYRP2 | ENSG00000161031 | Tissue enriched | √ |
| LRWD1 | ENSG00000161036 | Tissue enriched | √ |
| CELF5 | ENSG00000161082 | Tissue enriched | √ |
| AC008132.13 | ENSG00000161103 | Tissue enriched | × |
| CCDC116 | ENSG00000161180 | Tissue enriched | √ |
| DMKN | ENSG00000161249 | Tissue enriched | √ |
| LYZL6 | ENSG00000161572 | Tissue enriched | √ |
| CCL16 | ENSG00000161573 | Tissue enriched | √ |
| KLHL10 | ENSG00000161594 | Tissue enriched | √ |
| CCDC155 | ENSG00000161609 | Tissue enriched | √ |
| DCD | ENSG00000161634 | Tissue enriched | √ |
| IZUMO2 | ENSG00000161652 | Tissue enriched | × |
| ASB16 | ENSG00000161664 | Tissue enriched | √ |
| SYCE2 | ENSG00000161860 | Tissue enriched | √ |
| ASGR2 | ENSG00000161944 | Tissue enriched | √ |
| CCDC42 | ENSG00000161973 | Tissue enriched | √ |
| C16orf11 | ENSG00000161992 | Tissue enriched | √ |
| CCDC78 | ENSG00000162004 | Tissue enriched | √ |
| MEIOB | ENSG00000162039 | Tissue enriched | × |
| HS3ST6 | ENSG00000162040 | Tissue enriched | √ |
| ZG16B | ENSG00000162078 | Tissue enriched | √ |
| CLPB | ENSG00000162129 | Tissue enriched | √ |
| GNG3 | ENSG00000162188 | Tissue enriched | √ |
| ITIH3 | ENSG00000162267 | Tissue enriched | √ |
| FGF19 | ENSG00000162344 | Tissue enriched | √ |
| CTRC | ENSG00000162438 | Tissue enriched | √ |
| CCDC27 | ENSG00000162592 | Tissue enriched | √ |
| LRRIQ3 | ENSG00000162620 | Tissue enriched | √ |
| HENMT1 | ENSG00000162639 | Tissue enriched | × |
| AGL | ENSG00000162688 | Tissue enriched | √ |
| KCNJ9 | ENSG00000162728 | Tissue enriched | √ |
| SLC9C2 | ENSG00000162753 | Tissue enriched | × |
| LRRC52 | ENSG00000162763 | Tissue enriched | √ |
| FAM71A | ENSG00000162771 | Tissue enriched | √ |
| TDRD5 | ENSG00000162782 | Tissue enriched | √ |
| WDR64 | ENSG00000162843 | Tissue enriched | √ |
| KLHDC8A | ENSG00000162873 | Tissue enriched | √ |
| PM20D1 | ENSG00000162877 | Tissue enriched | √ |
| KCNF1 | ENSG00000162975 | Tissue enriched | √ |
| ZSWIM2 | ENSG00000163012 | Tissue enriched | √ |
| ANKRD30BL | ENSG00000163046 | Tissue enriched | × |
| ADCK3 | ENSG00000163050 | Tissue enriched | × |
| BIRC8 | ENSG00000163098 | Tissue enriched | √ |
| PDHA2 | ENSG00000163114 | Tissue enriched | √ |
| ANKRD23 | ENSG00000163126 | Tissue enriched | √ |
| TMOD4 | ENSG00000163157 | Tissue enriched | √ |
| SMCP | ENSG00000163206 | Tissue enriched | √ |
| BMP10 | ENSG00000163217 | Tissue enriched | √ |
| S100A12 | ENSG00000163221 | Tissue enriched | √ |
| TDRD10 | ENSG00000163239 | Tissue enriched | √ |
| CRYGC | ENSG00000163254 | Tissue enriched | √ |
| ALPP | ENSG00000163283 | Tissue enriched | √ |
| GABRG1 | ENSG00000163285 | Tissue enriched | √ |
| FAM19A4 | ENSG00000163377 | Tissue enriched | √ |
| C3orf30 | ENSG00000163424 | Tissue enriched | √ |
| PDCL2 | ENSG00000163440 | Tissue enriched | √ |
| TSACC | ENSG00000163467 | Tissue enriched | × |
| CCDC141 | ENSG00000163492 | Tissue enriched | √ |
| DPPA2 | ENSG00000163530 | Tissue enriched | √ |
| SERPINI1 | ENSG00000163536 | Tissue enriched | √ |
| SPTA1 | ENSG00000163554 | Tissue enriched | √ |
| SLC2A2 | ENSG00000163581 | Tissue enriched | √ |
| ALB | ENSG00000163631 | Tissue enriched | √ |
| FAM194A | ENSG00000163645 | Tissue enriched | √ |
| CCNL1 | ENSG00000163660 | Tissue enriched | √ |
| FANCD2OS | ENSG00000163705 | Tissue enriched | × |
| PPBP | ENSG00000163736 | Tissue enriched | √ |
| PF4 | ENSG00000163737 | Tissue enriched | √ |
| PLSCR2 | ENSG00000163746 | Tissue enriched | √ |
| CCDC158 | ENSG00000163749 | Tissue enriched | √ |
| DNAJC5G | ENSG00000163793 | Tissue enriched | √ |
| SPDYA | ENSG00000163806 | Tissue enriched | √ |
| TGM4 | ENSG00000163810 | Tissue enriched | √ |
| RTP3 | ENSG00000163825 | Tissue enriched | √ |
| GRIK3 | ENSG00000163873 | Tissue enriched | √ |
| RPL39L | ENSG00000163923 | Tissue enriched | √ |
| SFMBT1 | ENSG00000163935 | Tissue enriched | √ |
| ZDHHC19 | ENSG00000163958 | Tissue enriched | √ |
| MFI2 | ENSG00000163975 | Tissue enriched | √ |
| SLC9B1 | ENSG00000164037 | Tissue enriched | × |
| CAMP | ENSG00000164047 | Tissue enriched | √ |
| FBXW12 | ENSG00000164049 | Tissue enriched | √ |
| HSPA4L | ENSG00000164070 | Tissue enriched | √ |
| CAMKV | ENSG00000164076 | Tissue enriched | √ |
| ADAD1 | ENSG00000164113 | Tissue enriched | √ |
| C4orf45 | ENSG00000164123 | Tissue enriched | √ |
| PRDM9 | ENSG00000164256 | Tissue enriched | √ |
| SCGB3A2 | ENSG00000164265 | Tissue enriched | √ |
| SPINK1 | ENSG00000164266 | Tissue enriched | √ |
| SPZ1 | ENSG00000164299 | Tissue enriched | √ |
| CAGE1 | ENSG00000164304 | Tissue enriched | √ |
| TMEM174 | ENSG00000164325 | Tissue enriched | √ |
| CARTPT | ENSG00000164326 | Tissue enriched | √ |
| FAM170A | ENSG00000164334 | Tissue enriched | √ |
| KLKB1 | ENSG00000164344 | Tissue enriched | √ |
| SLC6A18 | ENSG00000164363 | Tissue enriched | √ |
| GDF9 | ENSG00000164404 | Tissue enriched | √ |
| LEAP2 | ENSG00000164406 | Tissue enriched | √ |
| FABP7 | ENSG00000164434 | Tissue enriched | √ |
| FAM26D | ENSG00000164451 | Tissue enriched | √ |
| C7orf72 | ENSG00000164500 | Tissue enriched | √ |
| HIST1H2AA | ENSG00000164508 | Tissue enriched | √ |
| TBX20 | ENSG00000164532 | Tissue enriched | √ |
| FAM183B | ENSG00000164556 | Tissue enriched | √ |
| MYOZ3 | ENSG00000164591 | Tissue enriched | √ |
| NEUROD6 | ENSG00000164600 | Tissue enriched | √ |
| SLC29A4 | ENSG00000164638 | Tissue enriched | √ |
| C7orf62 | ENSG00000164645 | Tissue enriched | × |
| SP8 | ENSG00000164651 | Tissue enriched | √ |
| IQUB | ENSG00000164675 | Tissue enriched | √ |
| SLC13A4 | ENSG00000164707 | Tissue enriched | √ |
| SLC35G3 | ENSG00000164729 | Tissue enriched | × |
| ADCY1 | ENSG00000164742 | Tissue enriched | √ |
| SUN3 | ENSG00000164744 | Tissue enriched | √ |
| SLC30A8 | ENSG00000164756 | Tissue enriched | √ |
| PHKG1 | ENSG00000164776 | Tissue enriched | √ |
| KCNV1 | ENSG00000164794 | Tissue enriched | √ |
| DEFA4 | ENSG00000164821 | Tissue enriched | √ |
| NOS3 | ENSG00000164867 | Tissue enriched | √ |
| CA3 | ENSG00000164879 | Tissue enriched | √ |
| SLC7A13 | ENSG00000164893 | Tissue enriched | √ |
| BAALC | ENSG00000164929 | Tissue enriched | √ |
| DIRAS2 | ENSG00000165023 | Tissue enriched | √ |
| LETM2 | ENSG00000165046 | Tissue enriched | √ |
| PRKACG | ENSG00000165059 | Tissue enriched | √ |
| ZMAT4 | ENSG00000165061 | Tissue enriched | √ |
| PRSS37 | ENSG00000165076 | Tissue enriched | √ |
| C8orf34 | ENSG00000165084 | Tissue enriched | √ |
| GKAP1 | ENSG00000165113 | Tissue enriched | √ |
| SSMEM1 | ENSG00000165120 | Tissue enriched | × |
| C7orf34 | ENSG00000165131 | Tissue enriched | √ |
| CXorf22 | ENSG00000165164 | Tissue enriched | √ |
| C9orf84 | ENSG00000165181 | Tissue enriched | √ |
| CXorf58 | ENSG00000165182 | Tissue enriched | √ |
| TSHR | ENSG00000165409 | Tissue enriched | √ |
| PGM2L1 | ENSG00000165434 | Tissue enriched | √ |
| PHOX2A | ENSG00000165462 | Tissue enriched | √ |
| MBL2 | ENSG00000165471 | Tissue enriched | √ |
| GJB2 | ENSG00000165474 | Tissue enriched | √ |
| RPL10L | ENSG00000165496 | Tissue enriched | √ |
| NOXRED1 | ENSG00000165555 | Tissue enriched | × |
| AMER2 | ENSG00000165566 | Tissue enriched | × |
| AKR1E2 | ENSG00000165568 | Tissue enriched | √ |
| SSX3 | ENSG00000165584 | Tissue enriched | √ |
| DRGX | ENSG00000165606 | Tissue enriched | √ |
| CLEC1B | ENSG00000165682 | Tissue enriched | √ |
| TMEM52B | ENSG00000165685 | Tissue enriched | × |
| GFI1B | ENSG00000165702 | Tissue enriched | √ |
| RET | ENSG00000165731 | Tissue enriched | √ |
| C12orf50 | ENSG00000165805 | Tissue enriched | √ |
| C10orf82 | ENSG00000165863 | Tissue enriched | √ |
| RAPSN | ENSG00000165917 | Tissue enriched | √ |
| SERPINA12 | ENSG00000165953 | Tissue enriched | √ |
| SLC6A5 | ENSG00000165970 | Tissue enriched | √ |
| CCDC38 | ENSG00000165972 | Tissue enriched | √ |
| C1QL3 | ENSG00000165985 | Tissue enriched | √ |
| KCNC2 | ENSG00000166006 | Tissue enriched | √ |
| MAGEA9 | ENSG00000166008 | Tissue enriched | × |
| LIPC | ENSG00000166035 | Tissue enriched | √ |
| PASD1 | ENSG00000166049 | Tissue enriched | √ |
| TMCO5A | ENSG00000166069 | Tissue enriched | √ |
| CMTM5 | ENSG00000166091 | Tissue enriched | √ |
| SVOP | ENSG00000166111 | Tissue enriched | √ |
| SPATA19 | ENSG00000166118 | Tissue enriched | √ |
| C16orf78 | ENSG00000166152 | Tissue enriched | √ |
| TPTE | ENSG00000166157 | Tissue enriched | √ |
| LRTM2 | ENSG00000166159 | Tissue enriched | √ |
| SPIC | ENSG00000166211 | Tissue enriched | √ |
| TBATA | ENSG00000166220 | Tissue enriched | × |
| SCN3B | ENSG00000166257 | Tissue enriched | √ |
| C11orf65 | ENSG00000166323 | Tissue enriched | √ |
| CCDC182 | ENSG00000166329 | Tissue enriched | × |
| NETO1 | ENSG00000166342 | Tissue enriched | √ |
| MSS51 | ENSG00000166343 | Tissue enriched | × |
| POTED | ENSG00000166351 | Tissue enriched | √ |
| WDR88 | ENSG00000166359 | Tissue enriched | √ |
| A2ML1 | ENSG00000166535 | Tissue enriched | √ |
| CDH16 | ENSG00000166589 | Tissue enriched | √ |
| WDR16 | ENSG00000166596 | Tissue enriched | √ |
| MMP10 | ENSG00000166670 | Tissue enriched | √ |
| LDHC | ENSG00000166796 | Tissue enriched | √ |
| LDHAL6A | ENSG00000166800 | Tissue enriched | √ |
| PLIN1 | ENSG00000166819 | Tissue enriched | √ |
| CACNG2 | ENSG00000166862 | Tissue enriched | √ |
| TAC3 | ENSG00000166863 | Tissue enriched | √ |
| ELFN2 | ENSG00000166897 | Tissue enriched | √ |
| MS4A6E | ENSG00000166926 | Tissue enriched | √ |
| MS4A14 | ENSG00000166928 | Tissue enriched | √ |
| MS4A5 | ENSG00000166930 | Tissue enriched | √ |
| EPB42 | ENSG00000166947 | Tissue enriched | √ |
| MS4A15 | ENSG00000166961 | Tissue enriched | √ |
| MAP1A | ENSG00000166963 | Tissue enriched | √ |
| TCP10L2 | ENSG00000166984 | Tissue enriched | √ |
| C15orf43 | ENSG00000167014 | Tissue enriched | √ |
| NKX3×1 | ENSG00000167034 | Tissue enriched | √ |
| SUN5 | ENSG00000167098 | Tissue enriched | √ |
| TBC1D21 | ENSG00000167139 | Tissue enriched | √ |
| C16orf92 | ENSG00000167194 | Tissue enriched | √ |
| GOLGA6C | ENSG00000167195 | Tissue enriched | √ |
| LOXHD1 | ENSG00000167210 | Tissue enriched | √ |
| IGF2 | ENSG00000167244 | Tissue enriched | √ |
| OR51E2 | ENSG00000167332 | Tissue enriched | √ |
| MMP26 | ENSG00000167346 | Tissue enriched | √ |
| LPO | ENSG00000167419 | Tissue enriched | √ |
| JSRP1 | ENSG00000167476 | Tissue enriched | √ |
| CDT1 | ENSG00000167513 | Tissue enriched | √ |
| PROCA1 | ENSG00000167525 | Tissue enriched | √ |
| LALBA | ENSG00000167531 | Tissue enriched | √ |
| AQP2 | ENSG00000167580 | Tissue enriched | √ |
| ANKRD33 | ENSG00000167612 | Tissue enriched | √ |
| ATCAY | ENSG00000167654 | Tissue enriched | √ |
| PLIN4 | ENSG00000167676 | Tissue enriched | √ |
| SERPINF2 | ENSG00000167711 | Tissue enriched | √ |
| KLK4 | ENSG00000167749 | Tissue enriched | √ |
| KLK2 | ENSG00000167751 | Tissue enriched | √ |
| KLK5 | ENSG00000167754 | Tissue enriched | √ |
| KLK13 | ENSG00000167759 | Tissue enriched | √ |
| KRT1 | ENSG00000167768 | Tissue enriched | √ |
| MGAT5B | ENSG00000167889 | Tissue enriched | √ |
| CYP7A1 | ENSG00000167910 | Tissue enriched | √ |
| GSDMA | ENSG00000167914 | Tissue enriched | √ |
| SOST | ENSG00000167941 | Tissue enriched | √ |
| DNASE1L2 | ENSG00000167968 | Tissue enriched | √ |
| CASKIN1 | ENSG00000167971 | Tissue enriched | √ |
| C11orf85 | ENSG00000168070 | Tissue enriched | √ |
| HIST3H3 | ENSG00000168148 | Tissue enriched | √ |
| KCNV2 | ENSG00000168263 | Tissue enriched | √ |
| PTF1A | ENSG00000168267 | Tissue enriched | √ |
| HIST1H1E | ENSG00000168298 | Tissue enriched | √ |
| MOBP | ENSG00000168314 | Tissue enriched | √ |
| C8orf22 | ENSG00000168333 | Tissue enriched | √ |
| HR | ENSG00000168453 | Tissue enriched | √ |
| TXNDC2 | ENSG00000168454 | Tissue enriched | √ |
| SFTPC | ENSG00000168484 | Tissue enriched | √ |
| CCDC110 | ENSG00000168491 | Tissue enriched | √ |
| MYL1 | ENSG00000168530 | Tissue enriched | √ |
| ADAM29 | ENSG00000168594 | Tissue enriched | √ |
| ADAM18 | ENSG00000168619 | Tissue enriched | √ |
| DPCR1 | ENSG00000168631 | Tissue enriched | √ |
| KCTD19 | ENSG00000168676 | Tissue enriched | √ |
| WFDC12 | ENSG00000168703 | Tissue enriched | √ |
| C20orf62 | ENSG00000168746 | Tissue enriched | × |
| TSPY2 | ENSG00000168757 | Tissue enriched | √ |
| SFTPB | ENSG00000168878 | Tissue enriched | √ |
| ENHO | ENSG00000168913 | Tissue enriched | √ |
| CTRB1 | ENSG00000168925 | Tissue enriched | √ |
| CTRB2 | ENSG00000168928 | Tissue enriched | √ |
| GRM5 | ENSG00000168959 | Tissue enriched | √ |
| CPLX1 | ENSG00000168993 | Tissue enriched | √ |
| NTSR2 | ENSG00000169006 | Tissue enriched | √ |
| VCX3A | ENSG00000169059 | Tissue enriched | √ |
| C8orf46 | ENSG00000169085 | Tissue enriched | √ |
| GOT1L1 | ENSG00000169154 | Tissue enriched | √ |
| MN1 | ENSG00000169184 | Tissue enriched | √ |
| IL13 | ENSG00000169194 | Tissue enriched | √ |
| IL1RAPL1 | ENSG00000169306 | Tissue enriched | √ |
| P2RY12 | ENSG00000169313 | Tissue enriched | √ |
| C22orf15 | ENSG00000169314 | Tissue enriched | √ |
| UMOD | ENSG00000169344 | Tissue enriched | √ |
| GP2 | ENSG00000169347 | Tissue enriched | √ |
| RNASE2 | ENSG00000169385 | Tissue enriched | √ |
| RNASE3 | ENSG00000169397 | Tissue enriched | √ |
| IL8 | ENSG00000169429 | Tissue enriched | √ |
| HTRA4 | ENSG00000169495 | Tissue enriched | √ |
| CT55 | ENSG00000169551 | Tissue enriched | × |
| VPREB1 | ENSG00000169575 | Tissue enriched | √ |
| GKN1 | ENSG00000169605 | Tissue enriched | √ |
| ACTRT2 | ENSG00000169717 | Tissue enriched | √ |
| TAS2R1 | ENSG00000169777 | Tissue enriched | √ |
| LINGO1 | ENSG00000169783 | Tissue enriched | √ |
| RBMY1F | ENSG00000169800 | Tissue enriched | √ |
| CTNND2 | ENSG00000169862 | Tissue enriched | √ |
| AHSP | ENSG00000169877 | Tissue enriched | √ |
| CALML6 | ENSG00000169885 | Tissue enriched | √ |
| PYDC1 | ENSG00000169900 | Tissue enriched | √ |
| S100G | ENSG00000169906 | Tissue enriched | √ |
| FRMPD4 | ENSG00000169933 | Tissue enriched | √ |
| HSFY2 | ENSG00000169953 | Tissue enriched | √ |
| GPR37L1 | ENSG00000170075 | Tissue enriched | √ |
| SERPINA6 | ENSG00000170099 | Tissue enriched | √ |
| GPR25 | ENSG00000170128 | Tissue enriched | √ |
| VGLL2 | ENSG00000170162 | Tissue enriched | √ |
| GYPA | ENSG00000170180 | Tissue enriched | √ |
| FABP6 | ENSG00000170231 | Tissue enriched | √ |
| USP50 | ENSG00000170236 | Tissue enriched | √ |
| SLN | ENSG00000170290 | Tissue enriched | √ |
| FABP4 | ENSG00000170323 | Tissue enriched | √ |
| CST5 | ENSG00000170367 | Tissue enriched | √ |
| CST2 | ENSG00000170369 | Tissue enriched | √ |
| CST1 | ENSG00000170373 | Tissue enriched | √ |
| KRT78 | ENSG00000170423 | Tissue enriched | √ |
| DNAJC18 | ENSG00000170464 | Tissue enriched | √ |
| KRT6C | ENSG00000170465 | Tissue enriched | √ |
| SPATA24 | ENSG00000170469 | Tissue enriched | √ |
| KRT4 | ENSG00000170477 | Tissue enriched | √ |
| KISS1 | ENSG00000170498 | Tissue enriched | √ |
| HSD17B13 | ENSG00000170509 | Tissue enriched | √ |
| COX7B2 | ENSG00000170516 | Tissue enriched | √ |
| KRT83 | ENSG00000170523 | Tissue enriched | √ |
| DLGAP1 | ENSG00000170579 | Tissue enriched | √ |
| FAM71B | ENSG00000170613 | Tissue enriched | √ |
| SCRT1 | ENSG00000170616 | Tissue enriched | √ |
| GTSF1 | ENSG00000170627 | Tissue enriched | √ |
| RBMXL2 | ENSG00000170748 | Tissue enriched | √ |
| TPD52L3 | ENSG00000170777 | Tissue enriched | √ |
| DYDC1 | ENSG00000170788 | Tissue enriched | √ |
| CEL | ENSG00000170835 | Tissue enriched | √ |
| PSG6 | ENSG00000170848 | Tissue enriched | √ |
| PLA2G1B | ENSG00000170890 | Tissue enriched | √ |
| TRH | ENSG00000170893 | Tissue enriched | √ |
| PGK2 | ENSG00000170950 | Tissue enriched | √ |
| PLAC1 | ENSG00000170965 | Tissue enriched | √ |
| DDI1 | ENSG00000170967 | Tissue enriched | √ |
| C8orf74 | ENSG00000171060 | Tissue enriched | √ |
| MUC7 | ENSG00000171195 | Tissue enriched | √ |
| PROL1 | ENSG00000171199 | Tissue enriched | √ |
| SMR3B | ENSG00000171201 | Tissue enriched | √ |
| CSN3 | ENSG00000171209 | Tissue enriched | √ |
| LRG1 | ENSG00000171236 | Tissue enriched | √ |
| NPTX1 | ENSG00000171246 | Tissue enriched | √ |
| CLCN5 | ENSG00000171365 | Tissue enriched | √ |
| TPPP | ENSG00000171368 | Tissue enriched | √ |
| KRT13 | ENSG00000171401 | Tissue enriched | √ |
| XAGE3 | ENSG00000171402 | Tissue enriched | √ |
| XAGE5 | ENSG00000171405 | Tissue enriched | √ |
| CDK5R2 | ENSG00000171450 | Tissue enriched | √ |
| SPACA5B | ENSG00000171478 | Tissue enriched | × |
| SPACA5 | ENSG00000171489 | Tissue enriched | √ |
| MROH2B | ENSG00000171495 | Tissue enriched | × |
| NEUROD2 | ENSG00000171532 | Tissue enriched | √ |
| ECEL1 | ENSG00000171551 | Tissue enriched | √ |
| FGG | ENSG00000171557 | Tissue enriched | √ |
| FGA | ENSG00000171560 | Tissue enriched | √ |
| FGB | ENSG00000171564 | Tissue enriched | √ |
| DSCAM | ENSG00000171587 | Tissue enriched | √ |
| ENC1 | ENSG00000171617 | Tissue enriched | √ |
| SYCE1 | ENSG00000171772 | Tissue enriched | √ |
| SLFNL1 | ENSG00000171790 | Tissue enriched | √ |
| WDR87 | ENSG00000171804 | Tissue enriched | √ |
| PRND | ENSG00000171864 | Tissue enriched | √ |
| KLF17 | ENSG00000171872 | Tissue enriched | √ |
| FBXW10 | ENSG00000171931 | Tissue enriched | √ |
| FOXB1 | ENSG00000171956 | Tissue enriched | √ |
| LDHAL6B | ENSG00000171989 | Tissue enriched | √ |
| MAL | ENSG00000172005 | Tissue enriched | √ |
| GAP43 | ENSG00000172020 | Tissue enriched | √ |
| TEX37 | ENSG00000172073 | Tissue enriched | × |
| SLC9C1 | ENSG00000172139 | Tissue enriched | × |
| LCE1D | ENSG00000172155 | Tissue enriched | √ |
| AZU1 | ENSG00000172232 | Tissue enriched | √ |
| C1QTNF4 | ENSG00000172247 | Tissue enriched | √ |
| CDY1 | ENSG00000172288 | Tissue enriched | √ |
| CDY1B | ENSG00000172352 | Tissue enriched | √ |
| ARNT2 | ENSG00000172379 | Tissue enriched | √ |
| MYOZ2 | ENSG00000172399 | Tissue enriched | √ |
| DNAJB7 | ENSG00000172404 | Tissue enriched | √ |
| EFCAB3 | ENSG00000172421 | Tissue enriched | √ |
| TTC36 | ENSG00000172425 | Tissue enriched | √ |
| HSFY1 | ENSG00000172468 | Tissue enriched | × |
| AGXT | ENSG00000172482 | Tissue enriched | √ |
| ACOT12 | ENSG00000172497 | Tissue enriched | √ |
| FAM170B | ENSG00000172538 | Tissue enriched | √ |
| MUCL1 | ENSG00000172551 | Tissue enriched | √ |
| C17orf66 | ENSG00000172653 | Tissue enriched | √ |
| ZFAND4 | ENSG00000172671 | Tissue enriched | × |
| FAM71D | ENSG00000172717 | Tissue enriched | √ |
| KRT2 | ENSG00000172867 | Tissue enriched | √ |
| SLC22A13 | ENSG00000172940 | Tissue enriched | √ |
| ADH6 | ENSG00000172955 | Tissue enriched | √ |
| XKR3 | ENSG00000172967 | Tissue enriched | √ |
| CCDC63 | ENSG00000173093 | Tissue enriched | √ |
| RP11×683L23.1 | ENSG00000173213 | Tissue enriched | × |
| LIPM | ENSG00000173239 | Tissue enriched | √ |
| PLAC8L1 | ENSG00000173261 | Tissue enriched | √ |
| SLC2A14 | ENSG00000173262 | Tissue enriched | √ |
| KCNK7 | ENSG00000173338 | Tissue enriched | √ |
| IQCF1 | ENSG00000173389 | Tissue enriched | √ |
| GLIPR1L1 | ENSG00000173401 | Tissue enriched | √ |
| CCDC36 | ENSG00000173421 | Tissue enriched | √ |
| RNASE8 | ENSG00000173431 | Tissue enriched | √ |
| RNASE11 | ENSG00000173464 | Tissue enriched | √ |
| SPATA3 | ENSG00000173699 | Tissue enriched | √ |
| WFIKKN2 | ENSG00000173714 | Tissue enriched | √ |
| C1orf100 | ENSG00000173728 | Tissue enriched | √ |
| TOPAZ1 | ENSG00000173769 | Tissue enriched | × |
| CNP | ENSG00000173786 | Tissue enriched | √ |
| TDRD12 | ENSG00000173809 | Tissue enriched | √ |
| 10×Mar | ENSG00000173838 | Tissue enriched | × |
| SLCO4C1 | ENSG00000173930 | Tissue enriched | √ |
| PIFO | ENSG00000173947 | Tissue enriched | × |
| SPERT | ENSG00000174015 | Tissue enriched | √ |
| FAM46D | ENSG00000174016 | Tissue enriched | √ |
| C9orf131 | ENSG00000174038 | Tissue enriched | √ |
| SOCS7 | ENSG00000174111 | Tissue enriched | √ |
| ARL13A | ENSG00000174225 | Tissue enriched | √ |
| C20orf166 | ENSG00000174407 | Tissue enriched | √ |
| STARD6 | ENSG00000174448 | Tissue enriched | √ |
| GOLGA6L2 | ENSG00000174450 | Tissue enriched | × |
| VWC2L | ENSG00000174453 | Tissue enriched | √ |
| TTC9B | ENSG00000174521 | Tissue enriched | √ |
| SLC29A2 | ENSG00000174669 | Tissue enriched | √ |
| LEP | ENSG00000174697 | Tissue enriched | √ |
| FGFBP3 | ENSG00000174721 | Tissue enriched | √ |
| PCP2 | ENSG00000174788 | Tissue enriched | √ |
| C4orf26 | ENSG00000174792 | Tissue enriched | √ |
| CNIH2 | ENSG00000174871 | Tissue enriched | √ |
| CA5A | ENSG00000174990 | Tissue enriched | √ |
| SLC22A1 | ENSG00000175003 | Tissue enriched | √ |
| TEX36 | ENSG00000175018 | Tissue enriched | × |
| RAG2 | ENSG00000175097 | Tissue enriched | √ |
| WFDC5 | ENSG00000175121 | Tissue enriched | √ |
| CADM2 | ENSG00000175161 | Tissue enriched | √ |
| INHBC | ENSG00000175189 | Tissue enriched | √ |
| HIGD2B | ENSG00000175202 | Tissue enriched | √ |
| NPPA | ENSG00000175206 | Tissue enriched | √ |
| CATSPER1 | ENSG00000175294 | Tissue enriched | √ |
| CST6 | ENSG00000175315 | Tissue enriched | √ |
| APOF | ENSG00000175336 | Tissue enriched | √ |
| UBQLNL | ENSG00000175518 | Tissue enriched | √ |
| UBQLN3 | ENSG00000175520 | Tissue enriched | √ |
| PNLIP | ENSG00000175535 | Tissue enriched | √ |
| UCP3 | ENSG00000175564 | Tissue enriched | √ |
| PRM1 | ENSG00000175646 | Tissue enriched | √ |
| TEX26 | ENSG00000175664 | Tissue enriched | × |
| RBMXL3 | ENSG00000175718 | Tissue enriched | √ |
| ZNF645 | ENSG00000175809 | Tissue enriched | √ |
| CCDC168 | ENSG00000175820 | Tissue enriched | × |
| CALCB | ENSG00000175868 | Tissue enriched | √ |
| CREG2 | ENSG00000175874 | Tissue enriched | √ |
| WBSCR28 | ENSG00000175877 | Tissue enriched | √ |
| ASCL3 | ENSG00000176009 | Tissue enriched | √ |
| CHDC2 | ENSG00000176034 | Tissue enriched | × |
| ZNF683 | ENSG00000176083 | Tissue enriched | √ |
| AL445665.1 | ENSG00000176134 | Tissue enriched | × |
| HSF5 | ENSG00000176160 | Tissue enriched | √ |
| ENTHD1 | ENSG00000176177 | Tissue enriched | √ |
| CIDEA | ENSG00000176194 | Tissue enriched | √ |
| LRRTM4 | ENSG00000176204 | Tissue enriched | √ |
| ACBD7 | ENSG00000176244 | Tissue enriched | √ |
| HMGB4 | ENSG00000176256 | Tissue enriched | √ |
| OR4N2 | ENSG00000176294 | Tissue enriched | √ |
| FOXR1 | ENSG00000176302 | Tissue enriched | √ |
| PRR18 | ENSG00000176381 | Tissue enriched | √ |
| CNTD1 | ENSG00000176563 | Tissue enriched | √ |
| DCAF4L2 | ENSG00000176566 | Tissue enriched | √ |
| CNBD1 | ENSG00000176571 | Tissue enriched | √ |
| C14orf177 | ENSG00000176605 | Tissue enriched | √ |
| HORMAD2 | ENSG00000176635 | Tissue enriched | √ |
| RNF152 | ENSG00000176641 | Tissue enriched | √ |
| TGIF2LY | ENSG00000176679 | Tissue enriched | √ |
| PFN4 | ENSG00000176732 | Tissue enriched | √ |
| MAGEB6 | ENSG00000176746 | Tissue enriched | √ |
| TCERG1L | ENSG00000176769 | Tissue enriched | √ |
| GRIN1 | ENSG00000176884 | Tissue enriched | √ |
| SOX11 | ENSG00000176887 | Tissue enriched | √ |
| EFCAB5 | ENSG00000176927 | Tissue enriched | √ |
| LY6H | ENSG00000176956 | Tissue enriched | √ |
| FMR1NB | ENSG00000176988 | Tissue enriched | √ |
| C19orf18 | ENSG00000177025 | Tissue enriched | √ |
| ZDHHC22 | ENSG00000177108 | Tissue enriched | √ |
| CETN1 | ENSG00000177143 | Tissue enriched | √ |
| CLVS1 | ENSG00000177182 | Tissue enriched | √ |
| SPACA4 | ENSG00000177202 | Tissue enriched | √ |
| TRIM72 | ENSG00000177238 | Tissue enriched | √ |
| FBXO39 | ENSG00000177294 | Tissue enriched | √ |
| BEND2 | ENSG00000177324 | Tissue enriched | √ |
| UBE2U | ENSG00000177414 | Tissue enriched | √ |
| NIM1K | ENSG00000177453 | Tissue enriched | × |
| RBM44 | ENSG00000177483 | Tissue enriched | √ |
| VCX2 | ENSG00000177504 | Tissue enriched | √ |
| ST8SIA3 | ENSG00000177511 | Tissue enriched | √ |
| FAM187B | ENSG00000177558 | Tissue enriched | √ |
| C12orf54 | ENSG00000177627 | Tissue enriched | √ |
| PNPLA2 | ENSG00000177666 | Tissue enriched | √ |
| C2orf57 | ENSG00000177673 | Tissue enriched | √ |
| MAGEB10 | ENSG00000177689 | Tissue enriched | √ |
| YIPF7 | ENSG00000177752 | Tissue enriched | √ |
| MYOZ1 | ENSG00000177791 | Tissue enriched | √ |
| CAPZA3 | ENSG00000177938 | Tissue enriched | √ |
| ODF3 | ENSG00000177947 | Tissue enriched | √ |
| SPATA31E1 | ENSG00000177992 | Tissue enriched | × |
| TSPYL6 | ENSG00000178021 | Tissue enriched | √ |
| TSSK6 | ENSG00000178093 | Tissue enriched | √ |
| AMER3 | ENSG00000178171 | Tissue enriched | × |
| TMEM151B | ENSG00000178233 | Tissue enriched | √ |
| SLITRK1 | ENSG00000178235 | Tissue enriched | √ |
| PRM3 | ENSG00000178257 | Tissue enriched | √ |
| TNP2 | ENSG00000178279 | Tissue enriched | √ |
| C1orf65 | ENSG00000178395 | Tissue enriched | √ |
| DDC8 | ENSG00000178404 | Tissue enriched | × |
| MCMDC2 | ENSG00000178460 | Tissue enriched | × |
| OTOS | ENSG00000178602 | Tissue enriched | √ |
| C10orf53 | ENSG00000178645 | Tissue enriched | √ |
| DYNAP | ENSG00000178690 | Tissue enriched | × |
| CPN2 | ENSG00000178772 | Tissue enriched | √ |
| H1FOO | ENSG00000178804 | Tissue enriched | √ |
| FAM101A | ENSG00000178882 | Tissue enriched | √ |
| FOXE1 | ENSG00000178919 | Tissue enriched | √ |
| LGALS7B | ENSG00000178934 | Tissue enriched | √ |
| EXD1 | ENSG00000178997 | Tissue enriched | √ |
| C9orf50 | ENSG00000179058 | Tissue enriched | √ |
| C12orf42 | ENSG00000179088 | Tissue enriched | √ |
| CYP11B2 | ENSG00000179142 | Tissue enriched | √ |
| ALOXE3 | ENSG00000179148 | Tissue enriched | √ |
| PXT1 | ENSG00000179165 | Tissue enriched | √ |
| GGN | ENSG00000179168 | Tissue enriched | √ |
| SIGLECL1 | ENSG00000179213 | Tissue enriched | × |
| DAND5 | ENSG00000179284 | Tissue enriched | √ |
| TMEM151A | ENSG00000179292 | Tissue enriched | √ |
| TMEM31 | ENSG00000179363 | Tissue enriched | √ |
| C1orf101 | ENSG00000179397 | Tissue enriched | √ |
| DNAJB8 | ENSG00000179407 | Tissue enriched | √ |
| SLC17A8 | ENSG00000179520 | Tissue enriched | √ |
| RNF151 | ENSG00000179580 | Tissue enriched | √ |
| TPPP2 | ENSG00000179636 | Tissue enriched | √ |
| RPRML | ENSG00000179673 | Tissue enriched | √ |
| KIAA1875 | ENSG00000179698 | Tissue enriched | √ |
| SYCN | ENSG00000179751 | Tissue enriched | √ |
| FAM216B | ENSG00000179813 | Tissue enriched | × |
| MRGPRX3 | ENSG00000179826 | Tissue enriched | √ |
| NKPD1 | ENSG00000179846 | Tissue enriched | √ |
| ABCA13 | ENSG00000179869 | Tissue enriched | √ |
| NRXN1 | ENSG00000179915 | Tissue enriched | √ |
| FAM71E2 | ENSG00000180043 | Tissue enriched | √ |
| WFDC11 | ENSG00000180083 | Tissue enriched | √ |
| TDRD6 | ENSG00000180113 | Tissue enriched | √ |
| C12orf40 | ENSG00000180116 | Tissue enriched | √ |
| CSNK1A1L | ENSG00000180138 | Tissue enriched | √ |
| TH | ENSG00000180176 | Tissue enriched | √ |
| MYLPF | ENSG00000180209 | Tissue enriched | √ |
| F2 | ENSG00000180210 | Tissue enriched | √ |
| FAM71C | ENSG00000180219 | Tissue enriched | √ |
| PRNT | ENSG00000180259 | Tissue enriched | √ |
| C17orf104 | ENSG00000180336 | Tissue enriched | √ |
| DEFB123 | ENSG00000180424 | Tissue enriched | √ |
| C11orf71 | ENSG00000180425 | Tissue enriched | √ |
| CYP8B1 | ENSG00000180432 | Tissue enriched | √ |
| GLIPR1L2 | ENSG00000180481 | Tissue enriched | √ |
| DEFB119 | ENSG00000180483 | Tissue enriched | √ |
| C3orf22 | ENSG00000180697 | Tissue enriched | √ |
| ANKRD30B | ENSG00000180777 | Tissue enriched | √ |
| MAP3K15 | ENSG00000180815 | Tissue enriched | √ |
| C11orf42 | ENSG00000180878 | Tissue enriched | √ |
| GPR62 | ENSG00000180929 | Tissue enriched | √ |
| GPR137C | ENSG00000180998 | Tissue enriched | √ |
| C17orf47 | ENSG00000181013 | Tissue enriched | √ |
| LSMEM1 | ENSG00000181016 | Tissue enriched | × |
| ANKRD34A | ENSG00000181039 | Tissue enriched | √ |
| ADIPOQ | ENSG00000181092 | Tissue enriched | √ |
| C4orf50 | ENSG00000181215 | Tissue enriched | √ |
| SLC25A41 | ENSG00000181240 | Tissue enriched | √ |
| OR5AK2 | ENSG00000181273 | Tissue enriched | √ |
| NME9 | ENSG00000181322 | Tissue enriched | × |
| SPEM1 | ENSG00000181323 | Tissue enriched | √ |
| SAGE1 | ENSG00000181433 | Tissue enriched | √ |
| TMEM45A | ENSG00000181458 | Tissue enriched | √ |
| CDRT1 | ENSG00000181464 | Tissue enriched | √ |
| ODF3L2 | ENSG00000181781 | Tissue enriched | √ |
| ACTL9 | ENSG00000181786 | Tissue enriched | √ |
| FTMT | ENSG00000181867 | Tissue enriched | √ |
| RTKN2 | ENSG00000182010 | Tissue enriched | √ |
| ADIG | ENSG00000182035 | Tissue enriched | √ |
| PTCHD3 | ENSG00000182077 | Tissue enriched | √ |
| FAM181B | ENSG00000182103 | Tissue enriched | √ |
| TEX28P1 | ENSG00000182242 | Tissue enriched | × |
| NLRP10 | ENSG00000182261 | Tissue enriched | √ |
| IZUMO1 | ENSG00000182264 | Tissue enriched | √ |
| DCAF4L1 | ENSG00000182308 | Tissue enriched | √ |
| AC079354.1 | ENSG00000182329 | Tissue enriched | × |
| LIPF | ENSG00000182333 | Tissue enriched | √ |
| CDY2A | ENSG00000182415 | Tissue enriched | × |
| TEX19 | ENSG00000182459 | Tissue enriched | √ |
| SPNS3 | ENSG00000182557 | Tissue enriched | √ |
| VCX | ENSG00000182583 | Tissue enriched | √ |
| ACTL10 | ENSG00000182584 | Tissue enriched | × |
| EPGN | ENSG00000182585 | Tissue enriched | √ |
| HS3ST4 | ENSG00000182601 | Tissue enriched | √ |
| CCDC172 | ENSG00000182645 | Tissue enriched | × |
| PPP1R27 | ENSG00000182676 | Tissue enriched | × |
| PAPPA | ENSG00000182752 | Tissue enriched | √ |
| CCDC87 | ENSG00000182791 | Tissue enriched | √ |
| MAGEB17 | ENSG00000182798 | Tissue enriched | × |
| AC008132.1 | ENSG00000182824 | Tissue enriched | × |
| GPR97 | ENSG00000182885 | Tissue enriched | √ |
| TMEM95 | ENSG00000182896 | Tissue enriched | √ |
| OTOP3 | ENSG00000182938 | Tissue enriched | √ |
| ODF3L1 | ENSG00000182950 | Tissue enriched | √ |
| HMGN4 | ENSG00000182952 | Tissue enriched | √ |
| CYLC1 | ENSG00000183035 | Tissue enriched | √ |
| WBP2NL | ENSG00000183066 | Tissue enriched | √ |
| NEB | ENSG00000183091 | Tissue enriched | √ |
| LYPD4 | ENSG00000183103 | Tissue enriched | √ |
| FAM43B | ENSG00000183114 | Tissue enriched | √ |
| POTEC | ENSG00000183206 | Tissue enriched | √ |
| RIMBP3C | ENSG00000183246 | Tissue enriched | √ |
| PLGLB1 | ENSG00000183281 | Tissue enriched | × |
| AC140481.2 | ENSG00000183292 | Tissue enriched | × |
| FAM9A | ENSG00000183304 | Tissue enriched | √ |
| MAGEA2B | ENSG00000183305 | Tissue enriched | × |
| SPDYE4 | ENSG00000183318 | Tissue enriched | √ |
| C15orf60 | ENSG00000183324 | Tissue enriched | √ |
| GBP6 | ENSG00000183347 | Tissue enriched | √ |
| FHL3 | ENSG00000183386 | Tissue enriched | √ |
| TMEM89 | ENSG00000183396 | Tissue enriched | √ |
| TFDP3 | ENSG00000183434 | Tissue enriched | √ |
| XAGE1C | ENSG00000183461 | Tissue enriched | × |
| TREX2 | ENSG00000183479 | Tissue enriched | √ |
| ACSM5 | ENSG00000183549 | Tissue enriched | √ |
| C10orf120 | ENSG00000183559 | Tissue enriched | √ |
| GKN2 | ENSG00000183607 | Tissue enriched | √ |
| GOLGA8G | ENSG00000183629 | Tissue enriched | √ |
| TP53TG3 | ENSG00000183632 | Tissue enriched | × |
| 11×Mar | ENSG00000183654 | Tissue enriched | × |
| FAM19A1 | ENSG00000183662 | Tissue enriched | √ |
| PSG9 | ENSG00000183668 | Tissue enriched | √ |
| CTAG1A | ENSG00000183678 | Tissue enriched | × |
| BMP8A | ENSG00000183682 | Tissue enriched | √ |
| OR4N4 | ENSG00000183706 | Tissue enriched | √ |
| OPCML | ENSG00000183715 | Tissue enriched | √ |
| BPY2 | ENSG00000183753 | Tissue enriched | √ |
| C9orf66 | ENSG00000183784 | Tissue enriched | √ |
| BPY2B | ENSG00000183795 | Tissue enriched | × |
| SCN5A | ENSG00000183873 | Tissue enriched | √ |
| PP2D1 | ENSG00000183977 | Tissue enriched | × |
| OR2T10 | ENSG00000184022 | Tissue enriched | √ |
| CTAG1B | ENSG00000184033 | Tissue enriched | √ |
| TRIML1 | ENSG00000184108 | Tissue enriched | √ |
| CNTN2 | ENSG00000184144 | Tissue enriched | √ |
| SPRR4 | ENSG00000184148 | Tissue enriched | √ |
| KCNQ3 | ENSG00000184156 | Tissue enriched | √ |
| KCNJ12 | ENSG00000184185 | Tissue enriched | √ |
| OLIG1 | ENSG00000184221 | Tissue enriched | √ |
| PCDH9 | ENSG00000184226 | Tissue enriched | √ |
| KCNK12 | ENSG00000184261 | Tissue enriched | √ |
| MROH7 | ENSG00000184313 | Tissue enriched | × |
| S100A7A | ENSG00000184330 | Tissue enriched | √ |
| IQCF2 | ENSG00000184345 | Tissue enriched | √ |
| SPATA32 | ENSG00000184361 | Tissue enriched | × |
| COLEC10 | ENSG00000184374 | Tissue enriched | √ |
| ACTRT3 | ENSG00000184378 | Tissue enriched | × |
| C21orf128 | ENSG00000184385 | Tissue enriched | √ |
| BPIFC | ENSG00000184459 | Tissue enriched | × |
| FOXO4 | ENSG00000184481 | Tissue enriched | √ |
| POU3F2 | ENSG00000184486 | Tissue enriched | √ |
| GAST | ENSG00000184502 | Tissue enriched | √ |
| NUTM1 | ENSG00000184507 | Tissue enriched | × |
| CEND1 | ENSG00000184524 | Tissue enriched | √ |
| C17orf74 | ENSG00000184560 | Tissue enriched | √ |
| AC132216.1 | ENSG00000184566 | Tissue enriched | × |
| PIWIL3 | ENSG00000184571 | Tissue enriched | √ |
| PRSS55 | ENSG00000184647 | Tissue enriched | √ |
| ODF4 | ENSG00000184650 | Tissue enriched | √ |
| DDX53 | ENSG00000184735 | Tissue enriched | √ |
| MAGEA2 | ENSG00000184750 | Tissue enriched | √ |
| AC013269.5 | ENSG00000184761 | Tissue enriched | × |
| TCTE3 | ENSG00000184786 | Tissue enriched | √ |
| TUSC5 | ENSG00000184811 | Tissue enriched | √ |
| HIST1H2AH | ENSG00000184825 | Tissue enriched | √ |
| H1FX | ENSG00000184897 | Tissue enriched | √ |
| AQP12A | ENSG00000184945 | Tissue enriched | √ |
| SLC22A10 | ENSG00000184999 | Tissue enriched | √ |
| NT5C1B | ENSG00000185013 | Tissue enriched | √ |
| C5orf47 | ENSG00000185056 | Tissue enriched | √ |
| KRT76 | ENSG00000185069 | Tissue enriched | √ |
| ADSSL1 | ENSG00000185100 | Tissue enriched | √ |
| MYADML2 | ENSG00000185105 | Tissue enriched | √ |
| HIST1H2BL | ENSG00000185130 | Tissue enriched | √ |
| AQP12B | ENSG00000185176 | Tissue enriched | √ |
| ZNF479 | ENSG00000185177 | Tissue enriched | √ |
| PRSS57 | ENSG00000185198 | Tissue enriched | × |
| MC2R | ENSG00000185231 | Tissue enriched | √ |
| TEX28 | ENSG00000185254 | Tissue enriched | √ |
| TEX33 | ENSG00000185264 | Tissue enriched | × |
| NOTUM | ENSG00000185269 | Tissue enriched | √ |
| KLHL33 | ENSG00000185271 | Tissue enriched | √ |
| NUPR1L | ENSG00000185290 | Tissue enriched | × |
| SPPL2C | ENSG00000185294 | Tissue enriched | × |
| SFTPA2 | ENSG00000185303 | Tissue enriched | √ |
| OR2V1 | ENSG00000185372 | Tissue enriched | × |
| FAM47A | ENSG00000185448 | Tissue enriched | √ |
| KRT6B | ENSG00000185479 | Tissue enriched | √ |
| STAC3 | ENSG00000185482 | Tissue enriched | √ |
| SV2B | ENSG00000185518 | Tissue enriched | √ |
| C1orf227 | ENSG00000185523 | Tissue enriched | √ |
| NXF2 | ENSG00000185554 | Tissue enriched | √ |
| AHNAK2 | ENSG00000185567 | Tissue enriched | √ |
| SPATA8 | ENSG00000185594 | Tissue enriched | √ |
| PDIA2 | ENSG00000185615 | Tissue enriched | √ |
| KRT79 | ENSG00000185640 | Tissue enriched | √ |
| SMIM23 | ENSG00000185662 | Tissue enriched | × |
| PMEL | ENSG00000185664 | Tissue enriched | × |
| POU3F1 | ENSG00000185668 | Tissue enriched | √ |
| PRAME | ENSG00000185686 | Tissue enriched | √ |
| C6orf201 | ENSG00000185689 | Tissue enriched | √ |
| C11orf87 | ENSG00000185742 | Tissue enriched | √ |
| XAGE2 | ENSG00000185751 | Tissue enriched | √ |
| SPATA31A6 | ENSG00000185775 | Tissue enriched | × |
| NPAP1 | ENSG00000185823 | Tissue enriched | × |
| TMEM210 | ENSG00000185863 | Tissue enriched | × |
| TMPRSS11B | ENSG00000185873 | Tissue enriched | √ |
| PRSS38 | ENSG00000185888 | Tissue enriched | √ |
| BPY2C | ENSG00000185894 | Tissue enriched | × |
| KLHL34 | ENSG00000185915 | Tissue enriched | √ |
| NXF2B | ENSG00000185945 | Tissue enriched | √ |
| C7orf61 | ENSG00000185955 | Tissue enriched | √ |
| FAM186A | ENSG00000185958 | Tissue enriched | √ |
| LCE3A | ENSG00000185962 | Tissue enriched | √ |
| CCIN | ENSG00000185972 | Tissue enriched | √ |
| H2AFB3 | ENSG00000185978 | Tissue enriched | × |
| LEMD1 | ENSG00000186007 | Tissue enriched | √ |
| ATP4B | ENSG00000186009 | Tissue enriched | √ |
| ZPBP2 | ENSG00000186075 | Tissue enriched | √ |
| TEX38 | ENSG00000186118 | Tissue enriched | × |
| C2orf53 | ENSG00000186143 | Tissue enriched | √ |
| DEFB131 | ENSG00000186146 | Tissue enriched | √ |
| UBL4B | ENSG00000186150 | Tissue enriched | √ |
| LCE5A | ENSG00000186207 | Tissue enriched | √ |
| LCE1E | ENSG00000186226 | Tissue enriched | √ |
| AC016757.3 | ENSG00000186235 | Tissue enriched | × |
| KDM4D | ENSG00000186280 | Tissue enriched | √ |
| GABRA5 | ENSG00000186297 | Tissue enriched | √ |
| RGS9BP | ENSG00000186326 | Tissue enriched | √ |
| TMEM212 | ENSG00000186329 | Tissue enriched | √ |
| SLC36A3 | ENSG00000186334 | Tissue enriched | √ |
| KRT10 | ENSG00000186395 | Tissue enriched | √ |
| FCAR | ENSG00000186431 | Tissue enriched | √ |
| KRT3 | ENSG00000186442 | Tissue enriched | √ |
| SPATA12 | ENSG00000186451 | Tissue enriched | √ |
| TMPRSS12 | ENSG00000186452 | Tissue enriched | √ |
| FAM228A | ENSG00000186453 | Tissue enriched | × |
| MYT1L | ENSG00000186487 | Tissue enriched | √ |
| CLCNKA | ENSG00000186510 | Tissue enriched | √ |
| PRG2 | ENSG00000186652 | Tissue enriched | √ |
| CCDC42B | ENSG00000186710 | Tissue enriched | √ |
| CCDC73 | ENSG00000186714 | Tissue enriched | √ |
| LCE1A | ENSG00000186844 | Tissue enriched | √ |
| KRT14 | ENSG00000186847 | Tissue enriched | √ |
| TNFRSF18 | ENSG00000186891 | Tissue enriched | √ |
| SERPINA11 | ENSG00000186910 | Tissue enriched | √ |
| FAM183A | ENSG00000186973 | Tissue enriched | √ |
| ACTL7A | ENSG00000187003 | Tissue enriched | √ |
| RHD | ENSG00000187010 | Tissue enriched | √ |
| PNLIPRP1 | ENSG00000187021 | Tissue enriched | √ |
| TMPRSS6 | ENSG00000187045 | Tissue enriched | √ |
| TMEM262 | ENSG00000187066 | Tissue enriched | × |
| HEATR4 | ENSG00000187105 | Tissue enriched | √ |
| SLIT1 | ENSG00000187122 | Tissue enriched | √ |
| VSTM2B | ENSG00000187135 | Tissue enriched | √ |
| SPATA21 | ENSG00000187144 | Tissue enriched | √ |
| ANGPTL5 | ENSG00000187151 | Tissue enriched | √ |
| H1FNT | ENSG00000187166 | Tissue enriched | √ |
| LCE4A | ENSG00000187170 | Tissue enriched | √ |
| LCE2A | ENSG00000187173 | Tissue enriched | √ |
| LCE2C | ENSG00000187180 | Tissue enriched | √ |
| RP11×195F19.5 | ENSG00000187186 | Tissue enriched | × |
| DAZ3 | ENSG00000187191 | Tissue enriched | √ |
| LCE2D | ENSG00000187223 | Tissue enriched | √ |
| FAM9C | ENSG00000187268 | Tissue enriched | √ |
| CIDEC | ENSG00000187288 | Tissue enriched | √ |
| LHFPL3 | ENSG00000187416 | Tissue enriched | √ |
| KCNJ11 | ENSG00000187486 | Tissue enriched | √ |
| CDHR4 | ENSG00000187492 | Tissue enriched | √ |
| CXorf27 | ENSG00000187516 | Tissue enriched | √ |
| C4orf40 | ENSG00000187533 | Tissue enriched | √ |
| POTEM | ENSG00000187537 | Tissue enriched | × |
| PRAMEF10 | ENSG00000187545 | Tissue enriched | √ |
| SBK2 | ENSG00000187550 | Tissue enriched | √ |
| COX8C | ENSG00000187581 | Tissue enriched | √ |
| TMEM247 | ENSG00000187600 | Tissue enriched | × |
| C5orf52 | ENSG00000187658 | Tissue enriched | √ |
| ERC2 | ENSG00000187672 | Tissue enriched | √ |
| DNAJB13 | ENSG00000187726 | Tissue enriched | √ |
| GABRD | ENSG00000187730 | Tissue enriched | √ |
| C9orf153 | ENSG00000187753 | Tissue enriched | √ |
| ADH1A | ENSG00000187758 | Tissue enriched | √ |
| LIN28B | ENSG00000187772 | Tissue enriched | √ |
| TMEM72 | ENSG00000187783 | Tissue enriched | √ |
| TMEM202 | ENSG00000187806 | Tissue enriched | √ |
| C2orf78 | ENSG00000187833 | Tissue enriched | √ |
| SHISA7 | ENSG00000187902 | Tissue enriched | √ |
| ZCCHC13 | ENSG00000187969 | Tissue enriched | √ |
| C17orf99 | ENSG00000187997 | Tissue enriched | √ |
| C19orf67 | ENSG00000188032 | Tissue enriched | × |
| CLCN1 | ENSG00000188037 | Tissue enriched | √ |
| RNF133 | ENSG00000188050 | Tissue enriched | √ |
| PLA2G4E | ENSG00000188089 | Tissue enriched | √ |
| DAZ1 | ENSG00000188120 | Tissue enriched | √ |
| MAPK12 | ENSG00000188130 | Tissue enriched | √ |
| OTOG | ENSG00000188162 | Tissue enriched | × |
| FAM166A | ENSG00000188163 | Tissue enriched | √ |
| SMTNL2 | ENSG00000188176 | Tissue enriched | √ |
| PRKAR1B | ENSG00000188191 | Tissue enriched | √ |
| FAM230A | ENSG00000188280 | Tissue enriched | × |
| IGFL1 | ENSG00000188293 | Tissue enriched | √ |
| LRRIQ4 | ENSG00000188306 | Tissue enriched | √ |
| CENPP | ENSG00000188312 | Tissue enriched | √ |
| PRR19 | ENSG00000188368 | Tissue enriched | √ |
| PPP3R2 | ENSG00000188386 | Tissue enriched | √ |
| CLEC2A | ENSG00000188393 | Tissue enriched | √ |
| NANOS2 | ENSG00000188425 | Tissue enriched | √ |
| KRTDAP | ENSG00000188508 | Tissue enriched | √ |
| HBA2 | ENSG00000188536 | Tissue enriched | √ |
| NKAIN2 | ENSG00000188580 | Tissue enriched | √ |
| IGFL3 | ENSG00000188624 | Tissue enriched | √ |
| CC2D2B | ENSG00000188649 | Tissue enriched | √ |
| RHCE | ENSG00000188672 | Tissue enriched | √ |
| C2orf80 | ENSG00000188674 | Tissue enriched | √ |
| DUPD1 | ENSG00000188716 | Tissue enriched | √ |
| CATSPER4 | ENSG00000188782 | Tissue enriched | √ |
| TMCO2 | ENSG00000188800 | Tissue enriched | √ |
| HMX2 | ENSG00000188816 | Tissue enriched | √ |
| SNTN | ENSG00000188817 | Tissue enriched | √ |
| C1orf192 | ENSG00000188931 | Tissue enriched | √ |
| LIPI | ENSG00000188992 | Tissue enriched | √ |
| MAGEB16 | ENSG00000189023 | Tissue enriched | √ |
| DUSP21 | ENSG00000189037 | Tissue enriched | √ |
| CGB5 | ENSG00000189052 | Tissue enriched | √ |
| GAGE2A | ENSG00000189064 | Tissue enriched | √ |
| VSTM1 | ENSG00000189068 | Tissue enriched | √ |
| FAM25B | ENSG00000189090 | Tissue enriched | √ |
| FAM47B | ENSG00000189132 | Tissue enriched | √ |
| NKAPL | ENSG00000189134 | Tissue enriched | √ |
| FSCB | ENSG00000189139 | Tissue enriched | √ |
| OR14I1 | ENSG00000189181 | Tissue enriched | √ |
| KRT77 | ENSG00000189182 | Tissue enriched | √ |
| DCAF8L2 | ENSG00000189186 | Tissue enriched | √ |
| SPANXN3 | ENSG00000189252 | Tissue enriched | √ |
| C22orf43 | ENSG00000189269 | Tissue enriched | √ |
| FOXR2 | ENSG00000189299 | Tissue enriched | √ |
| SPANXN4 | ENSG00000189326 | Tissue enriched | √ |
| RP11×113D6.10 | ENSG00000189332 | Tissue enriched | × |
| TBC1D28 | ENSG00000189375 | Tissue enriched | √ |
| OTUD6A | ENSG00000189401 | Tissue enriched | √ |
| GJB4 | ENSG00000189433 | Tissue enriched | √ |
| SYCP2 | ENSG00000196074 | Tissue enriched | √ |
| MYBPC1 | ENSG00000196091 | Tissue enriched | √ |
| VN1R2 | ENSG00000196131 | Tissue enriched | √ |
| SERPINA3 | ENSG00000196136 | Tissue enriched | √ |
| OR10J1 | ENSG00000196184 | Tissue enriched | √ |
| RYR1 | ENSG00000196218 | Tissue enriched | √ |
| SFTA2 | ENSG00000196260 | Tissue enriched | √ |
| ATP2A1 | ENSG00000196296 | Tissue enriched | √ |
| AKR1CL1 | ENSG00000196326 | Tissue enriched | √ |
| STK31 | ENSG00000196335 | Tissue enriched | √ |
| ADH7 | ENSG00000196344 | Tissue enriched | √ |
| ZNF729 | ENSG00000196350 | Tissue enriched | × |
| ELAVL3 | ENSG00000196361 | Tissue enriched | √ |
| FUT4 | ENSG00000196371 | Tissue enriched | √ |
| SLC35F1 | ENSG00000196376 | Tissue enriched | √ |
| INCA1 | ENSG00000196388 | Tissue enriched | √ |
| SPANXD | ENSG00000196406 | Tissue enriched | × |
| THEM5 | ENSG00000196407 | Tissue enriched | √ |
| PRTN3 | ENSG00000196415 | Tissue enriched | √ |
| GK2 | ENSG00000196475 | Tissue enriched | √ |
| HBG2 | ENSG00000196565 | Tissue enriched | √ |
| SLC22A25 | ENSG00000196600 | Tissue enriched | √ |
| RIMBP3 | ENSG00000196622 | Tissue enriched | √ |
| WNK3 | ENSG00000196632 | Tissue enriched | √ |
| LCE1B | ENSG00000196734 | Tissue enriched | √ |
| ANKRD20A1 | ENSG00000196774 | Tissue enriched | × |
| CTB×134H23.2 | ENSG00000196796 | Tissue enriched | × |
| SPRR2B | ENSG00000196805 | Tissue enriched | √ |
| CHRNG | ENSG00000196811 | Tissue enriched | √ |
| ADA | ENSG00000196839 | Tissue enriched | √ |
| TOMM20L | ENSG00000196860 | Tissue enriched | √ |
| C5orf48 | ENSG00000196900 | Tissue enriched | √ |
| RIMBP3B | ENSG00000196934 | Tissue enriched | × |
| ZNF705A | ENSG00000196946 | Tissue enriched | √ |
| NPIPB9 | ENSG00000196993 | Tissue enriched | × |
| LCE1C | ENSG00000197084 | Tissue enriched | √ |
| SLC6A17 | ENSG00000197106 | Tissue enriched | √ |
| ADAM32 | ENSG00000197140 | Tissue enriched | √ |
| MAGEA6 | ENSG00000197172 | Tissue enriched | √ |
| ZSCAN5B | ENSG00000197213 | Tissue enriched | √ |
| SERPINA1 | ENSG00000197249 | Tissue enriched | √ |
| IL27 | ENSG00000197272 | Tissue enriched | √ |
| BLM | ENSG00000197299 | Tissue enriched | √ |
| CYP2B6 | ENSG00000197408 | Tissue enriched | √ |
| GOLGA6L1 | ENSG00000197414 | Tissue enriched | √ |
| OPALIN | ENSG00000197430 | Tissue enriched | √ |
| ELANE | ENSG00000197561 | Tissue enriched | √ |
| MYH6 | ENSG00000197616 | Tissue enriched | √ |
| SERPINB13 | ENSG00000197641 | Tissue enriched | √ |
| CCER1 | ENSG00000197651 | Tissue enriched | × |
| SLC22A24 | ENSG00000197658 | Tissue enriched | √ |
| KRTAP26×1 | ENSG00000197683 | Tissue enriched | √ |
| HSPB9 | ENSG00000197723 | Tissue enriched | √ |
| SCGB1D4 | ENSG00000197745 | Tissue enriched | √ |
| CFD | ENSG00000197766 | Tissue enriched | √ |
| C9orf173 | ENSG00000197768 | Tissue enriched | √ |
| CYP2A13 | ENSG00000197838 | Tissue enriched | √ |
| PRB3 | ENSG00000197870 | Tissue enriched | √ |
| SLC22A12 | ENSG00000197891 | Tissue enriched | √ |
| SLC22A6 | ENSG00000197901 | Tissue enriched | √ |
| FAM25G | ENSG00000197910 | Tissue enriched | × |
| ERO1L | ENSG00000197930 | Tissue enriched | √ |
| AADACL2 | ENSG00000197953 | Tissue enriched | √ |
| MBP | ENSG00000197971 | Tissue enriched | √ |
| SPANXA1 | ENSG00000198021 | Tissue enriched | × |
| ZNF560 | ENSG00000198028 | Tissue enriched | √ |
| TUBA3C | ENSG00000198033 | Tissue enriched | √ |
| ZNF273 | ENSG00000198039 | Tissue enriched | √ |
| CYP2A7 | ENSG00000198077 | Tissue enriched | √ |
| H2AFB1 | ENSG00000198082 | Tissue enriched | √ |
| ADH4 | ENSG00000198099 | Tissue enriched | √ |
| NPIPB6 | ENSG00000198156 | Tissue enriched | × |
| FAM47C | ENSG00000198173 | Tissue enriched | √ |
| CACNA1E | ENSG00000198216 | Tissue enriched | √ |
| ANTXRL | ENSG00000198250 | Tissue enriched | √ |
| H2AFB2 | ENSG00000198307 | Tissue enriched | × |
| TMEM239 | ENSG00000198326 | Tissue enriched | × |
| MYL4 | ENSG00000198336 | Tissue enriched | √ |
| HIST1H2AL | ENSG00000198374 | Tissue enriched | √ |
| TMEM207 | ENSG00000198398 | Tissue enriched | √ |
| CCT8L2 | ENSG00000198445 | Tissue enriched | √ |
| TPM2 | ENSG00000198467 | Tissue enriched | √ |
| ZNF280B | ENSG00000198477 | Tissue enriched | √ |
| KCNRG | ENSG00000198553 | Tissue enriched | √ |
| RD3 | ENSG00000198570 | Tissue enriched | √ |
| SPANXC | ENSG00000198573 | Tissue enriched | √ |
| AKR1C4 | ENSG00000198610 | Tissue enriched | √ |
| RYR2 | ENSG00000198626 | Tissue enriched | √ |
| TAT | ENSG00000198650 | Tissue enriched | √ |
| LPA | ENSG00000198670 | Tissue enriched | √ |
| MAGEA1 | ENSG00000198681 | Tissue enriched | √ |
| GPX6 | ENSG00000198704 | Tissue enriched | √ |
| C19orf45 | ENSG00000198723 | Tissue enriched | √ |
| OXCT2 | ENSG00000198754 | Tissue enriched | √ |
| EGFL6 | ENSG00000198759 | Tissue enriched | √ |
| SYCP1 | ENSG00000198765 | Tissue enriched | √ |
| APCDD1L | ENSG00000198768 | Tissue enriched | √ |
| GRIN3A | ENSG00000198785 | Tissue enriched | √ |
| MAGEB3 | ENSG00000198798 | Tissue enriched | √ |
| LRRC10 | ENSG00000198812 | Tissue enriched | √ |
| GRM3 | ENSG00000198822 | Tissue enriched | √ |
| SELM | ENSG00000198832 | Tissue enriched | √ |
| C1orf68 | ENSG00000198854 | Tissue enriched | √ |
| C9orf96 | ENSG00000198870 | Tissue enriched | √ |
| ASB12 | ENSG00000198881 | Tissue enriched | √ |
| SSX4B | ENSG00000198946 | Tissue enriched | × |
| IBA57×AS1 | ENSG00000203684 | Tissue enriched | × |
| TCP10 | ENSG00000203690 | Tissue enriched | √ |
| CAPN8 | ENSG00000203697 | Tissue enriched | √ |
| C6orf99 | ENSG00000203711 | Tissue enriched | × |
| ECT2L | ENSG00000203734 | Tissue enriched | √ |
| SPRN | ENSG00000203772 | Tissue enriched | √ |
| LOR | ENSG00000203782 | Tissue enriched | √ |
| PRR9 | ENSG00000203783 | Tissue enriched | × |
| LELP1 | ENSG00000203784 | Tissue enriched | √ |
| KPRP | ENSG00000203786 | Tissue enriched | √ |
| FAM24A | ENSG00000203795 | Tissue enriched | √ |
| HSD3B1 | ENSG00000203857 | Tissue enriched | √ |
| HSD3B2 | ENSG00000203859 | Tissue enriched | √ |
| C6orf163 | ENSG00000203872 | Tissue enriched | √ |
| RP11×451M19.3 | ENSG00000203876 | Tissue enriched | × |
| RIPPLY2 | ENSG00000203877 | Tissue enriched | √ |
| OOEP | ENSG00000203907 | Tissue enriched | √ |
| C1orf146 | ENSG00000203910 | Tissue enriched | √ |
| SPANXN2 | ENSG00000203924 | Tissue enriched | √ |
| SPANXA2 | ENSG00000203926 | Tissue enriched | √ |
| LINC00632 | ENSG00000203930 | Tissue enriched | × |
| CXorf66 | ENSG00000203933 | Tissue enriched | √ |
| C10orf62 | ENSG00000203942 | Tissue enriched | √ |
| LDLRAD1 | ENSG00000203985 | Tissue enriched | √ |
| RHOXF2B | ENSG00000203989 | Tissue enriched | √ |
| C1orf185 | ENSG00000204006 | Tissue enriched | √ |
| GLT6D1 | ENSG00000204007 | Tissue enriched | √ |
| IFIT1B | ENSG00000204010 | Tissue enriched | √ |
| CT83 | ENSG00000204019 | Tissue enriched | × |
| LIPN | ENSG00000204020 | Tissue enriched | √ |
| LIPK | ENSG00000204021 | Tissue enriched | √ |
| LIPJ | ENSG00000204022 | Tissue enriched | √ |
| AL359195.1 | ENSG00000204038 | Tissue enriched | × |
| RPA4 | ENSG00000204086 | Tissue enriched | √ |
| CLPSL1 | ENSG00000204140 | Tissue enriched | × |
| GGNBP1 | ENSG00000204188 | Tissue enriched | √ |
| TXNDC8 | ENSG00000204193 | Tissue enriched | √ |
| TMEM235 | ENSG00000204278 | Tissue enriched | × |
| PAGE3 | ENSG00000204279 | Tissue enriched | √ |
| C6orf10 | ENSG00000204296 | Tissue enriched | √ |
| TMEM225 | ENSG00000204300 | Tissue enriched | √ |
| AGER | ENSG00000204305 | Tissue enriched | √ |
| ERICH2 | ENSG00000204334 | Tissue enriched | × |
| BTBD17 | ENSG00000204347 | Tissue enriched | √ |
| SPANXN5 | ENSG00000204363 | Tissue enriched | √ |
| C10orf126 | ENSG00000204365 | Tissue enriched | × |
| XAGE1E | ENSG00000204375 | Tissue enriched | × |
| XAGE1D | ENSG00000204376 | Tissue enriched | √ |
| XAGE1A | ENSG00000204379 | Tissue enriched | × |
| XAGE1B | ENSG00000204382 | Tissue enriched | × |
| HSPA1L | ENSG00000204390 | Tissue enriched | √ |
| CSHL1 | ENSG00000204414 | Tissue enriched | √ |
| LY6G6C | ENSG00000204421 | Tissue enriched | √ |
| LY6G6F | ENSG00000204424 | Tissue enriched | √ |
| FAM155A | ENSG00000204442 | Tissue enriched | √ |
| MCCD1 | ENSG00000204511 | Tissue enriched | √ |
| CCHCR1 | ENSG00000204536 | Tissue enriched | √ |
| PSORS1C2 | ENSG00000204538 | Tissue enriched | √ |
| CDSN | ENSG00000204539 | Tissue enriched | √ |
| MUC21 | ENSG00000204544 | Tissue enriched | √ |
| DEFB121 | ENSG00000204548 | Tissue enriched | √ |
| SSX4 | ENSG00000204645 | Tissue enriched | √ |
| MOG | ENSG00000204655 | Tissue enriched | √ |
| OR2H2 | ENSG00000204657 | Tissue enriched | √ |
| C5orf60 | ENSG00000204661 | Tissue enriched | √ |
| C9orf57 | ENSG00000204669 | Tissue enriched | √ |
| OR2H1 | ENSG00000204688 | Tissue enriched | √ |
| AC025278.1 | ENSG00000204752 | Tissue enriched | × |
| CR769776.1 | ENSG00000204788 | Tissue enriched | × |
| SPATA31A2 | ENSG00000204848 | Tissue enriched | × |
| SPATA31A1 | ENSG00000204849 | Tissue enriched | × |
| FAM221B | ENSG00000204930 | Tissue enriched | × |
| PSG5 | ENSG00000204941 | Tissue enriched | √ |
| LRRC10B | ENSG00000204950 | Tissue enriched | √ |
| FBXO47 | ENSG00000204952 | Tissue enriched | √ |
| PCDHA5 | ENSG00000204965 | Tissue enriched | √ |
| MS4A13 | ENSG00000204979 | Tissue enriched | √ |
| PRSS1 | ENSG00000204983 | Tissue enriched | √ |
| AARD | ENSG00000205002 | Tissue enriched | × |
| LGALS7 | ENSG00000205076 | Tissue enriched | √ |
| CXorf30 | ENSG00000205081 | Tissue enriched | √ |
| TMEM231 | ENSG00000205084 | Tissue enriched | √ |
| FAM71F2 | ENSG00000205085 | Tissue enriched | √ |
| FAM205A | ENSG00000205108 | Tissue enriched | × |
| CDKL4 | ENSG00000205111 | Tissue enriched | √ |
| TMEM88B | ENSG00000205116 | Tissue enriched | √ |
| ARID3C | ENSG00000205143 | Tissue enriched | √ |
| C7orf66 | ENSG00000205174 | Tissue enriched | √ |
| C11orf91 | ENSG00000205177 | Tissue enriched | × |
| CCDC144NL | ENSG00000205212 | Tissue enriched | √ |
| CTXN3 | ENSG00000205279 | Tissue enriched | √ |
| RP11×542P2.1 | ENSG00000205301 | Tissue enriched | × |
| SLCO6A1 | ENSG00000205359 | Tissue enriched | √ |
| KRT6A | ENSG00000205420 | Tissue enriched | √ |
| IZUMO3 | ENSG00000205442 | Tissue enriched | × |
| TP53TG3D | ENSG00000205456 | Tissue enriched | × |
| TP53TG3C | ENSG00000205457 | Tissue enriched | × |
| MFSD2B | ENSG00000205639 | Tissue enriched | √ |
| VCX3B | ENSG00000205642 | Tissue enriched | √ |
| HTN3 | ENSG00000205649 | Tissue enriched | √ |
| TECRL | ENSG00000205678 | Tissue enriched | √ |
| GAGE2E | ENSG00000205775 | Tissue enriched | √ |
| GAGE1 | ENSG00000205777 | Tissue enriched | √ |
| ARRDC5 | ENSG00000205784 | Tissue enriched | √ |
| CYS1 | ENSG00000205795 | Tissue enriched | √ |
| C16orf96 | ENSG00000205832 | Tissue enriched | × |
| GMNC | ENSG00000205835 | Tissue enriched | × |
| RFPL3S | ENSG00000205853 | Tissue enriched | √ |
| C22orf42 | ENSG00000205856 | Tissue enriched | √ |
| LRRC72 | ENSG00000205858 | Tissue enriched | × |
| DEFB134 | ENSG00000205882 | Tissue enriched | √ |
| DAZ4 | ENSG00000205916 | Tissue enriched | √ |
| ONECUT3 | ENSG00000205922 | Tissue enriched | √ |
| OLIG2 | ENSG00000205927 | Tissue enriched | √ |
| DAZ2 | ENSG00000205944 | Tissue enriched | √ |
| SMIM21 | ENSG00000206026 | Tissue enriched | × |
| C18orf63 | ENSG00000206043 | Tissue enriched | × |
| DEFA1 | ENSG00000206047 | Tissue enriched | × |
| KRTAP20×4 | ENSG00000206105 | Tissue enriched | √ |
| RNASE13 | ENSG00000206150 | Tissue enriched | √ |
| HBA1 | ENSG00000206172 | Tissue enriched | √ |
| HBM | ENSG00000206177 | Tissue enriched | √ |
| TCEB3B | ENSG00000206181 | Tissue enriched | √ |
| ANKUB1 | ENSG00000206199 | Tissue enriched | × |
| TSSK2 | ENSG00000206203 | Tissue enriched | × |
| PRR23A | ENSG00000206260 | Tissue enriched | √ |
| LRRC30 | ENSG00000206422 | Tissue enriched | √ |
| CD200R1L | ENSG00000206531 | Tissue enriched | √ |
| PRSS50 | ENSG00000206549 | Tissue enriched | √ |
| TRIM71 | ENSG00000206557 | Tissue enriched | √ |
| XKR4 | ENSG00000206579 | Tissue enriched | √ |
| TSSK1B | ENSG00000212122 | Tissue enriched | √ |
| PRR22 | ENSG00000212123 | Tissue enriched | √ |
| CTAGE1 | ENSG00000212710 | Tissue enriched | √ |
| KRTAP3×2 | ENSG00000212900 | Tissue enriched | √ |
| SYT3 | ENSG00000213023 | Tissue enriched | √ |
| CGB8 | ENSG00000213030 | Tissue enriched | √ |
| C6orf165 | ENSG00000213204 | Tissue enriched | × |
| CSH2 | ENSG00000213218 | Tissue enriched | √ |
| TSGA13 | ENSG00000213265 | Tissue enriched | √ |
| MAGEA12 | ENSG00000213401 | Tissue enriched | √ |
| TTLL13 | ENSG00000213471 | Tissue enriched | √ |
| GBP7 | ENSG00000213512 | Tissue enriched | √ |
| FAM209B | ENSG00000213714 | Tissue enriched | × |
| ATP6V1G2 | ENSG00000213760 | Tissue enriched | √ |
| AC011500.1 | ENSG00000213922 | Tissue enriched | × |
| CCL27 | ENSG00000213927 | Tissue enriched | √ |
| HBG1 | ENSG00000213934 | Tissue enriched | √ |
| ZNF99 | ENSG00000213973 | Tissue enriched | √ |
| MAGEB1 | ENSG00000214107 | Tissue enriched | √ |
| ST7×OT4 | ENSG00000214188 | Tissue enriched | × |
| ANG | ENSG00000214274 | Tissue enriched | √ |
| SOGA3 | ENSG00000214338 | Tissue enriched | × |
| EFCAB9 | ENSG00000214360 | Tissue enriched | × |
| AS3MT | ENSG00000214435 | Tissue enriched | √ |
| C17orf98 | ENSG00000214556 | Tissue enriched | √ |
| TMEM249 | ENSG00000214597 | Tissue enriched | × |
| IQCF5 | ENSG00000214681 | Tissue enriched | √ |
| IQCF6 | ENSG00000214686 | Tissue enriched | √ |
| CAPN14 | ENSG00000214711 | Tissue enriched | √ |
| RP1×139D8.6 | ENSG00000214732 | Tissue enriched | × |
| MS4A18 | ENSG00000214782 | Tissue enriched | × |
| CDRT15L2 | ENSG00000214819 | Tissue enriched | × |
| RAD51AP2 | ENSG00000214842 | Tissue enriched | √ |
| DCDC2C | ENSG00000214866 | Tissue enriched | × |
| AC005082.1 | ENSG00000214871 | Tissue enriched | × |
| SMTNL1 | ENSG00000214872 | Tissue enriched | √ |
| AC003102.1 | ENSG00000214921 | Tissue enriched | × |
| SPATA31D1 | ENSG00000214929 | Tissue enriched | × |
| TBC1D26 | ENSG00000214946 | Tissue enriched | √ |
| C16orf90 | ENSG00000215131 | Tissue enriched | √ |
| MUC5AC | ENSG00000215182 | Tissue enriched | × |
| KCNU1 | ENSG00000215262 | Tissue enriched | √ |
| GAGE12G | ENSG00000215269 | Tissue enriched | × |
| GAGE10 | ENSG00000215274 | Tissue enriched | √ |
| GOLGA6L6 | ENSG00000215405 | Tissue enriched | √ |
| SIAH3 | ENSG00000215475 | Tissue enriched | √ |
| GAB4 | ENSG00000215568 | Tissue enriched | √ |
| CELA2B | ENSG00000215704 | Tissue enriched | √ |
| GAGE12E | ENSG00000216649 | Tissue enriched | × |
| CCDC7 | ENSG00000216937 | Tissue enriched | √ |
| SYCE3 | ENSG00000217442 | Tissue enriched | × |
| TDRD15 | ENSG00000218819 | Tissue enriched | × |
| PAPOLB | ENSG00000218823 | Tissue enriched | √ |
| CTA×299D3.8 | ENSG00000219016 | Tissue enriched | × |
| CELA3B | ENSG00000219073 | Tissue enriched | √ |
| TEX40 | ENSG00000219435 | Tissue enriched | × |
| HTR5A×AS1 | ENSG00000220575 | Tissue enriched | × |
| PSG3 | ENSG00000221826 | Tissue enriched | √ |
| C2orf16 | ENSG00000221843 | Tissue enriched | √ |
| MAGEA3 | ENSG00000221867 | Tissue enriched | √ |
| TMEM257 | ENSG00000221870 | Tissue enriched | × |
| OR3A2 | ENSG00000221882 | Tissue enriched | √ |
| OR1C1 | ENSG00000221888 | Tissue enriched | √ |
| NPTXR | ENSG00000221890 | Tissue enriched | √ |
| POM121L12 | ENSG00000221900 | Tissue enriched | √ |
| FXYD7 | ENSG00000221946 | Tissue enriched | √ |
| MYBPHL | ENSG00000221986 | Tissue enriched | √ |
| C7orf71 | ENSG00000222004 | Tissue enriched | √ |
| LINC01118 | ENSG00000222005 | Tissue enriched | × |
| POTEG | ENSG00000222036 | Tissue enriched | √ |
| DCDC2B | ENSG00000222046 | Tissue enriched | √ |
| CDRT15 | ENSG00000223510 | Tissue enriched | √ |
| EBLN1 | ENSG00000223601 | Tissue enriched | × |
| HBD | ENSG00000223609 | Tissue enriched | √ |
| AC007557.1 | ENSG00000223874 | Tissue enriched | × |
| CT47A10 | ENSG00000224089 | Tissue enriched | √ |
| C17orf72 | ENSG00000224383 | Tissue enriched | √ |
| GPX5 | ENSG00000224586 | Tissue enriched | √ |
| GAGE12J | ENSG00000224659 | Tissue enriched | √ |
| ZNF812 | ENSG00000224689 | Tissue enriched | × |
| GAGE12H | ENSG00000224902 | Tissue enriched | × |
| APOC4×APOC2 | ENSG00000224916 | Tissue enriched | × |
| CT62 | ENSG00000225362 | Tissue enriched | √ |
| RP11×3B7.1 | ENSG00000225399 | Tissue enriched | × |
| TEX22 | ENSG00000226174 | Tissue enriched | × |
| AC104809.3 | ENSG00000226321 | Tissue enriched | × |
| AC102948.2 | ENSG00000226364 | Tissue enriched | × |
| DCAF8L1 | ENSG00000226372 | Tissue enriched | √ |
| CT47A9 | ENSG00000226600 | Tissue enriched | √ |
| KIF4B | ENSG00000226650 | Tissue enriched | √ |
| CT47A12 | ENSG00000226685 | Tissue enriched | × |
| AC079341.1 | ENSG00000226757 | Tissue enriched | × |
| SRRM5 | ENSG00000226763 | Tissue enriched | √ |
| CT45A6 | ENSG00000226907 | Tissue enriched | √ |
| CT47A11 | ENSG00000226929 | Tissue enriched | √ |
| RBMY1J | ENSG00000226941 | Tissue enriched | √ |
| C17orf112 | ENSG00000227011 | Tissue enriched | × |
| ANHX | ENSG00000227059 | Tissue enriched | × |
| SPANXB2 | ENSG00000227234 | Tissue enriched | √ |
| GAGE12D | ENSG00000227488 | Tissue enriched | √ |
| RD3L | ENSG00000227729 | Tissue enriched | × |
| C1orf234 | ENSG00000227868 | Tissue enriched | × |
| CTD×2330K9.3 | ENSG00000228008 | Tissue enriched | × |
| BOD1L2 | ENSG00000228075 | Tissue enriched | × |
| AC136604.1 | ENSG00000228259 | Tissue enriched | × |
| ORM2 | ENSG00000228278 | Tissue enriched | √ |
| PROB1 | ENSG00000228672 | Tissue enriched | × |
| CT45A4 | ENSG00000228836 | Tissue enriched | √ |
| TSPY3 | ENSG00000228927 | Tissue enriched | √ |
| C21orf54 | ENSG00000229086 | Tissue enriched | √ |
| PGA4 | ENSG00000229183 | Tissue enriched | √ |
| ORM1 | ENSG00000229314 | Tissue enriched | √ |
| TSPY8 | ENSG00000229549 | Tissue enriched | × |
| PGA3 | ENSG00000229859 | Tissue enriched | √ |
| PRPS1L1 | ENSG00000229937 | Tissue enriched | √ |
| IQCF3 | ENSG00000229972 | Tissue enriched | √ |
| AC083862.1 | ENSG00000229974 | Tissue enriched | × |
| AC006455.1 | ENSG00000230000 | Tissue enriched | × |
| POTEB2 | ENSG00000230031 | Tissue enriched | × |
| ANKRD66 | ENSG00000230062 | Tissue enriched | × |
| CT47A8 | ENSG00000230347 | Tissue enriched | × |
| CT47A4 | ENSG00000230594 | Tissue enriched | × |
| PRB4 | ENSG00000230657 | Tissue enriched | √ |
| LINC00692 | ENSG00000230891 | Tissue enriched | × |
| LINC01098 | ENSG00000231171 | Tissue enriched | × |
| C17orf105 | ENSG00000231256 | Tissue enriched | √ |
| SBK3 | ENSG00000231274 | Tissue enriched | × |
| C18orf42 | ENSG00000231824 | Tissue enriched | × |
| GAGE2B | ENSG00000231850 | Tissue enriched | √ |
| CYP21A2 | ENSG00000231852 | Tissue enriched | √ |
| PRH1 | ENSG00000231887 | Tissue enriched | × |
| PSG1 | ENSG00000231924 | Tissue enriched | √ |
| SCAND3 | ENSG00000232040 | Tissue enriched | √ |
| CT45A3 | ENSG00000232417 | Tissue enriched | √ |
| CT45A1 | ENSG00000232478 | Tissue enriched | √ |
| AL513478.1 | ENSG00000232866 | Tissue enriched | × |
| NPIPB7 | ENSG00000233232 | Tissue enriched | × |
| BTBD18 | ENSG00000233436 | Tissue enriched | √ |
| AC109829.1 | ENSG00000233438 | Tissue enriched | × |
| PRR23C | ENSG00000233701 | Tissue enriched | √ |
| SPATA31A5 | ENSG00000233788 | Tissue enriched | × |
| TSPY4 | ENSG00000233803 | Tissue enriched | √ |
| AC008394.1 | ENSG00000233828 | Tissue enriched | × |
| POTEB | ENSG00000233917 | Tissue enriched | √ |
| PAGE2 | ENSG00000234068 | Tissue enriched | √ |
| SPATA31A4 | ENSG00000234214 | Tissue enriched | × |
| AC006547.14 | ENSG00000234409 | Tissue enriched | × |
| RBMY1A1 | ENSG00000234414 | Tissue enriched | √ |
| KBTBD13 | ENSG00000234438 | Tissue enriched | √ |
| AC002365.1 | ENSG00000234469 | Tissue enriched | × |
| C5orf58 | ENSG00000234511 | Tissue enriched | √ |
| SPATA31A7 | ENSG00000234734 | Tissue enriched | × |
| C11orf94 | ENSG00000234776 | Tissue enriched | √ |
| APOC2 | ENSG00000234906 | Tissue enriched | √ |
| TMEM30C | ENSG00000235156 | Tissue enriched | √ |
| AC006946.15 | ENSG00000235478 | Tissue enriched | × |
| SPANXB1 | ENSG00000235604 | Tissue enriched | × |
| RNF148 | ENSG00000235631 | Tissue enriched | √ |
| AL133481.1 | ENSG00000235705 | Tissue enriched | × |
| C10orf40 | ENSG00000235931 | Tissue enriched | √ |
| LCE6A | ENSG00000235942 | Tissue enriched | √ |
| CT47A3 | ENSG00000236126 | Tissue enriched | × |
| GAGE2C | ENSG00000236249 | Tissue enriched | √ |
| CLEC2L | ENSG00000236279 | Tissue enriched | √ |
| SLFN14 | ENSG00000236320 | Tissue enriched | √ |
| GAGE12F | ENSG00000236362 | Tissue enriched | √ |
| CT47A1 | ENSG00000236371 | Tissue enriched | √ |
| TSPY10 | ENSG00000236424 | Tissue enriched | × |
| CT47B1 | ENSG00000236446 | Tissue enriched | √ |
| GAGE12B | ENSG00000236737 | Tissue enriched | × |
| C5orf27 | ENSG00000236882 | Tissue enriched | √ |
| C3orf84 | ENSG00000236980 | Tissue enriched | × |
| C4orf51 | ENSG00000237136 | Tissue enriched | √ |
| PRSS56 | ENSG00000237412 | Tissue enriched | × |
| RP11×147C23.1 | ENSG00000237435 | Tissue enriched | × |
| AC074212.3 | ENSG00000237452 | Tissue enriched | × |
| OR7E24 | ENSG00000237521 | Tissue enriched | √ |
| AC114783.1 | ENSG00000237524 | Tissue enriched | × |
| GAGE13 | ENSG00000237597 | Tissue enriched | √ |
| GAGE12C | ENSG00000237671 | Tissue enriched | × |
| AC005008.2 | ENSG00000237896 | Tissue enriched | × |
| CT47A5 | ENSG00000237957 | Tissue enriched | × |
| TSPY6P | ENSG00000238074 | Tissue enriched | × |
| PAGE2B | ENSG00000238269 | Tissue enriched | √ |
| ASB14 | ENSG00000239388 | Tissue enriched | √ |
| KLHL41 | ENSG00000239474 | Tissue enriched | × |
| POM121L7 | ENSG00000239511 | Tissue enriched | × |
| C2orf61 | ENSG00000239605 | Tissue enriched | √ |
| DEFA3 | ENSG00000239839 | Tissue enriched | × |
| TEX35 | ENSG00000240021 | Tissue enriched | × |
| AMY2B | ENSG00000240038 | Tissue enriched | √ |
| SMKR1 | ENSG00000240204 | Tissue enriched | × |
| DEFA1B | ENSG00000240247 | Tissue enriched | √ |
| GAGE2D | ENSG00000240257 | Tissue enriched | √ |
| LCE1F | ENSG00000240386 | Tissue enriched | √ |
| PNMA2 | ENSG00000240694 | Tissue enriched | √ |
| LRRD1 | ENSG00000240720 | Tissue enriched | × |
| KRBOX1 | ENSG00000240747 | Tissue enriched | × |
| PCDHGC5 | ENSG00000240764 | Tissue enriched | √ |
| UGT1A9 | ENSG00000241119 | Tissue enriched | × |
| OR14A2 | ENSG00000241128 | Tissue enriched | × |
| GAGE12I | ENSG00000241465 | Tissue enriched | × |
| SSX2 | ENSG00000241476 | Tissue enriched | √ |
| CORT | ENSG00000241563 | Tissue enriched | √ |
| TMEFF1 | ENSG00000241697 | Tissue enriched | √ |
| CT45A2 | ENSG00000242185 | Tissue enriched | √ |
| TCP10L | ENSG00000242220 | Tissue enriched | √ |
| PSG2 | ENSG00000242221 | Tissue enriched | √ |
| CT45A5 | ENSG00000242284 | Tissue enriched | √ |
| CT47A2 | ENSG00000242362 | Tissue enriched | √ |
| RBMY1E | ENSG00000242389 | Tissue enriched | √ |
| PCDHGC4 | ENSG00000242419 | Tissue enriched | √ |
| GTF2A1L | ENSG00000242441 | Tissue enriched | √ |
| SERPINB10 | ENSG00000242550 | Tissue enriched | √ |
| CCDC169 | ENSG00000242715 | Tissue enriched | × |
| RBMY1B | ENSG00000242875 | Tissue enriched | √ |
| ERVW×1 | ENSG00000242950 | Tissue enriched | × |
| PSG11 | ENSG00000243130 | Tissue enriched | √ |
| UGT1A3 | ENSG00000243135 | Tissue enriched | × |
| PSG4 | ENSG00000243137 | Tissue enriched | √ |
| AMY2A | ENSG00000243480 | Tissue enriched | √ |
| CFB | ENSG00000243649 | Tissue enriched | √ |
| RGAG1 | ENSG00000243978 | Tissue enriched | √ |
| LCE3C | ENSG00000244057 | Tissue enriched | √ |
| UGT1A7 | ENSG00000244122 | Tissue enriched | √ |
| CFB | ENSG00000244255 | Tissue enriched | √ |
| RBMY1D | ENSG00000244395 | Tissue enriched | × |
| KRTAP5×7 | ENSG00000244411 | Tissue enriched | √ |
| CFHR1 | ENSG00000244414 | Tissue enriched | √ |
| UGT1A4 | ENSG00000244474 | Tissue enriched | √ |
| ERVFRD×1 | ENSG00000244476 | Tissue enriched | × |
| RAD21L1 | ENSG00000244588 | Tissue enriched | √ |
| ASPRV1 | ENSG00000244617 | Tissue enriched | √ |
| HBB | ENSG00000244734 | Tissue enriched | √ |
| CEBPA | ENSG00000245848 | Tissue enriched | √ |
| INSL3 | ENSG00000248099 | Tissue enriched | √ |
| TRIM39×RPP21 | ENSG00000248167 | Tissue enriched | × |
| TARM1 | ENSG00000248385 | Tissue enriched | √ |
| POU5F2 | ENSG00000248483 | Tissue enriched | √ |
| RP11×766F14.2 | ENSG00000248713 | Tissue enriched | × |
| EPPIN×WFDC6 | ENSG00000249139 | Tissue enriched | × |
| THEGL | ENSG00000249693 | Tissue enriched | × |
| LGALS16 | ENSG00000249861 | Tissue enriched | × |
| RNF103×CHMP3 | ENSG00000249884 | Tissue enriched | × |
| CCDC79 | ENSG00000249961 | Tissue enriched | √ |
| RP11×180C1.1 | ENSG00000250038 | Tissue enriched | × |
| KIAA1456 | ENSG00000250305 | Tissue enriched | × |
| GYPB | ENSG00000250361 | Tissue enriched | √ |
| KIAA1210 | ENSG00000250423 | Tissue enriched | √ |
| MEGT1 | ENSG00000250641 | Tissue enriched | × |
| CCDC169×SOHLH2 | ENSG00000250709 | Tissue enriched | × |
| NT5C1B×RDH14 | ENSG00000250741 | Tissue enriched | × |
| RFPL4B | ENSG00000251258 | Tissue enriched | √ |
| RP11×171N4.2 | ENSG00000251283 | Tissue enriched | × |
| PRB1 | ENSG00000251655 | Tissue enriched | √ |
| SMIM18 | ENSG00000253457 | Tissue enriched | × |
| NACA2 | ENSG00000253506 | Tissue enriched | √ |
| RP11×404L6.2 | ENSG00000253787 | Tissue enriched | × |
| PCDHGA10 | ENSG00000253846 | Tissue enriched | √ |
| RP11×386G21.2 | ENSG00000253857 | Tissue enriched | × |
| PCDHGB1 | ENSG00000254221 | Tissue enriched | √ |
| INS | ENSG00000254647 | Tissue enriched | √ |
| AP000783.1 | ENSG00000254667 | Tissue enriched | × |
| EID3 | ENSG00000255150 | Tissue enriched | √ |
| PRR23D1 | ENSG00000255251 | Tissue enriched | × |
| OR8G5 | ENSG00000255298 | Tissue enriched | √ |
| CCDC179 | ENSG00000255359 | Tissue enriched | × |
| PRR23D2 | ENSG00000255378 | Tissue enriched | × |
| CARD18 | ENSG00000255501 | Tissue enriched | √ |
| NPIPB8 | ENSG00000255524 | Tissue enriched | × |
| RAB44 | ENSG00000255587 | Tissue enriched | × |
| RP11×234B24.6 | ENSG00000255639 | Tissue enriched | × |
| RP4×559A3.7 | ENSG00000255835 | Tissue enriched | × |
| CYP2A6 | ENSG00000255974 | Tissue enriched | √ |
| PAPPA×AS1 | ENSG00000256040 | Tissue enriched | × |
| LINC00696 | ENSG00000256097 | Tissue enriched | × |
| HMBS | ENSG00000256269 | Tissue enriched | √ |
| DND1 | ENSG00000256453 | Tissue enriched | × |
| PGA5 | ENSG00000256713 | Tissue enriched | √ |
| SLC5A8 | ENSG00000256870 | Tissue enriched | √ |
| KHDC1L | ENSG00000256980 | Tissue enriched | √ |
| HP | ENSG00000257017 | Tissue enriched | √ |
| C12orf79 | ENSG00000257242 | Tissue enriched | × |
| RP11×762I7.5 | ENSG00000257390 | Tissue enriched | × |
| OVCH1×AS1 | ENSG00000257599 | Tissue enriched | × |
| RP11×552I14.1 | ENSG00000257860 | Tissue enriched | × |
| LINC00935 | ENSG00000257987 | Tissue enriched | × |
| PRSS58 | ENSG00000258223 | Tissue enriched | × |
| RP11×362K2.2 | ENSG00000258231 | Tissue enriched | × |
| RP11×219B4.5 | ENSG00000258256 | Tissue enriched | × |
| RP11×536G4.1 | ENSG00000258292 | Tissue enriched | × |
| SPESP1 | ENSG00000258484 | Tissue enriched | √ |
| C20orf141 | ENSG00000258713 | Tissue enriched | √ |
| RP11×463J10.2 | ENSG00000258928 | Tissue enriched | × |
| TSPY1 | ENSG00000258992 | Tissue enriched | √ |
| RP11×371E8.4 | ENSG00000259066 | Tissue enriched | × |
| SLC35G6 | ENSG00000259224 | Tissue enriched | × |
| CTD×2116N17.1 | ENSG00000259316 | Tissue enriched | × |
| GH1 | ENSG00000259384 | Tissue enriched | √ |
| RP11×467N20.5 | ENSG00000259455 | Tissue enriched | × |
| RP11×324D17.1 | ENSG00000259517 | Tissue enriched | × |
| RP11×597K23.2 | ENSG00000259518 | Tissue enriched | × |
| C6orf229 | ENSG00000260286 | Tissue enriched | × |
| RP11×1035H13.3 | ENSG00000260342 | Tissue enriched | × |
| C16orf95 | ENSG00000260456 | Tissue enriched | × |
| XKR7 | ENSG00000260903 | Tissue enriched | √ |
| RP11×697E2.6 | ENSG00000261147 | Tissue enriched | × |
| C16orf97 | ENSG00000261190 | Tissue enriched | × |
| MUC22 | ENSG00000261272 | Tissue enriched | × |
| TP53TG3B | ENSG00000261509 | Tissue enriched | √ |
| GAN | ENSG00000261609 | Tissue enriched | √ |
| HPR | ENSG00000261701 | Tissue enriched | √ |
| RP11×676J12.7 | ENSG00000262003 | Tissue enriched | × |
| C19orf84 | ENSG00000262874 | Tissue enriched | × |
| MYZAP | ENSG00000263155 | Tissue enriched | × |
| RP11×321M21.3 | ENSG00000264116 | Tissue enriched | × |
| MYH4 | ENSG00000264424 | Tissue enriched | √ |
| RP11×723G8.2 | ENSG00000265352 | Tissue enriched | × |
| RP11×41O4.1 | ENSG00000266258 | Tissue enriched | × |
| TBC1D29 | ENSG00000266733 | Tissue enriched | √ |
| CTB×186H2.3 | ENSG00000266956 | Tissue enriched | × |
| RP11×886H22.1 | ENSG00000266997 | Tissue enriched | × |
| APOC4 | ENSG00000267467 | Tissue enriched | √ |
| CTD×2528L19.4 | ENSG00000267552 | Tissue enriched | × |
| CGB1 | ENSG00000267631 | Tissue enriched | √ |
| AC006116.20 | ENSG00000267710 | Tissue enriched | × |
| AL118506.1 | ENSG00000267848 | Tissue enriched | × |
| PRED58 | ENSG00000267857 | Tissue enriched | × |
| AL031666.2 | ENSG00000267882 | Tissue enriched | × |
| AC096582.1 | ENSG00000267988 | Tissue enriched | × |
| AC025262.1 | ENSG00000268028 | Tissue enriched | × |
| AC078925.1 | ENSG00000268091 | Tissue enriched | × |
| HBCBP | ENSG00000268162 | Tissue enriched | × |
| SMIM17 | ENSG00000268182 | Tissue enriched | × |
| C15ORF31 | ENSG00000268327 | Tissue enriched | × |
| AL590714.1 | ENSG00000268387 | Tissue enriched | × |
| AC008948.1 | ENSG00000268424 | Tissue enriched | × |
| BX088651.2 | ENSG00000268708 | Tissue enriched | × |
| AL590235.1 | ENSG00000268781 | Tissue enriched | × |
| AC132872.2 | ENSG00000268852 | Tissue enriched | × |
| ERVV×2 | ENSG00000268964 | Tissue enriched | × |
| CALR3 | ENSG00000269058 | Tissue enriched | √ |
| AC005606.1 | ENSG00000269205 | Tissue enriched | × |
| AC020629.1 | ENSG00000269367 | Tissue enriched | × |
| ERVV×1 | ENSG00000269526 | Tissue enriched | × |
| AL590560.1 | ENSG00000269620 | Tissue enriched | × |
| KLK9 | ENSG00000269741 | Tissue enriched | √ |
| AC015660.1 | ENSG00000269810 | Tissue enriched | × |
| MROH7×TTC4 | ENSG00000271723 | Tissue enriched | × |
| DKFZP761K2322 | ENSG00000272268 | Tissue enriched | × |
| MUSTN1 | ENSG00000272573 | Tissue enriched | √ |
| RP11×155D18.14 | ENSG00000272762 | Tissue enriched | × |
| GRIN2B | ENSG00000273079 | Tissue enriched | √ |
| AC005358.1 | ENSG00000273452 | Tissue enriched | × |
