# Supplementary material for: A Computational Method for Classifying Different Human Tissues with Quantitatively Tissue-Specific Expressed Genes
Source: Genes (Basel). 2018 Sep 7;9(9):449. doi: 10.3390/genes9090449 (PMC6162521; doi:10.3390/genes9090449)
Supplement: Supplementary file 1 [file genes-09-00449-s001.zip › Supplementary-Material-S5.docx]

**Supplementary Material S5.** Significantly highly expressed genes in each tissue obtained by the t test between one tissue and all other tissues.

| **Gene symbol** | **Rank** | **Tag of tissue** |
| --- | --- | --- |
| SEC11A | 1 | *T*_1_ |
| TSPAN4 | 2 | *T*_1_ |
| FGD5 | 3 | *T*_1_ |
| PLXND1 | 4 | *T*_1_ |
| TGFBR2 | 5 | *T*_1_ |
| STARD8 | 6 | *T*_1_ |
| EHD2 | 7 | *T*_1_ |
| LEPROT | 8 | *T*_1_ |
| LAMC1 | 9 | *T*_1_ |
| SHE | 10 | *T*_1_ |
| TSPAN14 | 11 | *T*_1_ |
| RAP1B | 12 | *T*_1_ |
| AFAP1L1 | 13 | *T*_1_ |
| MMRN2 | 14 | *T*_1_ |
| SLC25A24 | 15 | *T*_1_ |
| SDCBP | 16 | *T*_1_ |
| ACVRL1 | 17 | *T*_1_ |
| EPS8 | 18 | *T*_1_ |
| MAP7D3 | 19 | *T*_1_ |
| CHRNB1 | 1 | *T*_2_ |
| RER1 | 2 | *T*_2_ |
| MPDU1 | 3 | *T*_2_ |
| UROD | 4 | *T*_2_ |
| INTS9 | 5 | *T*_2_ |
| NR1H4 | 6 | *T*_2_ |
| CNPY2 | 7 | *T*_2_ |
| ACP1 | 8 | *T*_2_ |
| FAH | 9 | *T*_2_ |
| POLDIP2 | 10 | *T*_2_ |
| UBA5 | 11 | *T*_2_ |
| MPV17 | 12 | *T*_2_ |
| SLC46A1 | 13 | *T*_2_ |
| CUTA | 14 | *T*_2_ |
| GRTP1-AS1 | 15 | *T*_2_ |
| PARP3 | 16 | *T*_2_ |
| SRSF9 | 17 | *T*_2_ |
| AKR1B1 | 18 | *T*_2_ |
| ATR | 19 | *T*_2_ |
| DOCK2 | 1 | *T*_3_ |
| SASH3 | 2 | *T*_3_ |
| RCSD1 | 3 | *T*_3_ |
| NCKAP1L | 4 | *T*_3_ |
| RAC2 | 5 | *T*_3_ |
| RAB8A | 6 | *T*_3_ |
| STK4 | 7 | *T*_3_ |
| RINL | 8 | *T*_3_ |
| NDE1 | 9 | *T*_3_ |
| CXorf38 | 10 | *T*_3_ |
| ARID3B | 11 | *T*_3_ |
| MICB | 12 | *T*_3_ |
| KCNAB2 | 13 | *T*_3_ |
| VTI1A | 14 | *T*_3_ |
| CYFIP2 | 15 | *T*_3_ |
| RFFL | 16 | *T*_3_ |
| ERP44 | 17 | *T*_3_ |
| PRKCB | 18 | *T*_3_ |
| IFNAR2 | 19 | *T*_3_ |
| ATL3 | 1 | *T*_4_ |
| CRTAP | 2 | *T*_4_ |
| FAM127C | 3 | *T*_4_ |
| FGFR1 | 4 | *T*_4_ |
| FKBP7 | 5 | *T*_4_ |
| LIMS2 | 6 | *T*_4_ |
| LIX1L | 7 | *T*_4_ |
| MRVI1 | 8 | *T*_4_ |
| RAB34 | 9 | *T*_4_ |
| RBMS3 | 10 | *T*_4_ |
| RSU1 | 11 | *T*_4_ |
| SGCA | 12 | *T*_4_ |
| SNX9 | 13 | *T*_4_ |
| SYDE1 | 14 | *T*_4_ |
| TGFB1I1 | 15 | *T*_4_ |
| TMEM109 | 16 | *T*_4_ |
| TMEM43 | 17 | *T*_4_ |
| WWTR1 | 18 | *T*_4_ |
| MXRA7 | 19 | *T*_4_ |
| AARS | 1 | *T*_5_ |
| AASDHPPT | 2 | *T*_5_ |
| ABAT | 3 | *T*_5_ |
| ABCB9 | 4 | *T*_5_ |
| ABHD10 | 5 | *T*_5_ |
| ABHD12 | 6 | *T*_5_ |
| ABHD14A | 7 | *T*_5_ |
| ABHD8 | 8 | *T*_5_ |
| ABI2 | 9 | *T*_5_ |
| AC004221.2 | 10 | *T*_5_ |
| AC005197.2 | 11 | *T*_5_ |
| AC005330.2 | 12 | *T*_5_ |
| AC006115.3 | 13 | *T*_5_ |
| AC006538.1 | 14 | *T*_5_ |
| AC007092.1 | 15 | *T*_5_ |
| AC018730.1 | 16 | *T*_5_ |
| AC018730.3 | 17 | *T*_5_ |
| AC068057.2 | 18 | *T*_5_ |
| AC084219.4 | 19 | *T*_5_ |
| RPS20 | 1 | *T*_6_ |
| RPL23 | 2 | *T*_6_ |
| SEC11A | 3 | *T*_6_ |
| LEPROT | 4 | *T*_6_ |
| RPL11 | 5 | *T*_6_ |
| TSPAN4 | 6 | *T*_6_ |
| TCF7L2 | 7 | *T*_6_ |
| TCF7L1 | 8 | *T*_6_ |
| ECE1 | 9 | *T*_6_ |
| ODF2L | 10 | *T*_6_ |
| NFIB | 11 | *T*_6_ |
| RPL7 | 12 | *T*_6_ |
| C5orf38 | 13 | *T*_6_ |
| PKN3 | 14 | *T*_6_ |
| RPL10 | 15 | *T*_6_ |
| LGALS3 | 16 | *T*_6_ |
| ABHD14B | 17 | *T*_6_ |
| RNASE4 | 18 | *T*_6_ |
| LHFP | 19 | *T*_6_ |
| TMEM51 | 1 | *T*_7_ |
| SRC | 2 | *T*_7_ |
| MLXIP | 3 | *T*_7_ |
| PFN1 | 4 | *T*_7_ |
| HOXA2 | 5 | *T*_7_ |
| VASP | 6 | *T*_7_ |
| HEPH | 7 | *T*_7_ |
| TSPAN15 | 8 | *T*_7_ |
| HOXA9 | 9 | *T*_7_ |
| CHMP4B | 10 | *T*_7_ |
| HOXB3 | 11 | *T*_7_ |
| HOXA10 | 12 | *T*_7_ |
| NEURL1B | 13 | *T*_7_ |
| WNK2 | 14 | *T*_7_ |
| HOXA11-AS | 15 | *T*_7_ |
| ZDHHC7 | 16 | *T*_7_ |
| C1orf226 | 17 | *T*_7_ |
| SCN9A | 18 | *T*_7_ |
| HOXA3 | 19 | *T*_7_ |
| TRIOBP | 1 | *T*_8_ |
| HOXA-AS2 | 2 | *T*_8_ |
| DYNLT1 | 3 | *T*_8_ |
| FAM127B | 4 | *T*_8_ |
| TUBB6 | 5 | *T*_8_ |
| TMEM109 | 6 | *T*_8_ |
| TRAF7 | 7 | *T*_8_ |
| C6orf47 | 8 | *T*_8_ |
| LRP10 | 9 | *T*_8_ |
| DAAM1 | 10 | *T*_8_ |
| TPD52L2 | 11 | *T*_8_ |
| RP11-834C11.7 | 12 | *T*_8_ |
| ZNF503-AS2 | 13 | *T*_8_ |
| FAM127C | 14 | *T*_8_ |
| TRIP6 | 15 | *T*_8_ |
| C9orf3 | 16 | *T*_8_ |
| 10-Sep | 17 | *T*_8_ |
| HOXA2 | 18 | *T*_8_ |
| PDCD7 | 19 | *T*_8_ |
| TNNT2 | 1 | *T*_9_ |
| FABP3 | 2 | *T*_9_ |
| TPM1 | 3 | *T*_9_ |
| GATA4 | 4 | *T*_9_ |
| TCAP | 5 | *T*_9_ |
| TNNC1 | 6 | *T*_9_ |
| NDUFA12 | 7 | *T*_9_ |
| GATA6 | 8 | *T*_9_ |
| AFAP1L1 | 9 | *T*_9_ |
| CKM | 10 | *T*_9_ |
| PRKACA | 11 | *T*_9_ |
| MYBPC3 | 12 | *T*_9_ |
| SGCA | 13 | *T*_9_ |
| ALDOA | 14 | *T*_9_ |
| MB | 15 | *T*_9_ |
| NDUFS5 | 16 | *T*_9_ |
| MINOS1 | 17 | *T*_9_ |
| NDUFA8 | 18 | *T*_9_ |
| SLC4A3 | 19 | *T*_9_ |
| SERPINF2 | 1 | *T*_10_ |
| VTN | 2 | *T*_10_ |
| APCS | 3 | *T*_10_ |
| ASGR2 | 4 | *T*_10_ |
| MASP2 | 5 | *T*_10_ |
| ITIH4 | 6 | *T*_10_ |
| ASGR1 | 7 | *T*_10_ |
| SEPHS2 | 8 | *T*_10_ |
| NR1H4 | 9 | *T*_10_ |
| HYAL1 | 10 | *T*_10_ |
| TMEM176B | 11 | *T*_10_ |
| CISD3 | 12 | *T*_10_ |
| MARVELD2 | 13 | *T*_10_ |
| C8G | 14 | *T*_10_ |
| SLC35D2 | 15 | *T*_10_ |
| SLC7A9 | 16 | *T*_10_ |
| CAMSAP3 | 17 | *T*_10_ |
| SPRYD4 | 18 | *T*_10_ |
| FDX1 | 19 | *T*_10_ |
| TAGLN2 | 1 | *T*_11_ |
| TRPV2 | 2 | *T*_11_ |
| CD4 | 3 | *T*_11_ |
| PLXND1 | 4 | *T*_11_ |
| GPR116 | 5 | *T*_11_ |
| PAPSS2 | 6 | *T*_11_ |
| RPS6KA1 | 7 | *T*_11_ |
| VAMP8 | 8 | *T*_11_ |
| ESAM | 9 | *T*_11_ |
| CCND3 | 10 | *T*_11_ |
| FHOD1 | 11 | *T*_11_ |
| TMEM173 | 12 | *T*_11_ |
| PIEZO1 | 13 | *T*_11_ |
| MYO1B | 14 | *T*_11_ |
| UNC13D | 15 | *T*_11_ |
| F11R | 16 | *T*_11_ |
| RBMS2 | 17 | *T*_11_ |
| TBX4 | 18 | *T*_11_ |
| FGD5 | 19 | *T*_11_ |
| POLDIP2 | 1 | *T*_12_ |
| YBX1 | 2 | *T*_12_ |
| FAF1 | 3 | *T*_12_ |
| LRRC47 | 4 | *T*_12_ |
| CUL3 | 5 | *T*_12_ |
| C19orf47 | 6 | *T*_12_ |
| HRC | 7 | *T*_12_ |
| RAPSN | 8 | *T*_12_ |
| ADSL | 9 | *T*_12_ |
| FXR1 | 10 | *T*_12_ |
| SGCA | 11 | *T*_12_ |
| HOXC9 | 12 | *T*_12_ |
| EPM2A | 13 | *T*_12_ |
| DNAJB6 | 14 | *T*_12_ |
| CUL4A | 15 | *T*_12_ |
| CEP63 | 16 | *T*_12_ |
| PRKRA | 17 | *T*_12_ |
| RRAGD | 18 | *T*_12_ |
| WBSCR22 | 19 | *T*_12_ |
| ANXA2 | 1 | *T*_13_ |
| LEPRE1 | 2 | *T*_13_ |
| SORBS3 | 3 | *T*_13_ |
| CNTLN | 4 | *T*_13_ |
| CD81 | 5 | *T*_13_ |
| LPAR1 | 6 | *T*_13_ |
| TMEM109 | 7 | *T*_13_ |
| RP11-834C11.4 | 8 | *T*_13_ |
| ARHGEF28 | 9 | *T*_13_ |
| EBF2 | 10 | *T*_13_ |
| BMPR1B | 11 | *T*_13_ |
| S100A10 | 12 | *T*_13_ |
| FER | 13 | *T*_13_ |
| RAB34 | 14 | *T*_13_ |
| RBMS3 | 15 | *T*_13_ |
| ARL2 | 16 | *T*_13_ |
| ITPR3 | 17 | *T*_13_ |
| CAPNS1 | 18 | *T*_13_ |
| ACSS3 | 19 | *T*_13_ |
| RPS10 | 1 | *T*_14_ |
| RPL23 | 2 | *T*_14_ |
| RAB34 | 3 | *T*_14_ |
| RPS3 | 4 | *T*_14_ |
| HNRNPA1 | 5 | *T*_14_ |
| BST2 | 6 | *T*_14_ |
| IMPDH2 | 7 | *T*_14_ |
| RPL36A | 8 | *T*_14_ |
| RPS3A | 9 | *T*_14_ |
| NR5A1 | 10 | *T*_14_ |
| FBL | 11 | *T*_14_ |
| BNC2 | 12 | *T*_14_ |
| GNB5 | 13 | *T*_14_ |
| RPS5 | 14 | *T*_14_ |
| RPS24 | 15 | *T*_14_ |
| RPL11 | 16 | *T*_14_ |
| RPL15 | 17 | *T*_14_ |
| ZNF618 | 18 | *T*_14_ |
| TTC28 | 19 | *T*_14_ |
| GRHL2 | 1 | *T*_15_ |
| EPCAM | 2 | *T*_15_ |
| C1orf172 | 3 | *T*_15_ |
| GNB2L1 | 4 | *T*_15_ |
| STXBP2 | 5 | *T*_15_ |
| AP1M2 | 6 | *T*_15_ |
| RPL18A | 7 | *T*_15_ |
| RPL35A | 8 | *T*_15_ |
| KRT18 | 9 | *T*_15_ |
| CRB3 | 10 | *T*_15_ |
| PNLIPRP1 | 11 | *T*_15_ |
| RPS3A | 12 | *T*_15_ |
| PRSS1 | 13 | *T*_15_ |
| SEC61A1 | 14 | *T*_15_ |
| EEF1B2 | 15 | *T*_15_ |
| HNF4G | 16 | *T*_15_ |
| CGN | 17 | *T*_15_ |
| LLGL2 | 18 | *T*_15_ |
| P4HB | 19 | *T*_15_ |
| BEX2 | 1 | *T*_16_ |
| GDI1 | 2 | *T*_16_ |
| SCAMP5 | 3 | *T*_16_ |
| BEX4 | 4 | *T*_16_ |
| CCDC181 | 5 | *T*_16_ |
| FAM120AOS | 6 | *T*_16_ |
| BEX1 | 7 | *T*_16_ |
| AP3B2 | 8 | *T*_16_ |
| TCEAL2 | 9 | *T*_16_ |
| B9D1 | 10 | *T*_16_ |
| MAPK10 | 11 | *T*_16_ |
| LINC00094 | 12 | *T*_16_ |
| CUTA | 13 | *T*_16_ |
| REEP2 | 14 | *T*_16_ |
| P4HTM | 15 | *T*_16_ |
| ATAT1 | 16 | *T*_16_ |
| OGDHL | 17 | *T*_16_ |
| CRMP1 | 18 | *T*_16_ |
| MAPRE3 | 19 | *T*_16_ |
| RPS4Y1 | 1 | *T*_17_ |
| RPL10A | 2 | *T*_17_ |
| USP9Y | 3 | *T*_17_ |
| HOXA13 | 4 | *T*_17_ |
| SRC | 5 | *T*_17_ |
| HDAC1 | 6 | *T*_17_ |
| RP11-475N22.4 | 7 | *T*_17_ |
| NXN | 8 | *T*_17_ |
| RPS8 | 9 | *T*_17_ |
| USP20 | 10 | *T*_17_ |
| KDM5D | 11 | *T*_17_ |
| CHD3 | 12 | *T*_17_ |
| MANBAL | 13 | *T*_17_ |
| HOXA10 | 14 | *T*_17_ |
| RPL7A | 15 | *T*_17_ |
| SCMH1 | 16 | *T*_17_ |
| RPS24 | 17 | *T*_17_ |
| NBL1 | 18 | *T*_17_ |
| FOXP4 | 19 | *T*_17_ |
| AMMECR1 | 1 | *T*_18_ |
| APLF | 2 | *T*_18_ |
| ARF6 | 3 | *T*_18_ |
| C5orf38 | 4 | *T*_18_ |
| CD44 | 5 | *T*_18_ |
| EGFR | 6 | *T*_18_ |
| EIF3B | 7 | *T*_18_ |
| EIF3D | 8 | *T*_18_ |
| FARP2 | 9 | *T*_18_ |
| GLI3 | 10 | *T*_18_ |
| GNAI3 | 11 | *T*_18_ |
| GNB2L1 | 12 | *T*_18_ |
| GTF3C4 | 13 | *T*_18_ |
| HIATL1 | 14 | *T*_18_ |
| KDM5B | 15 | *T*_18_ |
| LAMP1 | 16 | *T*_18_ |
| LMNA | 17 | *T*_18_ |
| METTL8 | 18 | *T*_18_ |
| NECAP2 | 19 | *T*_18_ |
| TMEM176B | 1 | *T*_19_ |
| HDAC1 | 2 | *T*_19_ |
| TTC31 | 3 | *T*_19_ |
| TNFRSF14 | 4 | *T*_19_ |
| TRIM14 | 5 | *T*_19_ |
| TEP1 | 6 | *T*_19_ |
| RAVER1 | 7 | *T*_19_ |
| RPS6KA3 | 8 | *T*_19_ |
| PARP12 | 9 | *T*_19_ |
| NFATC2IP | 10 | *T*_19_ |
| TMEM176A | 11 | *T*_19_ |
| PSME1 | 12 | *T*_19_ |
| HNRNPF | 13 | *T*_19_ |
| GSTK1 | 14 | *T*_19_ |
| GAK | 15 | *T*_19_ |
| ZDHHC6 | 16 | *T*_19_ |
| KHNYN | 17 | *T*_19_ |
| CNOT11 | 18 | *T*_19_ |
| RP11-693N9.2 | 19 | *T*_19_ |
| WAS | 1 | *T*_20_ |
| RASSF5 | 2 | *T*_20_ |
| PSMB8 | 3 | *T*_20_ |
| LSP1 | 4 | *T*_20_ |
| ARHGAP25 | 5 | *T*_20_ |
| GIT2 | 6 | *T*_20_ |
| ARHGAP30 | 7 | *T*_20_ |
| DOK1 | 8 | *T*_20_ |
| CYBA | 9 | *T*_20_ |
| VAV1 | 10 | *T*_20_ |
| CTC-241F20.3 | 11 | *T*_20_ |
| GAB3 | 12 | *T*_20_ |
| CMTM7 | 13 | *T*_20_ |
| DOCK2 | 14 | *T*_20_ |
| RGS19 | 15 | *T*_20_ |
| TAPBP | 16 | *T*_20_ |
| PARVG | 17 | *T*_20_ |
| STK10 | 18 | *T*_20_ |
| MFNG | 19 | *T*_20_ |
| TMEM51 | 1 | *T*_21_ |
| GALNT12 | 2 | *T*_21_ |
| C1orf226 | 3 | *T*_21_ |
| FAM109B | 4 | *T*_21_ |
| CHRM3 | 5 | *T*_21_ |
| FZD5 | 6 | *T*_21_ |
| ANKS6 | 7 | *T*_21_ |
| DNAJC1 | 8 | *T*_21_ |
| SRC | 9 | *T*_21_ |
| TMTC2 | 10 | *T*_21_ |
| PTPRK | 11 | *T*_21_ |
| ISL1 | 12 | *T*_21_ |
| AAMP | 13 | *T*_21_ |
| CASZ1 | 14 | *T*_21_ |
| ARHGEF18 | 15 | *T*_21_ |
| RNASE4 | 16 | *T*_21_ |
| NSMCE4A | 17 | *T*_21_ |
| MECOM | 18 | *T*_21_ |
| RP11-834C11.7 | 19 | *T*_21_ |
| STXBP2 | 1 | *T*_22_ |
| FBXO7 | 2 | *T*_22_ |
| RNF157 | 3 | *T*_22_ |
| SMC6 | 4 | *T*_22_ |
| TTLL4 | 5 | *T*_22_ |
| ZWINT | 6 | *T*_22_ |
| TPX2 | 7 | *T*_22_ |
| NR6A1 | 8 | *T*_22_ |
| ZNF280B | 9 | *T*_22_ |
| STK36 | 10 | *T*_22_ |
| PIAS4 | 11 | *T*_22_ |
| MORC2 | 12 | *T*_22_ |
| ORC6 | 13 | *T*_22_ |
| EZH2 | 14 | *T*_22_ |
| R3HCC1L | 15 | *T*_22_ |
| OARD1 | 16 | *T*_22_ |
| PASK | 17 | *T*_22_ |
| ARID3B | 18 | *T*_22_ |
| SLC5A6 | 19 | *T*_22_ |
| ESAM | 1 | *T*_23_ |
| IQCA1 | 2 | *T*_23_ |
| RP11-532F12.5 | 3 | *T*_23_ |
| CNKSR1 | 4 | *T*_23_ |
| 2-Mar | 5 | *T*_23_ |
| RAB11B | 6 | *T*_23_ |
| PARP8 | 7 | *T*_23_ |
| ANKS6 | 8 | *T*_23_ |
| C16orf58 | 9 | *T*_23_ |
| SYNE4 | 10 | *T*_23_ |
| IQCK | 11 | *T*_23_ |
| KDELR1 | 12 | *T*_23_ |
| TMC4 | 13 | *T*_23_ |
| PLEK2 | 14 | *T*_23_ |
| ARHGAP8 | 15 | *T*_23_ |
| CCL28 | 16 | *T*_23_ |
| RBM47 | 17 | *T*_23_ |
| RASEF | 18 | *T*_23_ |
| TMEM205 | 19 | *T*_23_ |
| RAB34 | 1 | *T*_24_ |
| EMX2OS | 2 | *T*_24_ |
| TTC28 | 3 | *T*_24_ |
| RP11-475N22.4 | 4 | *T*_24_ |
| ATP11C | 5 | *T*_24_ |
| EMX2 | 6 | *T*_24_ |
| STAT5B | 7 | *T*_24_ |
| RBFOX2 | 8 | *T*_24_ |
| MEIS2 | 9 | *T*_24_ |
| ZFX | 10 | *T*_24_ |
| WT1-AS | 11 | *T*_24_ |
| TGFB1I1 | 12 | *T*_24_ |
| CNTLN | 13 | *T*_24_ |
| MANBAL | 14 | *T*_24_ |
| LIX1L | 15 | *T*_24_ |
| KIAA1614 | 16 | *T*_24_ |
| SRC | 17 | *T*_24_ |
| LEPREL2 | 18 | *T*_24_ |
| RERG | 19 | *T*_24_ |
| PIAS3 | 1 | *T*_25_ |
| NXN | 2 | *T*_25_ |
| SRC | 3 | *T*_25_ |
| TRIP6 | 4 | *T*_25_ |
| NRBP1 | 5 | *T*_25_ |
| ISL1 | 6 | *T*_25_ |
| YAP1 | 7 | *T*_25_ |
| PTBP1 | 8 | *T*_25_ |
| HOXD10 | 9 | *T*_25_ |
| EFS | 10 | *T*_25_ |
| HOXA13 | 11 | *T*_25_ |
| DVL2 | 12 | *T*_25_ |
| MYOF | 13 | *T*_25_ |
| TRIOBP | 14 | *T*_25_ |
| FAM114A1 | 15 | *T*_25_ |
| GLI3 | 16 | *T*_25_ |
| JAG1 | 17 | *T*_25_ |
| ELK3 | 18 | *T*_25_ |
| SYPL1 | 19 | *T*_25_ |
